# Supplementary material for: Frequent transitions in self-assembly across the evolution of a central metabolic enzyme
Source: Nat Commun. 2024 Dec 3;15:10515. doi: 10.1038/s41467-024-54408-6 (PMC11615384; doi:10.1038/s41467-024-54408-6)
Supplement: Supplementary file 1 — Supplementary Information [file 41467_2024_54408_MOESM1_ESM.pdf]

Supplementary Information:

Frequent transitions in self-assembly across the evolution of a central metabolic enzyme

Franziska L. Sendker[1]§, Tabea Schlotthauer[1]§, Christopher-Nils Mais[2]+, Yat Kei Lo[2]+, Mathias Girbig[1], Stefan Bohn[3], Thomas Heimerl[2], Daniel Schindler[2,4], Arielle Weinstein[5], Brian P. Metzger[5], Joseph W. Thornton[5,6], Arvind Pillai[5], Gert Bange[1,2,7], Jan M. Schuller[2,7], Georg K.A. Hochberg[1,2,7]*

1 Max-Planck-Institute for Terrestrial Microbiology; Karl-von-Frisch-Str. 10, 35043 Marburg, Germany

2 Center for Synthetic Microbiology (SYNMIKRO), Philipps-University Marburg; Karl-von-Frisch-Str. 14, 35043 Marburg, Germany

3 Institute of Structural Biology, Helmholtz Center Munich, Ingolstädter Landstraße 1 Neuherberg, Germany

4 MaxGENESYS Biofoundry, Max-Planck-Institute for Terrestrial Microbiology; Karl-von-Frisch-Str. 10, 35043 Marburg, Germany

5 Department of Ecology and Evolution, University of Chicago, Chicago, IL, USA

6 Department of Human Genetics, University of Chicago, Chicago, IL, USA

7 Department of Chemistry, Philipps-University Marburg; Hans-Meerwein-Str. 4, 35043 Marburg, Germany

§ These authors contributed equally. + These authors contributed equally. * Correspondence to georg.hochberg@mpi-marburg.mpg.de

This document contains

- Supplementary Figures S1-14
- Supplementary Tables S1-4

A list of DNA sequences of all proteins used in this study is provided as Supplementary Data File 1.

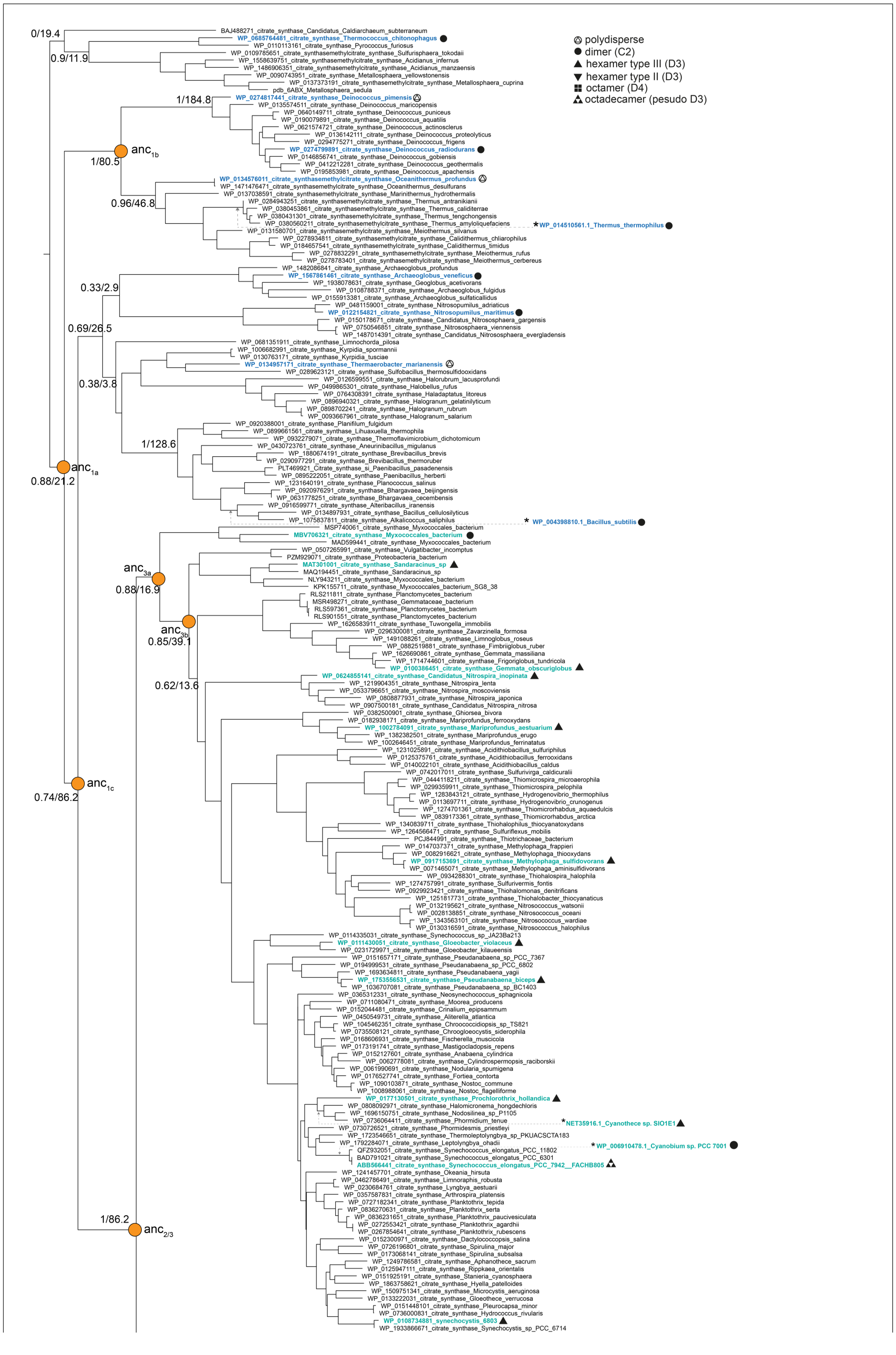

polydisperse
dimer (C2)
hexamer type III (D3)
hexamer type II (D3)
octamer (D4)
octadecamer (pesudo D3)
0/19.4
0.9/11.9
1/184.8
anc1b
1/80.5
0.96/46.8
0.33/2.9
0.69/26.5
0.38/3.8
1/128.6
anc1a
0.88/21.2
anc3a
0.88/16.9
anc3b
0.85/39.1
0.62/13.6
anc1c
0.74/86.2
1/86.2
anc2/3

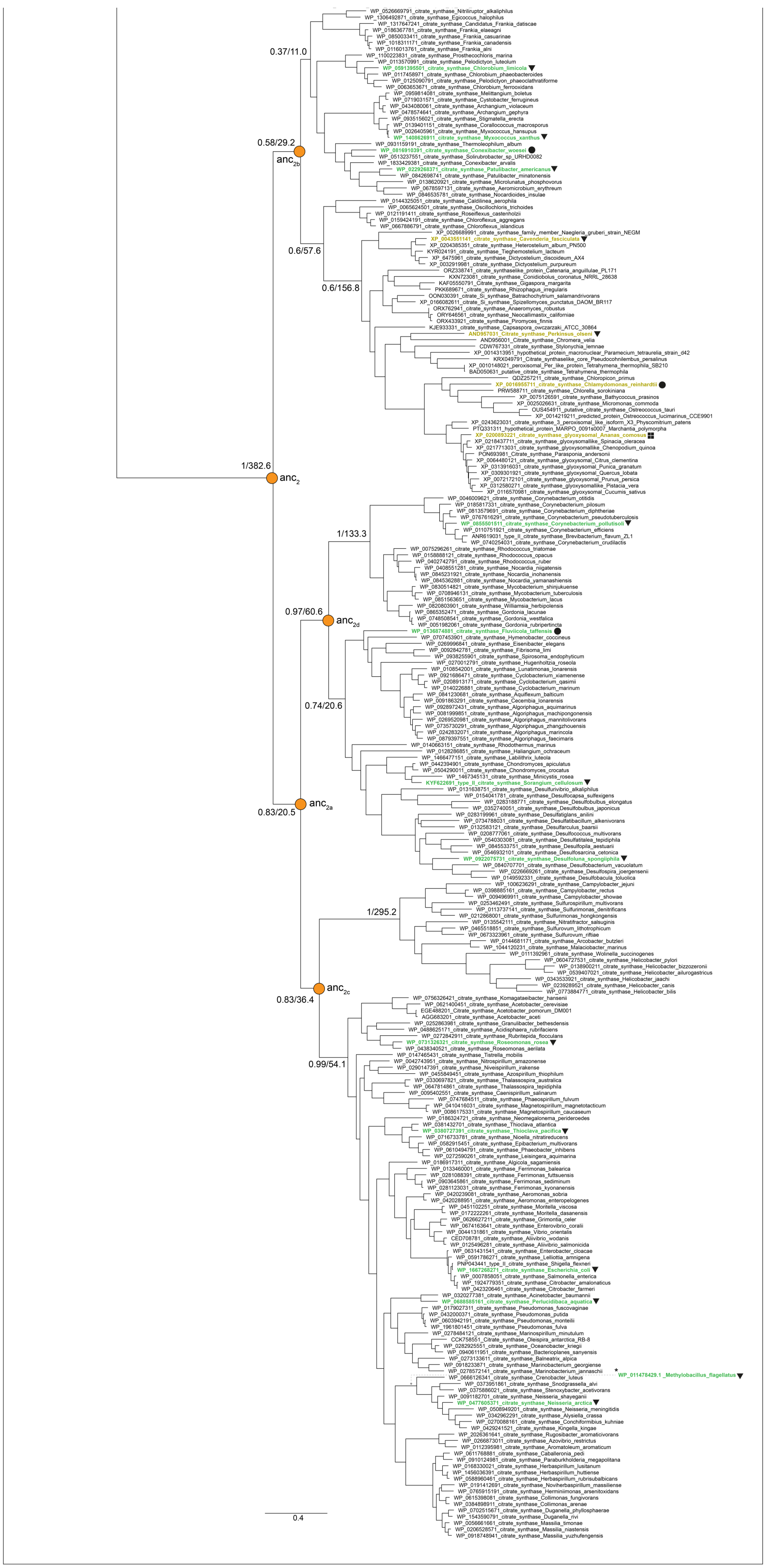

0.37/11.0
0.58/29.2
anc_{2b}
0.6/57.6
0.6/156.8
1/382.6
anc_2
1/133.3
0.97/60.6
anc_{2d}
0.74/20.6
anc_{2a}
0.83/20.5
1/295.2
anc_{2c}
0.83/36.4
0.99/54.1
0.4

Supplementary Fig. 1 Phylogenetic tree of citrate synthase Full tree with all sequences (organism name and NCBI-identifier) to infer the evolutionary history of citrate synthase (CS) in Bacteria, Archaea and non-mitochondrial CS in Eukaryotes. Extant sequences that were purified are colored and their quaternary structure is indicated with a symbol. Internal nodes of the tree for which ancestral sequences were reconstructed via ancestral sequence reconstruction (ASR) are indicated. Branch supports values are shown for these nodes and additional important internal branches as Felsenstein's bootstrap values and approximate likelihood ratio test statistics (fbe/aLRT). Four sequences of characterized extant CS were not included in the inference of the main phylogeny used for ASR. Their position on the tree was inferred afterwards by recalculating the phylogeny and is indicated with dashed lines.

a

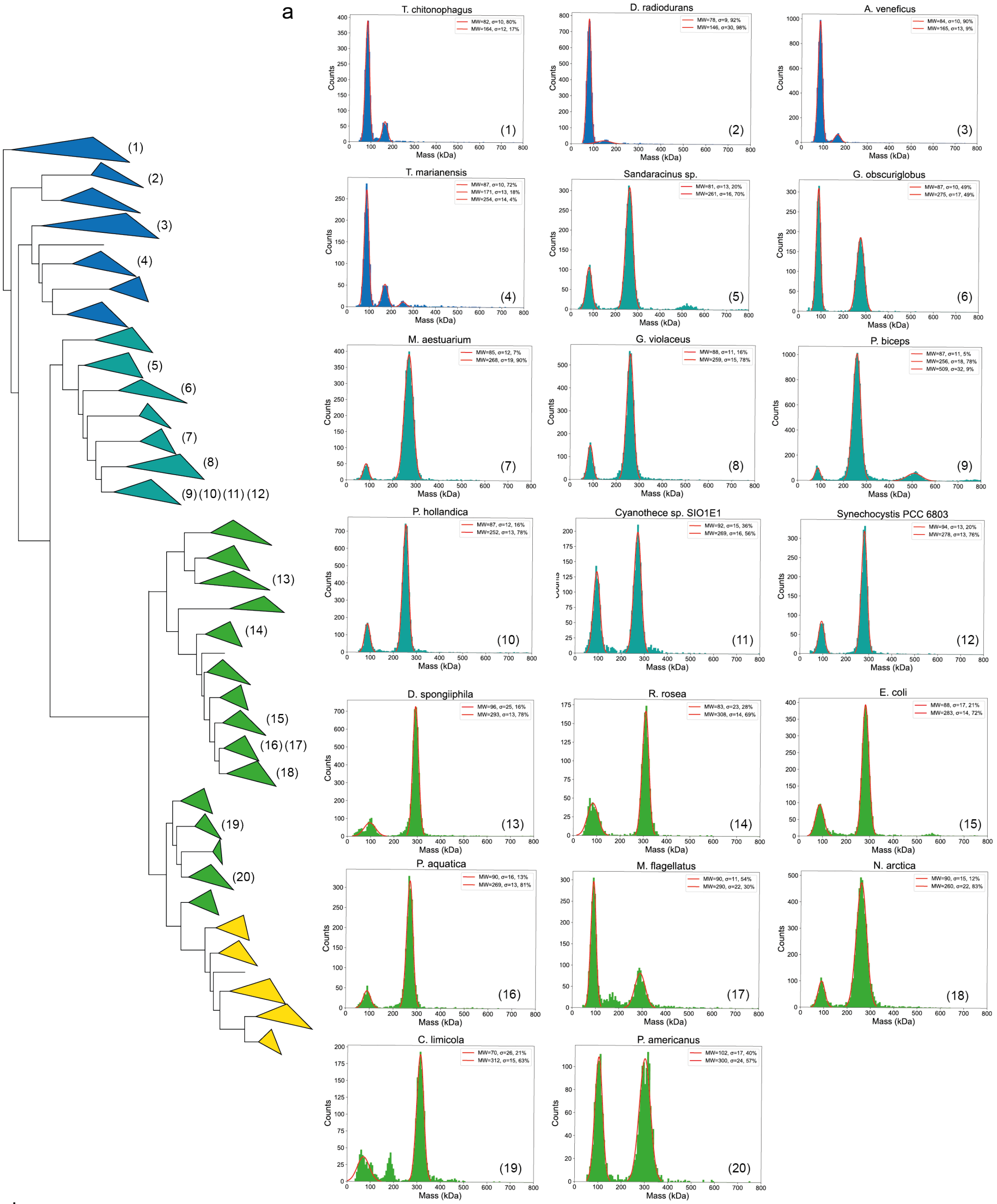

b Deposited CS structures

Type I dimer	2P2W, 2R26, 1O7X, 1VGP, 1IOM, 6ABY, 4YBO, 1AJ8, 2IBP, 1A59
Type II dimer	4TVM, 3MSU
Type II hexamer	4XGH, 4G6B, 6ZU0, 2H12, 7E8N, 4E6Y

Supplementary Fig. 2 Mass photometry measurements of additional characterized extant citrate synthase (a) Mass photometry histograms of additional purified extant citrate synthases

(CS) and their respective position on the inferred phylogenetic tree. (b) PDB accession codes of previously characterized and deposited structures of prokaryotic CS. Source data are provided as a Source Data file.

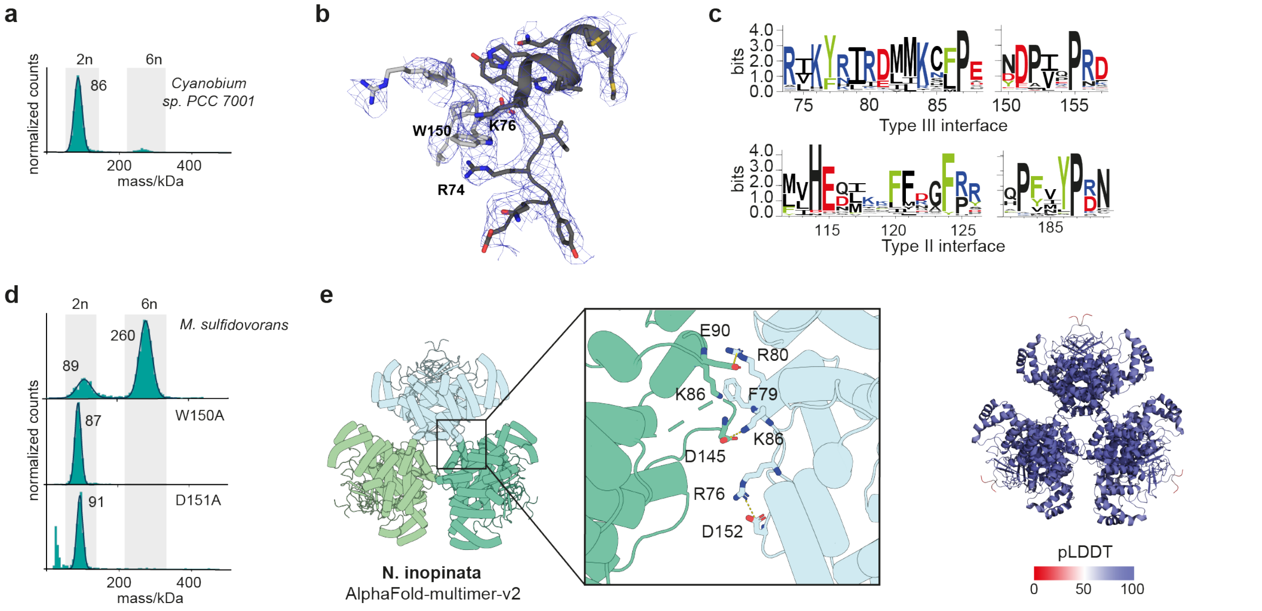

Supplementary Fig. 3 Important residues for type III hexamer formation (a) Mass photometry (MP) measurement of the dimeric type III citrate synthase (CS) from *Cyanobium* sp. PCC 7001. (b) Electron density map of the crystal structure of the CS from *M. sulfidovorans* at the interface of two subunits (compare Fig. 2d). The $2F_{obs}$-F_{calc} after final refinement is shown as a blue mesh at 1 σ. Protein is represented as cartoon with side chains as sticks. (c) Sequence logo of amino acid residues that are found within the interface of type II or III CS, demonstrating strong differences. Homologous sites are aligned, shift in site numbers results from a longer N-terminus in type II CS. (d) MP measurement of interface variants of the type III CS from *M. sulfidovorans*. (e) Structural prediction of the hexameric type III CS from *N. inopinata* inferred with AlphaFold-multimer-v2 with close up of the interface between dimers. Right: structural model colored according to the predicted local distance difference test (pLDDT). Source data are provided as a Source Data file.

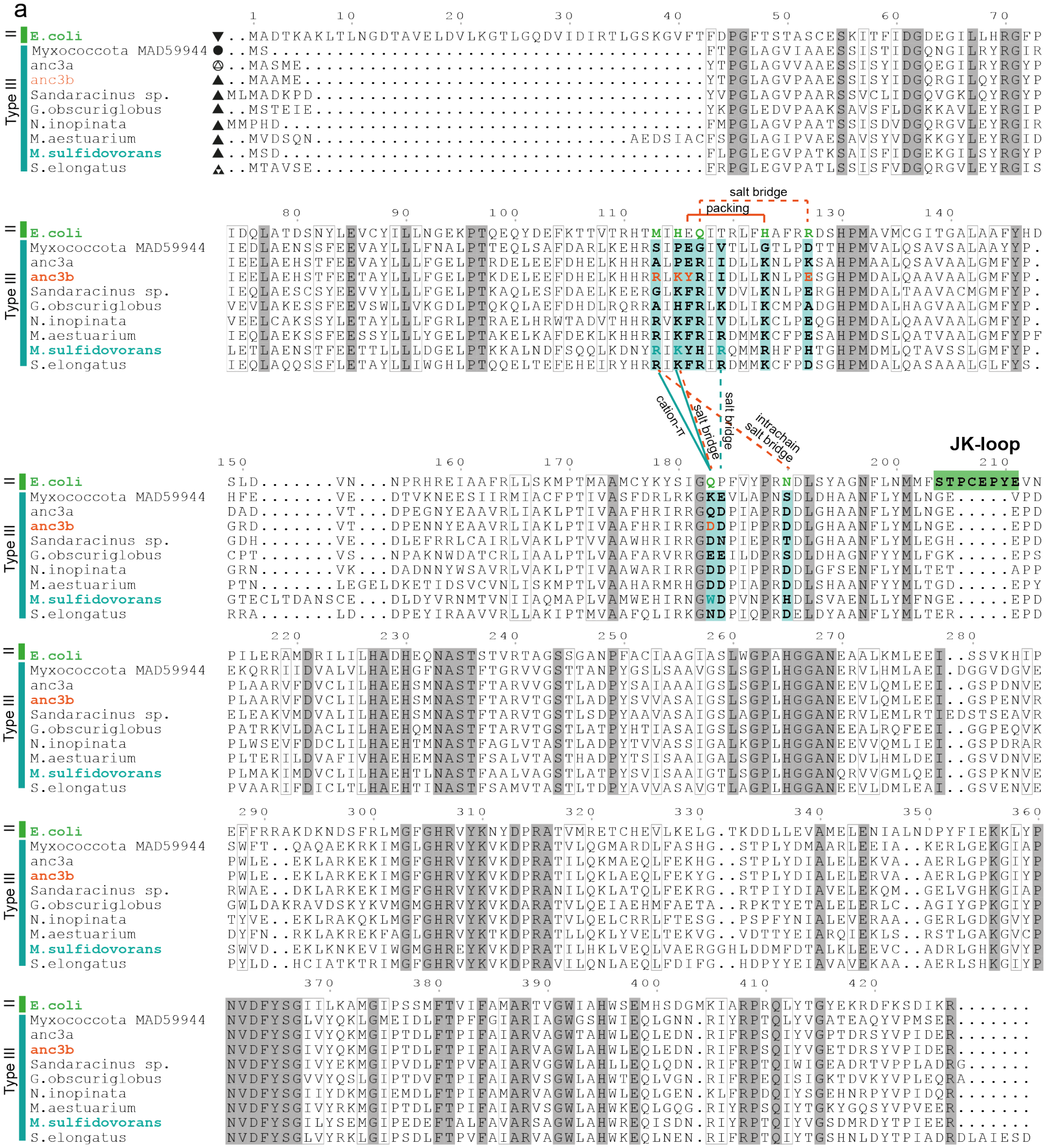

Supplementary Fig. 4 Conservation of residues inducing hexameric assembly in type III citrate synthase. Alignment of characterized type III citrate synthases (CS), ancestors anc$_{3a}$, anc$_{3b}$ and the type II CS from *E. coli*. Residues marked in teal are part of the hexamer interface in type III enzymes. Connecting lines indicate direct interactions between residues in structures (teal=*M. sulfidovorans*, orange=anc$_{3b}$). Residues that take part in the hexamer formation in *E. coli* CS are colored in green.

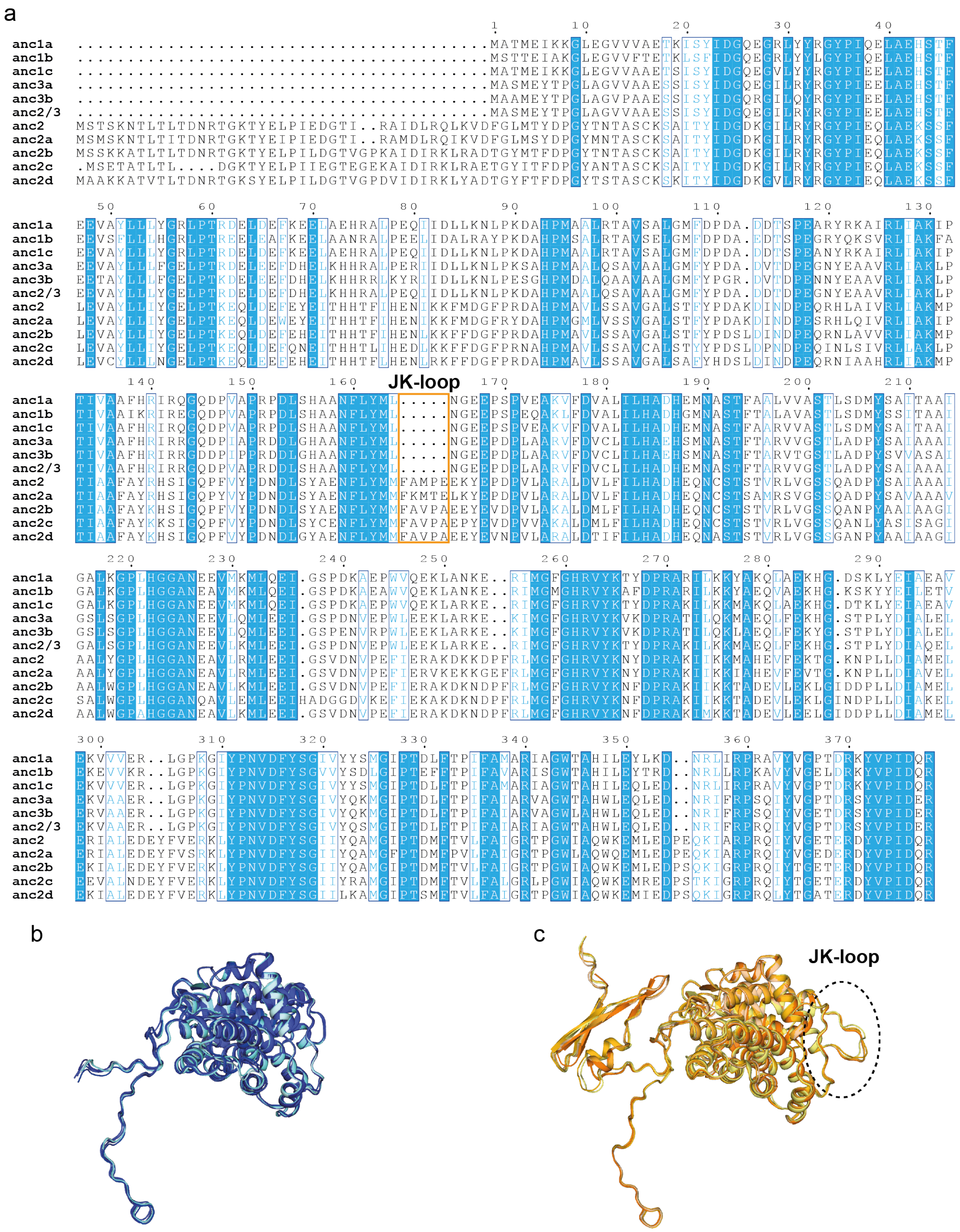

Supplementary Fig. 5 Sequences and predicted structures of reconstructed ancestral citrate synthase sequences (a) Alignment of the amino acid sequences of the inferred ancestral proteins. (b) Aligned Alphafold2 predictions of monomeric ancestral citrate synthase (CS) anc$_{1a}$,

anc_{1b}, anc_{1c}, $anc_{2/3}$, anc_{3a} and anc_{3b}. (c) Aligned Alphafold2 predictions of monomeric ancestral CS anc_2, anc_{2a}, anc_{2b}, anc_{2c} and anc_{2d}.

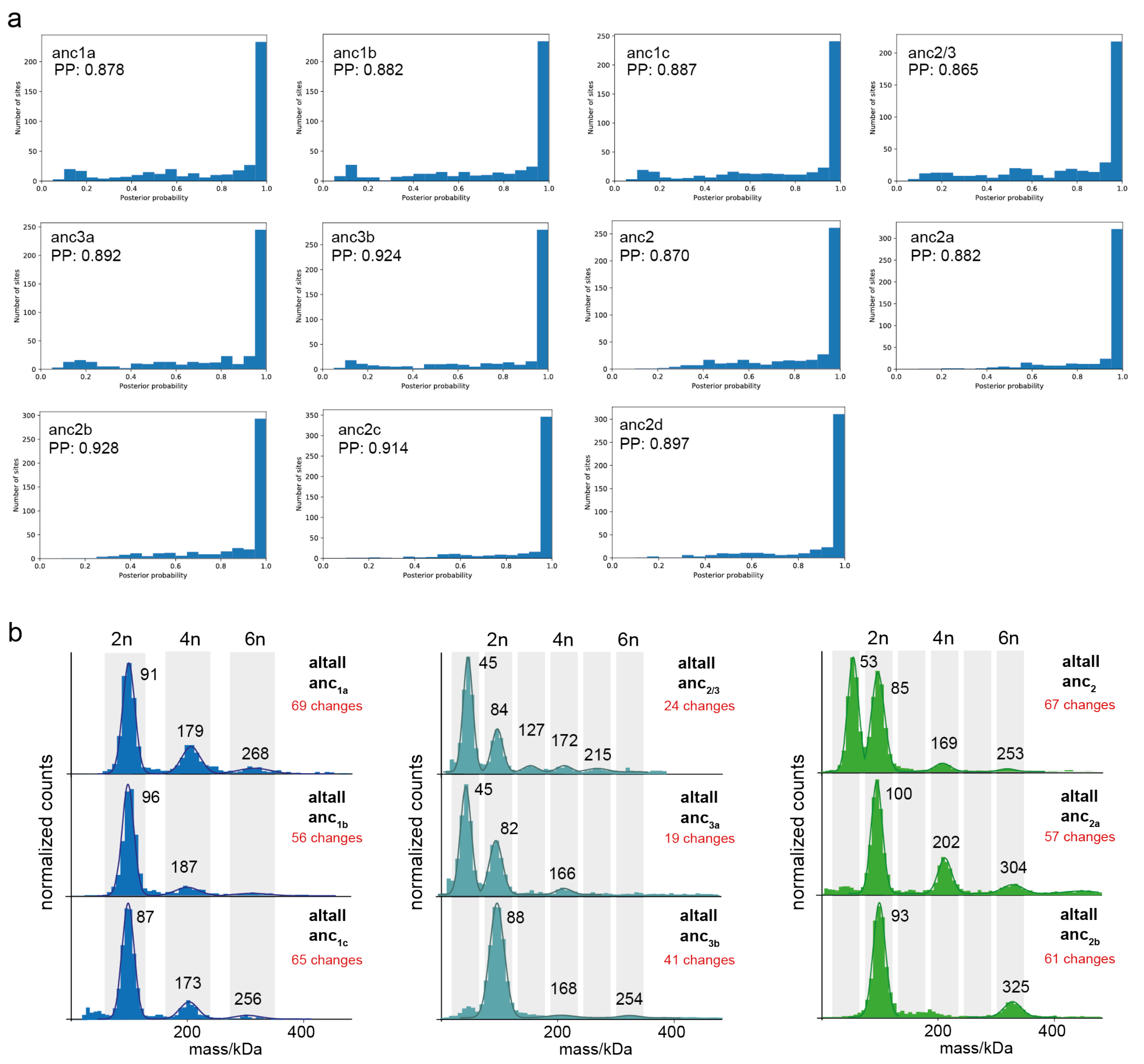

Supplementary Fig. 6 Robustness of the inferred ancestral proteins (a) Histograms per-site of the posterior probabilities (PP) of the maximum aposteriori state across reconstructed sites for all eleven ancestral proteins. (b) Mass photometry measurements of purified alternative ancestral proteins (altall = all position changed to the second most likely amino acid, if PP >0.2 and for altall $anc_{2/3}$ and anc_{3a} if PP >0.3). Number of changes denotes the differences to the maximum-likelihood ancestral sequence. Source data are provided as a Source Data file.

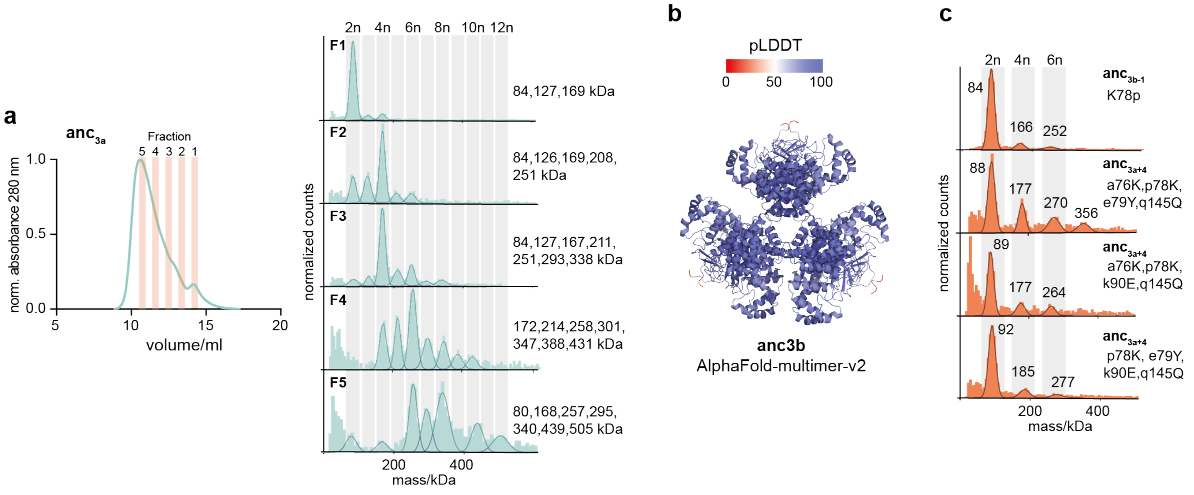

Supplementary Fig. 7 Type III ancestors assemble into polydisperse distributions (a) Size exclusion chromatography trace of the polydisperse anc_{3a} and mass photometry (MP) measurements of the isolated fractions. (b) MP measurements of variants of anc_{3a} with different combinations of substitutions that emerged in the interval to anc_{3b} (small and capital letters indicate the ancestral and derived amino acid, respectively). No combination of 4 was sufficient to induce hexameric assembly. The significance of the substitution p78K was shown by a reversal (K78p) in the hexameric anc_{3b}. (c) AlphaFold-multimer prediction of anc3b color-coded according to the site-wise pLDDT score. Source data are provided as a Source Data file.

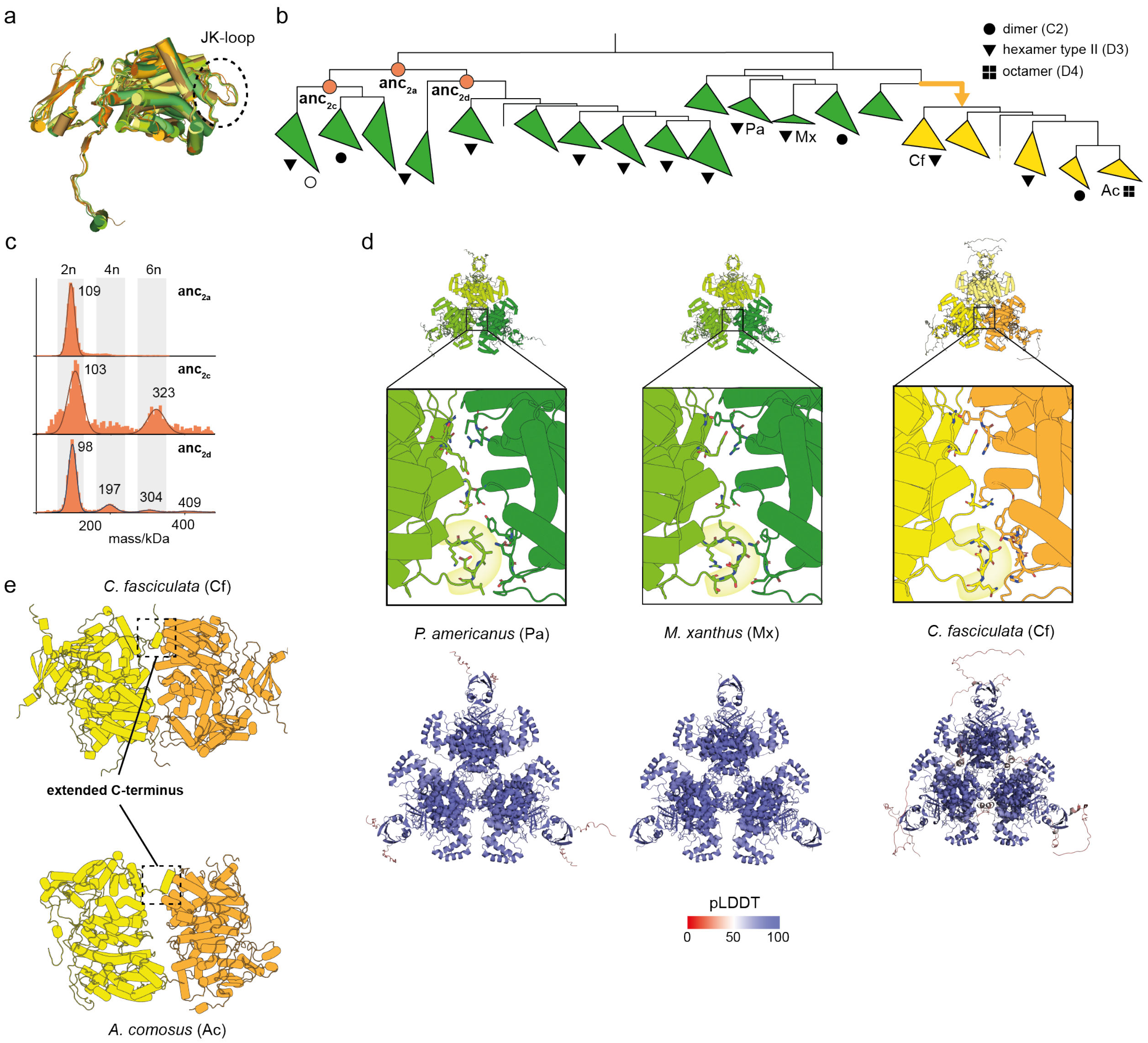

Supplementary Fig. 8 Inconclusive inference of the emergence of type II hexamers but conserved involvement of the JK-loop (a) Structural alignment of deposited type II citrate synthases (CS). All have the JK-loop (PDB: 4E6Y, 6ZU0, 2H12, 4G6B, 4XGH, 7E8N, 4TVM, 3MSU). (b) Schematic representation of part of the CS phylogeny displaying the quaternary structures of characterized type II CS. Nodes corresponding to resurrected ancestral CS are indicated (orange circles). (c) Mass photometry measurements of anc_{2a} and its descendants anc_{2c} and anc_{2d}. (d) Upper: AlphaFold-Multimer predictions of hexameric type II CS and non-mitochondrial CS with close ups on the interface that display the involvement of the JK-loop for all of them. Lower: pLDDT-colored structures of the same models. (e) Comparison of the extended C-terminus in the hexameric structure from *C. fasiculata* (AlphaFold-Multimer prediction) and octameric structure from *A. comosus* (cryo-EM). Only two dimers are displayed

for both structures to highlight the cross-connection by the C-terminus between adjacent dimers. Source data are provided as a Source Data file.

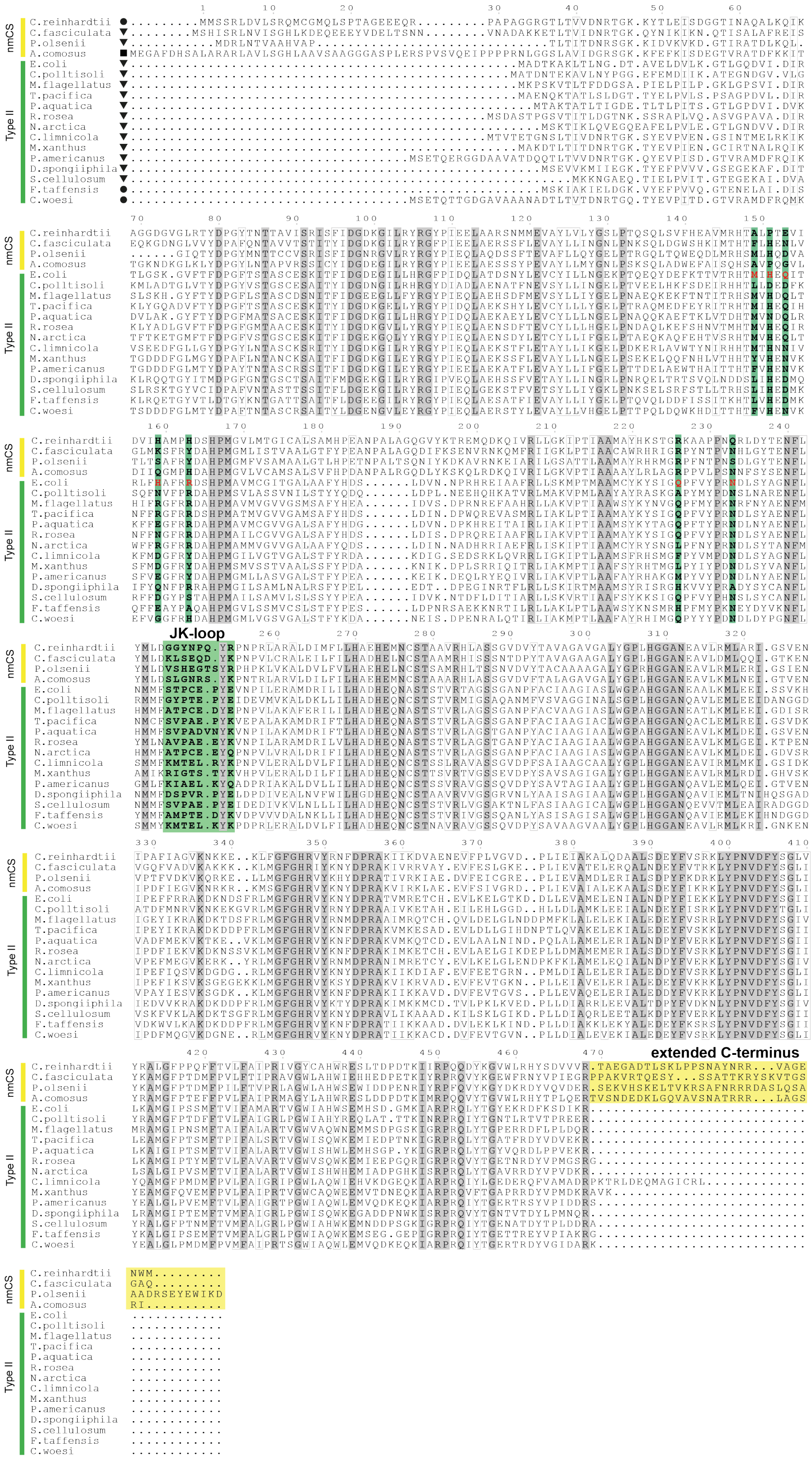
nmCS
Type II
C.reinhardtii
C.fasciculata
P.olsenii
A.comosus
E.coli
C.polltisoli
M.flagellatus
T.pacifica
P.aquatica
R.rosea
N.arctica
C.limnicola
M.xanthus
P.americanus
D.spongiiphila
S.cellulosum
F.taffensis
C.woesi
JK-loop
extended C-terminus

Supplementary Fig. 9 Conservation of residues inducing oligomeric assembly in type II citrate synthases (CS) and non-mitochondrial CS. Alignment of the characterized type II CS and nmCS. Residues marked in green take part in the hexamer formation in the hexamer from *E. coli*. The extended C-terminus of nmCS is colored in yellow.

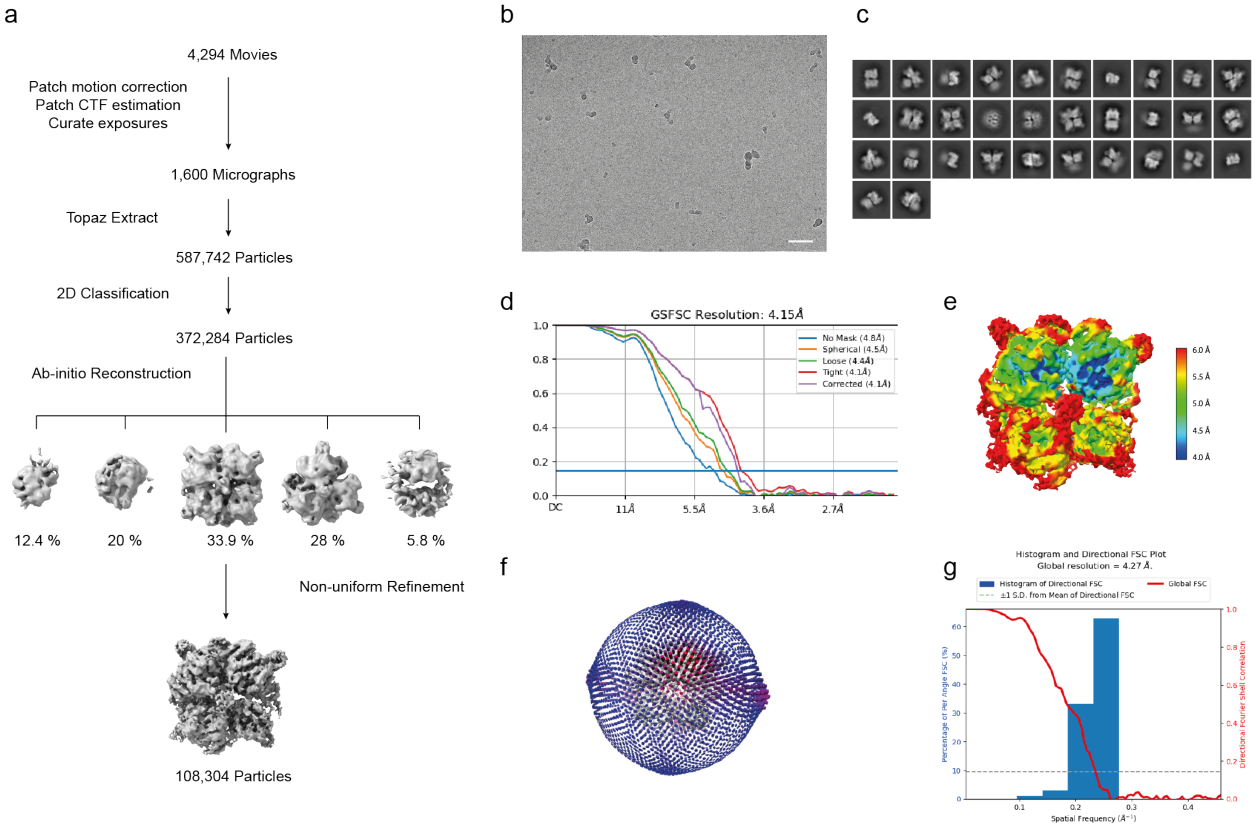

Supplementary Fig. 10 Cryo-EM data processing. (a) An overview of the image processing procedure (see Methods). (b) A representative micrograph of *A. cosmosus* CS acquired from FEI Titan Kriso (scale bar, 50 nm). (c) 2D classes selected for ab-initio reconstruction. (d) Fourier shell correlation (FSC) curves for the reconstruction applying different masks. (e) The final density map colored by local resolution. (f) Euler angular distribution of particles used in the final 3D reconstruction. (g) The histogram of directional FSC.

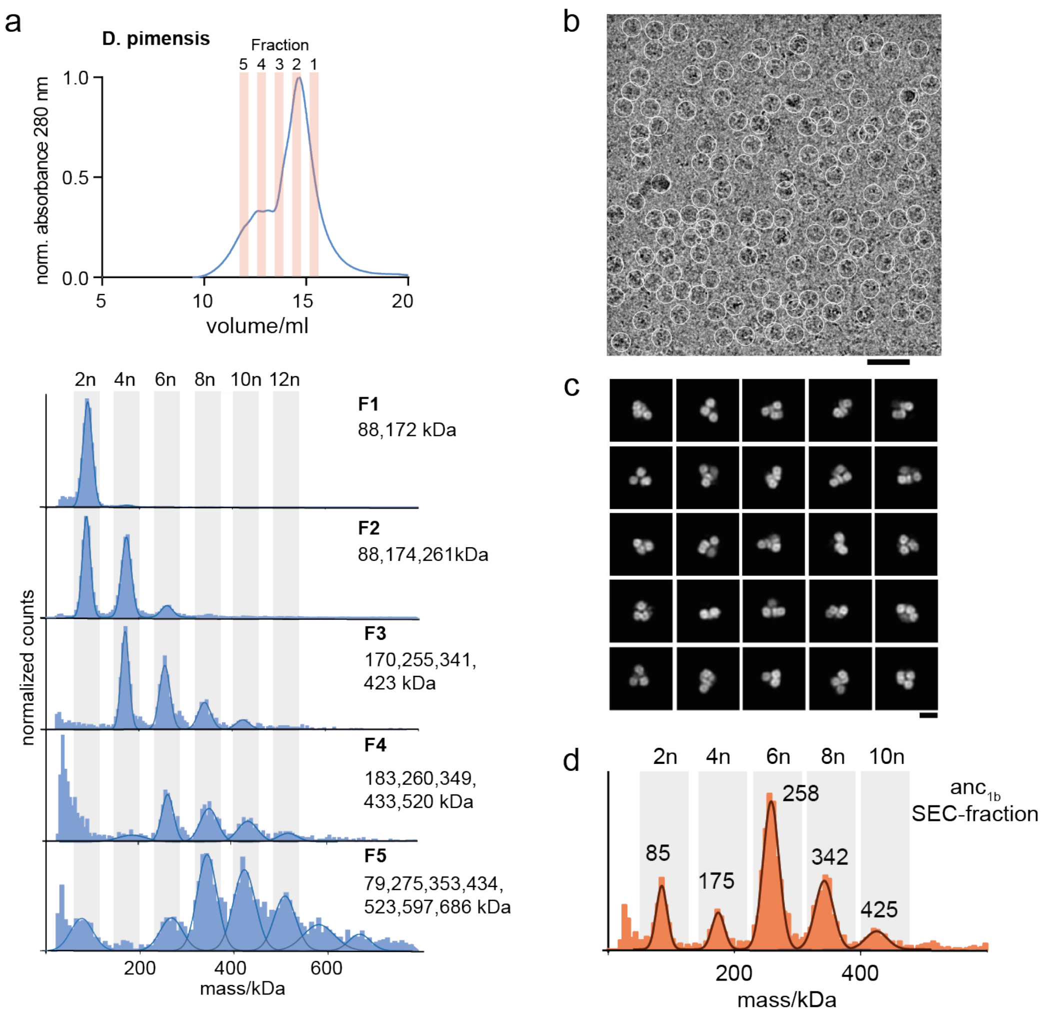

Supplementary Fig. 11 Polydisperse type I enzymes form multiples of dimers (a) Size exclusion chromatography (SEC) trace of polydisperse citrate synthase (CS) from *D. pimensis* and mass photometry (MP) measurements of the isolated fractions. (b) Exemplary cryo-EM micrograph of oligomeric complexes of polydisperse anc_{1a}, low-pass filtered at 5 Å. Particle picks are depicted as white circles (diameter = 180 Å). Scale bar = 40 nm. (c) 2D class averages of polydisperse anc_{1a}. Scale bar = 10 nm. (d) MP measurement of the SEC-fraction used for cryo-EM. Source data are provided as a Source Data file.

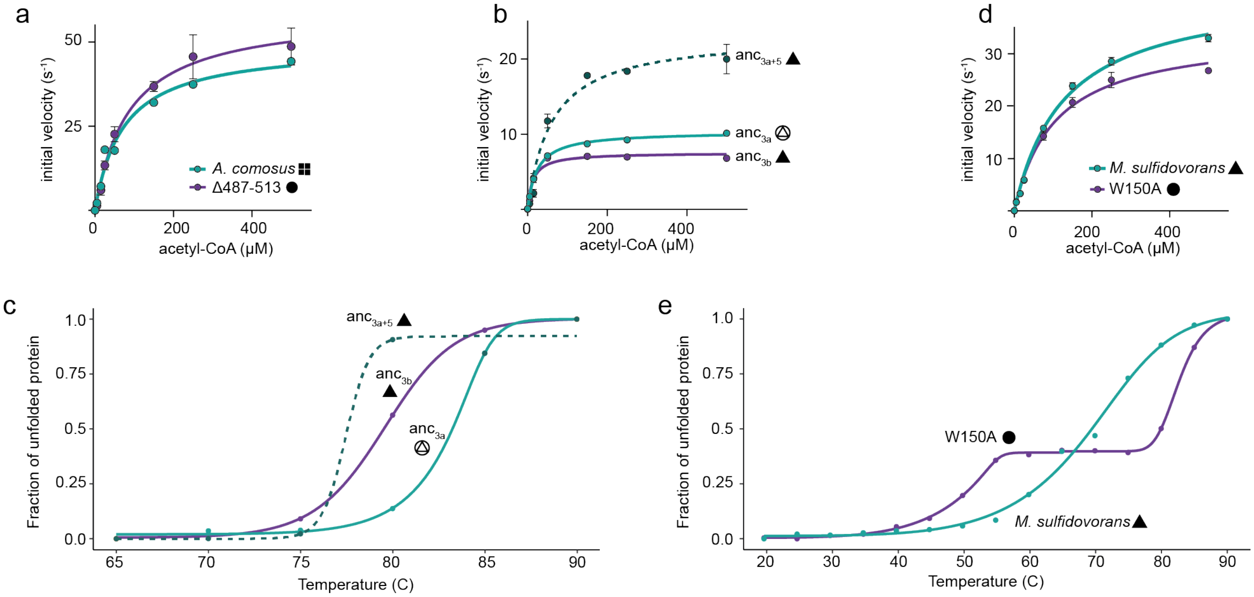

Supplementary Fig. 12 Kinetic measurements with saturated oxaloacetate and thermal stability (a) Michaelis-Menten kinetics of extant citrate synthase (CS) from *A. comosus* and a variant that disrupts interfaces between dimeric subcomplexes and prevent assembly into larger oligomers (Δ487-513). Data are presented as mean values +/- SD, n = 3 technical replicates. (b) Michaelis-Menten kinetics of the ancestral CS bracketing the emergence of hexamers within type III enzymes (anc$_{3a}$, anc$_{3b}$) and the minimal substitution construct to yield hexameric complexes (anc$_{3a+5}$). Data are presented as mean values +/- SD, n = 3 technical replicates. (c) Thermal stability measurements of the ancestral CS (anc$_{3a}$, anc$_{3b}$, anc$_{3a+5}$) using circular dichroism to evaluate folding of the protein. (d) Michaelis-Menten kinetics of extant CS from *M. sulfidovorans* and a variant that disrupts interfaces between dimeric subcomplexes and prevent assembly into larger oligomers (W150A). Data are presented as mean values +/- SD, n = 3 technical replicates. (e) Thermal stability measurements of the extant CS *M. sulfidovorans* and a variant that disrupts interfaces between dimeric subcomplexes and prevent assembly into larger oligomers (W150A) using circular dichroism to evaluate folding of the protein. Source data are provided as a Source Data file.

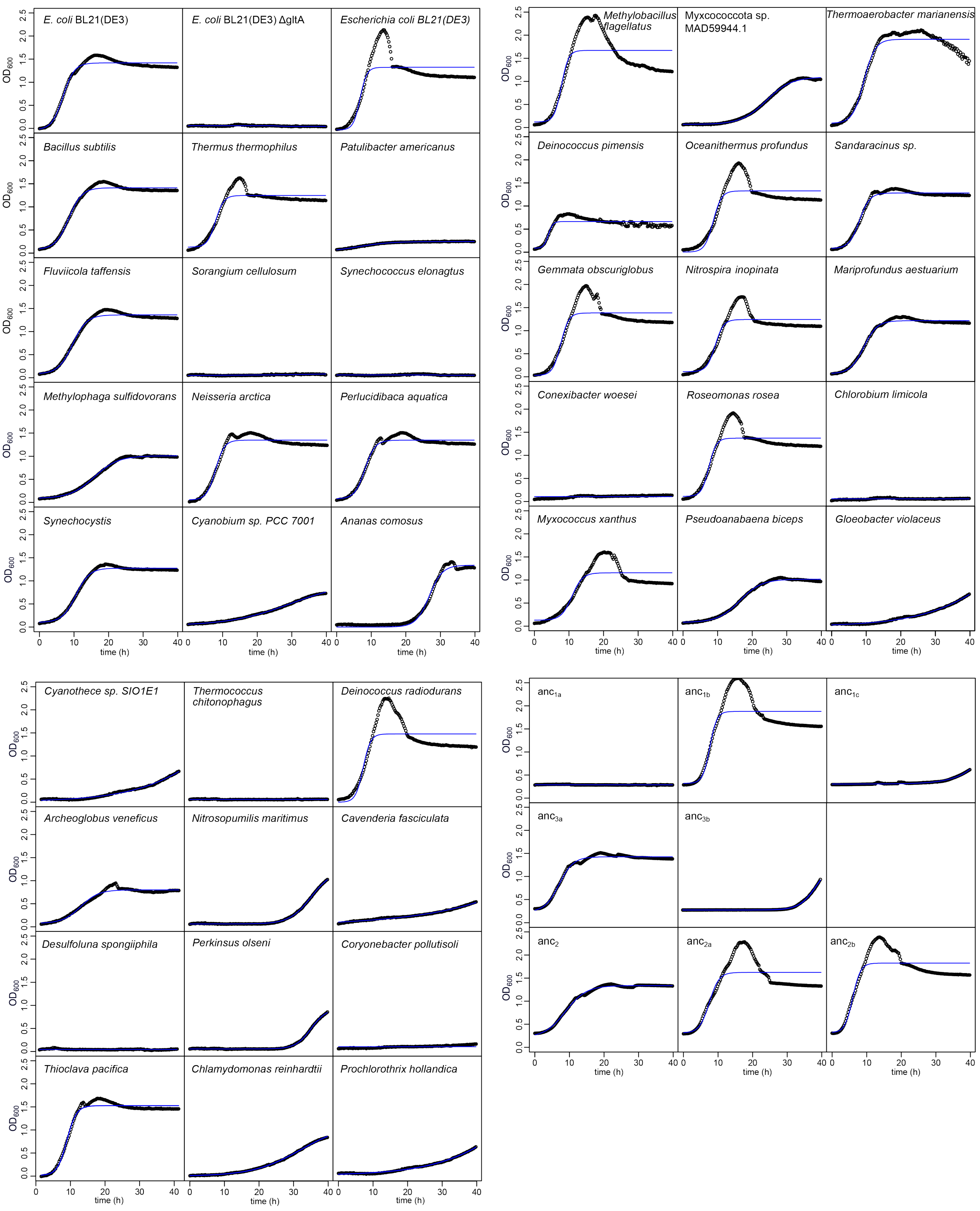

Supplementary Fig. 13 Growth data of complemented strains with fitted growth curves

Source data are provided as a Source Data file.

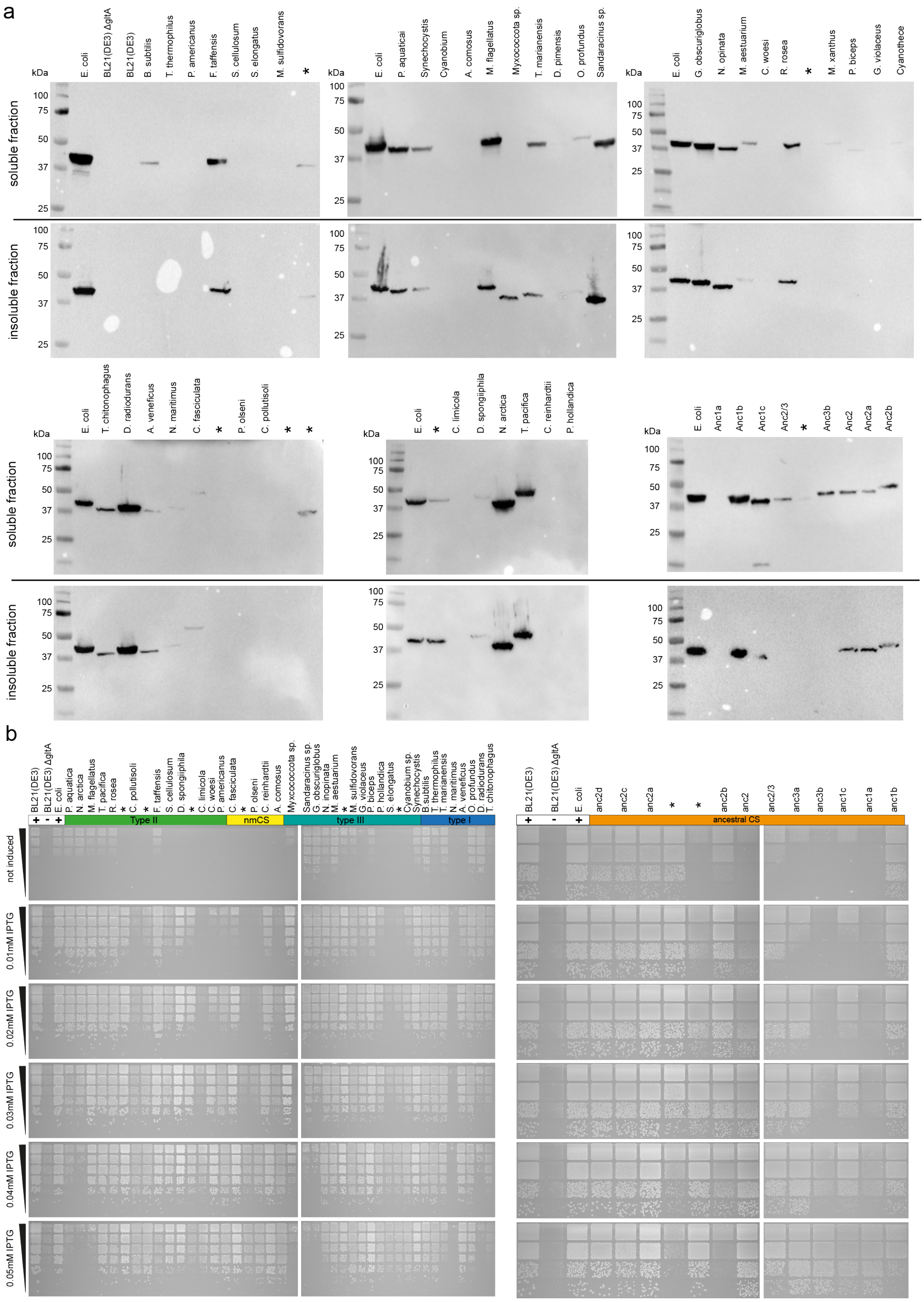
a
kDa
E. coli
BL21(DE3) ΔgltA
BL21(DE3)
B. subtilis
T. thermophilus
P. americanus
F. taffensis
S. cellulosum
S. elongatus
M. sulfidovorans
*
P. aquaticai
Synechocystis
Cyanobium
A. comosus
M. flagellatus
Myxococcota sp.
T. marianensis
D. pimensis
O. profundus
Sandaracinus sp.
G. obscuriglobus
N. opinata
M. aestuarium
C. woesi
R. rosea
M. xanthus
P. biceps
G. violaceus
Cyanothece
soluble fraction
insoluble fraction
100
75
50
37
25
T. chitonophagus
D. radiodurans
A. veneficus
N. maritimus
C. fasciculata
P. olseni
C. pollutisoli
C. limicola
D. spongiiphila
N. arctica
T. pacifica
C. reinhardtii
P. hollandica
Anc1a
Anc1b
Anc1c
Anc2/3
Anc3b
Anc2
Anc2a
Anc2b
b
BL21(DE3)
BL21(DE3) ΔgltA
E. coli
P. aquatica
M. flagellatus
R. rosea
C. pollutisoli
F. taffensis
S. cellulosum
C. woesi
P. americanus
Myxococcota sp.
Sandaracinus sp.
G. obscuriglobus
N. inopinata
M. aestuarium
M. sulfidovorans
G. violaceus
P. biceps
P. hollandica
S. elongatus
Cyanobium sp.
Synechocystis
B. subtilis
T. thermophilus
T. marianensis
N. maritimus
A. veneficus
O. profundus
D. radiodurans
T. chitonophagus
+
-
Type II
nmCS
type III
type I
ancestral CS
anc2d
anc2c
anc2a
anc2b
anc2
anc2/3
anc3a
anc3b
anc1c
anc1a
anc1b
not induced
0.01mM IPTG
0.02mM IPTG
0.03mM IPTG
0.04mM IPTG
0.05mM IPTG

Supplementary Fig. 14 Protein production and complementation on solid media (a) Western Blots depicting the citrate synthase (CS) protein production of all tested extant and ancestral CS enzymes in *E. coli*. Comparison of protein content in the soluble and insoluble fraction after cell lysis. (b) Spot-assays of complemented *E. coli* BL21(DE3) ΔgltA strains. Cultures were spotted in a five-step serial dilution using a ratio of 1:5 for each step and incubated on M9-solid media supplemented with glucose and different Isopropyl β- d-1-thiogalactopyranoside (IPTG) concentrations. One representative plate is shown for each experiment, out of a total of three replicates for each plate.

Supplementary Table 1 Data collection and refinement statistics for the crystal structure of *M. sulfidovorans* citrate synthase (CS)

	**CS from *M.sulfidovorans* PDB 8QWB**
**Wavelength** (Å)	0.9762
**Resolution range** (Å)	48.31 - 3.201 (3.316 - 3.201)
**Space group**	P 1 21 1
**Unit cell** (Å)	155.69 96.61 203.1 90 110.41 90
**Total reflections**	636593 (60352)
**Unique reflections**	92644 (9175)
**Multiplicity**	6.9 (6.6)
**Completeness (%)**	98.85 (97.95)
**Mean I/sigma(I)**	10.32 (0.88)
**Wilson B-factor**	114.97
R_{merge}	0.1319 (2.263)
R_{meas}	0.1428 (2.457)
R_{pim}	0.05402 (0.9447)
**CC1/2**	0.998 (0.467)
**CC***	1 (0.798)
**Reflections used in refinement**	92635 (9161)
**Reflections used for R-free**	4635 (459)
R_{work}	0.3252 (0.4478)
R_{free}	0.3245 (0.4490)
**CC (work)**	0.890 (0.463)
**CC (free)**	0.883 (0.481)
**Number of non-hydrogen atoms**	30067
**macromolecules**	30067
**Protein residues**	3816
**R.m.s. deviation**	
Bond lengths (Å)	0.012
Bond **angles** (°)	1.35
**Ramachandran favored (%)**	93.70
**Ramachandran allowed (%)**	6.22
**Ramachandran outliers (%)**	0.08
**Rotamer outliers (%)**	2.84
**Clashscore**	28.54
**Average B-factor** ($Å^2$)	134.04
M**acromolecules** ($Å^2$)	134.04

Statistics for the highest-resolution shell are shown in parentheses.

Supplementary Table 2 Cryo-EM data collection, refinement, and validation statistics of non-mitochondrial citrate synthase (CS) from *A. comosus*

	CS *A. comosus* **PDB 8QZP** **EMD-18779**
**Data collection and Processing**	
Microscope	Titan Krios
Voltage (keV)	300
Camera	Gatan K3
Magnification	29,000X
Pixel size at detector (Å/pixel)	1.09
Total electron exposure (e^-/Å^2)	55
Exposure rate (e^-/pixel/sec)	4.5
Number of frames collected during exposure	30
Defocus range (μm)	-3.0 to -0.5
Automation software	SerialEM
Micrographs collected (no.)	4,294
Micrographs used (no.)	1,600
Total extracted particles (no.)	587,742
**Reconstruction**	
Refined particles (no.)	372,284
Final particles (no.)	108,304
Symmetry imposed	C1
Resolution (global, Å)	
FSC 0.143 (unmasked/masked)	4.1/4.8
Resolution range (local, Å)	53.9 to 2.3
Map sharpening *B* factor (Å^2)	-222.5
Map sharpening methods	Global B factor
**Model composition**	
Protein	CS A. comosus
**Model Refinement**	
Real space refinement software	PHENIX v1.19.2
Model-Map scores (CC_mask)	0.75
B factors (Å^2)	(min/max/mean)
Protein	30.00/329.44/169.74
R.m.s. deviations from ideal values	
Bond lengths (Å)	0.005
Bond angles (°)	0.797
**Validation**	
MolProbity score	2.41
CaBLAM outliers	3.76
Clashscore	22.20
Poor rotamers (%)	1.23
Cβ outliers (%)	0.00
EMRinger score	0.30
**Ramachandran plot**	
Favored (%)	91.49
Allowed (%)	7.98
Outliers (%)	0.52

Supplementary Table S3 Kinetic enzyme parameters of *D. pimensis* citrate synthase and isolated size exclusion chromatography fractions containing different oligomeric state distributions

	k_{cat} (s^{-1})	$K_m$oxaloacetate (µM)
***D. pimensis* – non-fractionated**	36.6 ± 1.1	25.4 ± 3.1
**F1**	39.9 ± 1.3	29.6 ± 3.9
**F2**	23.2 ± 0.6	15.0 ± 1.8
**F3**	16.6 ± 0.5	17.7 ± 2.5
**F4**	18.4 ± 0.6	18.4 ± 2.5
**F5**	14.2 ± 0.7	25.9 ± 5.0

Measurements were performed at 25 °C. N=3 technical replicates, errors = SEM

Supplementary Table 4 Kinetic characterization for different extant and ancestral citrate synthases and their variants

	**aceytlCoA**		**oxaloacetate**	
	k_{cat} (s^{-1})	$K_m$acetyl-CoA (μM)	k_{cat} (s^{-1})	$K_m$oxaloacetate (μM)
**anc$_{3a}$**	9.1 ± 0.3	22.4 ± 2.6	10.3 ± 0.1	23.9 ± 1.1
**anc$_{3b}$**	5.6 ± 0.2	8.6 ± 1.9	7.5 ± 0.2	13.2 ± 2.2
**anc$_{3a+5}$**	21.9 ± 0.8	56.5 ± 6.8	23.3 ± 61.1	61.1 ± 10.2
M. sulfidovorans WT	42.4 ± 1.0	127.4 ± 7.7	33.3 ± 1.8	50.5 ± 5.2
M. sulfidovorans W150A	34.1 ± 1.0	104.7 ± 8.9	26.7 ± 1.1	36.7 ± 5.7
A. comosus WT	41.1 ± 1.5	13.2 ± 2.3	49.2 ± 2.2	72.1 ± 10.1
A. comosus Δ487-513	41.8 ± 1.5	11.3 ± 2.0	58.9 ± 2.6	87.2 ± 11.7

Measurements were performed at 25 °C. N=3 technical replicates, errors = SEM
