# Supplementary material for: Frequent transitions in self-assembly across the evolution of a central metabolic enzyme
Source: Nat Commun. 2024 Dec 3;15:10515. doi: 10.1038/s41467-024-54408-6 (PMC11615384; doi:10.1038/s41467-024-54408-6)
Supplement: Supplementary file 2 — Description of Additional Supplementary Files [file 41467_2024_54408_MOESM2_ESM.pdf]

Description of Additional Supplementary Files

File name: Supplementary Data 1

Description: DNA sequences used in this study
