# Supplementary material for: Frequent transitions in self-assembly across the evolution of a central metabolic enzyme
Source: Nat Commun. 2024 Dec 3;15:10515. doi: 10.1038/s41467-024-54408-6 (PMC11615384; doi:10.1038/s41467-024-54408-6)
Supplement: Supplementary file 3 — Supplementary Data 1 [file 41467_2024_54408_MOESM3_ESM.docx]

**Supplementary Data File. List of DNA sequences of proteins used in this study**

| Protein  (Accession number) | DNA sequence |
| --- | --- |
| Anc 1a | ATG GCC ACG ATG GAG ATC AAA AAG GGA CTG GAG GGA GTG GTG GTT GCC GAA ACG AAA ATC TCT TAT ATT GAT GGC CAG GAA GGA CGT TTG TAT TAC CGT GGG TAT CCT ATC CAA GAA TTA GCT GAG CAT TCT ACC TTC GAG GAG GTC GCG TAT TTA CTT CTT TAT GGA CGC CTG CCA ACG CGT GAT GAA CTG GAC GAG TTC AAA GAA GAG CTT GCA GAA CAT CGT GCT CTG CCA GAA CAA ATT ATT GAT TTG TTA AAA AAC CTT CCA AAG GAT GCC CAC CCA ATG GCC GCT CTT CGC ACG GCA GTC TCC GCT CTG GGA ATG TTC GAT CCG GAT GCA GAC GAT ACC TCA CCT GAA GCT CGC TAC CGC AAG GCC ATC CGC TTG ATC GCA AAA ATT CCG ACG ATC GTA GCT GCC TTT CAC CGT ATT CGC CAA GGA CAG GAC CCA GTA GCA CCG CGT CCT GAC TTG AGT CAC GCT GCC AAC TTC CTG TAT ATG CTG AAT GGC GAA GAA CCT TCG CCG GTG GAG GCC AAA GTG TTT GAT GTG GCT CTT ATC CTT CAT GCG GAC CAT GAG ATG AAC GCT TCC ACC TTC GCC GCA TTA GTC GTC GCG AGT ACT TTA TCA GAC ATG TAC AGT GCC ATT ACG GCT GCC ATT GGG GCC TTG AAG GGG CCG CTT CAT GGG GGT GCC AAC GAA GAG GTG ATG AAG ATG TTA CAA GAA ATT GGC TCA CCT GAT AAA GCT GAA CCC TGG GTG CAG GAG AAA CTT GCG AAT AAG GAA CGC ATT ATG GGC TTT GGA CAC CGT GTT TAC AAG ACT TAT GAT CCC CGT GCC CGC ATT TTG AAA AAA TAC GCG AAG CAG CTT GCC GAG AAA CAC GGT GAT TCC AAG CTG TAC GAG ATT GCT GAG GCG GTT GAG AAA GTG GTC GTG GAG CGC TTG GGG CCG AAA GGT ATT TAT CCA AAT GTG GAC TTT TAT TCA GGG ATT GTC TAT TAC TCC ATG GGT ATC CCA ACA GAC TTG TTT ACA CCT ATC TTC GCA ATG GCC CGT ATT GCT GGA TGG ACA GCT CAT ATT CTT GAA TAT CTT AAA GAT AAT CGC CTG ATC CGC CCT CGT GCA GTG TAT GTC GGT CCC ACG GAT CGC AAG TAC GTA CCC ATT GAC CAA CGT |
| Altall Anc 1a | ATGAGCAGTATGGAGGTGGCTAAGGGTCTTGAAGGAGTTGTAGCAACAGAGACTTCCATTTCGTATATCGATGGGGAGGAGGGAGTACTGTATTACCGTGGCTATCCCATTGAAGAATTAGCAGAGCATAGCTCGTTTGAAGAAGTTGCTTATCTTCTTCTTCACGGAAAACTGCCTACCCGCGAAGAACTGGAGGATTTCAAAGAGGAGTTGGCAGAAAATCGTGCCATTCCAGAAGAGGTTGTCGATATGCTGCGCAGCCTTCCTAAAAATGCGCACCCCATGTCAGCTCTGCGTACAGCCGTAAGTGCTTTAGGAATGTTTGATCCTGAGGCTGACGACACTAGTCCGGAAGGCAACTATCAAAAGGCCGTGCGCTTGATTGCAAAAATTCCCACAATTGTGGCAGCGTTTCATCGCATCCGTAAGGGGCAAGACCCTGTTGAACCTCGCCCTGATTTAAGTCACGCGGCCAATTTTCTGTATATGCTTCACGGAGAGGAGCCTAGCCCAGTCCAAGAGCGCGTTATGGACGTTGCACTGATTCTGCATGCTGAACACGAAATGAACGCATCTACATTTACGGCTTTAGTGGTGGCGTCTACTCTTTCGGACATGTATTCAGCGATTACAGCCGCTATTGGCGCACTTAAAGGGCCCCTTCATGGGGGGGCCAATGAGCAAGTTATGAAAATGCTTCAGGAGATTGGATCCCCAGATAAGGTTGAAGCATGGATTAAGGACAAATTGGAGCGCAAAGAACGCATTATGGGGTTCGGCCACCGTGTGTACAAGACCTATGACCCTCGTGCGAAAATCCTTAAAAAATATGCGCGCCAGCTTGCGGAAAAGAAGGGCGACACTAAGTGGTACGAGATTGCAGAAAAGATTGAGAAGGTCGTAGTTAAACGTTTGGGGGAAAAAGGAATCTATCCTAATGTCGATTTCTATTCAGGCGTCGTTTACCACTCTCTTGGTATCCCGACTGAGCTGTTCACGCCAATTTTTGCTATCGCGCGTATCAGTGGCTGGACGGCGCATGTCTTAGAGTATCTTGAGGATAACCGCCTTATCCGTCCGCGTGCCATTTATGTCGGACCGACAGATCGTAAGTATATTCCGCTTGACGAGCGT |
| Anc 1b | ATG TCG ACT ACA GAA ATC GCT AAG GGC CTG GAG GGA GTG GTG TTC ACT GAA ACC AAA CTT TCC TTC ATC GAT GGG CAA GAG GGG CGT TTA TAC TAC CTT GGG TAT CCG ATC CAA GAG TTA GCG GAA CAT TCG ACT TTT GAG GAG GTA AGC TTC CTT TTA CTG CAC GGT CGT TTA CCG ACT CGC GAA GAG TTG GAG GCT TTC AAA GAA GAG CTT GCC GCC AAT CGT GCA TTA CCT GAG GAG CTT ATC GAC GCC TTG CGT GCC TAC CCC AAG GAC GCC CAC CCG ATG TCC GCC TTA CGT ACT GCC GTC AGT GAA TTA GGC ATG TTC GAT CCG GAT GCG GAG GAT ACA TCG CCG GAG GGG CGT TAC CAA AAA TCA GTG CGC CTT ATT GCA AAG TTT GCG ACA ATT GTG GCC GCC ATC AAA CGC ATT CGT GAG GGG CAA GAT CCA GTG GCG CCA CGT CCC GAT TTG TCC CAC GCG GCG AAC TTC TTA TAT ATG TTA AAT GGG GAA GAG CCG AGT CCC GAG CAG GCC AAG CTG TTT GAC GTA GCA TTG ATC CTG CAC GCT GAC CAC GGT ATG AAT GCG TCG ACG TTT ACC GCG TTA GCG GTA GCA TCG ACC CTT AGT GAC ATG TAT TCC TCC ATC ACC GCA GCC ATC GGC GCA CTT AAA GGT CCT TTA CAC GGT GGA GCA AAT GAA GCG GTT ATG AAG ATG TTG CAG GAA ATC GGG AGT CCG GAC AAA GCA GAA GCG TGG GTC CAA GAG AAA TTG GCG AAC AAG GAG CGC ATT ATG GGT ATG GGG CAC CGC GTC TAT AAA GCT TTC GAT CCT CGT GCT CGC ATC TTG AAA AAG TAC GCG GAA CAA GTG GCT GAA AAG CAT GGC AAG TCC AAG TAT TAC GAA ATT CTG GAG ACC GTA GAA AAG GAG GTC GTT AAA CGT CTT GGG CCT AAG GGA ATT TAC CCA AAC GTG GAC TTT TAC AGC GGG GTG GTG TAC TCA GAT CTT GGT ATC CCC ACA GAG TTT TTT ACG CCT ATT TTC GCA GTC GCA CGT ATC TCT GGT TGG ACG GCG CAT ATT CTG GAG TAT ACT CGC GAC AAT CGT CTG CTT CGT CCA AAG GCA GTG TAC GTG GGT GAA CTT GAC CGC AAG TAT GTC CCT ATT GAT CAA CGT |
| Altall Anc 1b | ATGGCTACGATGGAGATCGCTCGTGGACTGGAGGGGGTCGTTTTTACTGAGACTAAGTTGAGCTACATTGATGGTCAGGAAGGAGTTTTATATTACTTGGGATATCCCATTCAGGAGCTTGCAGAACACTCTAGCTTTGAGGAGGTGTCGTATTTATTGCTTCATGGGCGTCTGCCGACGAAAGATGAACTGGACGCTTTTAAGGAAGAACTGGCCGCTAATCGTGCTATCCCTGAAGAGATTGTTGAATCGTTAAAATCGTACCCTCGCAACGCCCATCCTATGGCCGCTTTGCGCACCGCTGTAAGTCAACTGGGAATGTTCGACCCGGATGCGGACGATACGAGCCCCGAAGGACGTTACCGTAAGTCGATTCGTTTAATTGCAAAGTTTTCGACGATTGTAGCGGCTTTGAAGCGTTTACGTGAGGGCCAGGAACCAGTAGAGCCTCGCCCAGACTTGTCACATGCCGCCAATTTCTTATATATGTTAAACGGAAAGGAGCCGTCGCCGGAACAGGAACGCCTGTTTGATGTGGCGCTTATTTTGCATGCGGAACATGGAATGAATGCATCAACCTTTACGGCCTTAGCCGTGGCCAGCACCCTTTCTGATATGTATTCCGCCATTACCGCGGCCATCGGAGCTTTGAAGGGTCCATTACATGGGGGTGCAAATGAAGCTGTCATGAAGATGTTACAAGAGATCGGAACACCGGAGCGCGCCGAACCTTGGATCCAGGATAAGTTAGAGAAAAAGGAACGCATCATGGGTATGGGTCATCGTGTGTACAAAACGTTCGACCCACGCGCCCGTATCTTGAAGAAGTATGCCGAGCACGTCGCAGAAAAACAGGGAAAGTCCAAGTATTATGAAATCCTTGAGACGATTGAGGAAGTGGTCGTTGAGCGTCTTGGACCAAAAGGAATTTACCCCAATGTGGACTTTTATTCTGGTGTAGTTTACTCCGACTTGGGCATTCCCACCGAATTCTTTACGCCAATTTTTGCTATCGCCCGTATTTCTGGTTGGACCGCACACGTTTTAGAATATACTCGCGATAATCGTTTGCTGCGCCCCCGCGCAGTATATGTCGGTCCTCTTGACCGCAAGTACGTTCCCTTGGAGGAGCGC |
| Anc 1c | ATG GCC ACT ATG GAG ATT AAG AAG GGG TTA GAA GGC GTG GTA GCG GCT GAG ACA TCG ATC TCT TAC ATC GAT GGT CAG GAG GGG GTC CTT TAC TAT CGT GGG TAT CCA ATC GAA GAG TTA GCA GAA CAC TCA ACA TTT GAG GAG GTG GCT TAT TTA TTA CTT TAC GGG CGT TTG CCC ACT CGT GAC GAA CTT GAC GAG TTT AAA GAG GAG CTG GCC GAA CAC CGT GCC TTA CCT GAA CAG ATC ATC GAC TTG CTT AAA AAT CTT CCC AAG GAC GCC CAT CCG ATG GCT GCG TTG CGT ACC GCT GTG TCA GCA CTT GGA ATG TTC GAC CCT GAC GCC GAT GAC ACC TCT CCG GAA GCG AAC TAC CGT AAA GCG ATC CGT CTT ATC GCA AAA ATT CCA ACG ATT GTG GCC GCC TTT CAC CGC ATT CGC CAA GGT CAA GAT CCC GTT GCG CCG CGT CCA GAC TTA TCA CAT GCT GCC AAT TTT CTT TAT ATG CTT AAC GGG GAG GAA CCC AGT CCT GTG GAG GCT AAA GTT TTC GAC GTG GCC CTT ATC CTT CAC GCA GAC CAT GAA ATG AAT GCA AGC ACG TTC GCC GCA CGC GTA GTG GCG TCG ACT TTA AGT GAC ATG TAT AGT GCG ATT ACG GCT GCC ATT GGT GCC TTG AAA GGT CCA CTT CAC GGA GGC GCG AAC GAG GAG GTC ATG AAA ATG TTG CAA GAA ATT GGC TCG CCA GAT AAA GCA GAG CCA TGG GTC CAA GAA AAA CTG GCG CGT AAG GAA CGC ATT ATG GGT TTT GGT CAC CGC GTA TAC AAG ACT TAT GAT CCG CGC GCG AAA ATC CTG AAA AAA ATG GCG AAG CAG TTG GCA GAA AAG CAT GGC GAT ACG AAG CTT TAC GAG ATC GCC GAA GCG GTC GAA AAG GTC GTT GTT GAG CGC CTT GGG CCG AAA GGC ATC TAT CCC AAC GTG GAC TTT TAC TCG GGT ATC GTT TAC TAC TCA ATG GGT ATT CCT ACA GAT TTG TTC ACT CCT ATT TTT GCA ATG GCT CGC ATC GCT GGC TGG ACG GCT CAT ATC TTG GAA CAG TTA GAA GAC AAC CGC CTT ATT CGT CCT CGT GCT GTT TAT GTG GGA CCT ACA GAC CGC AAA TAT GTC CCC ATC GAT CAA CGT |
| Altall Anc 1c | ATGTCATCTATGGAAGTCGCAAAAGGTTTAGAGGGAGTAGTTGCAGCAGAAAGTAAGATTAGTTACATCGACGGTGAGGAGGGCATTCTTACGTATCGTGGCTATCCAATCCAGGACCTTGCAGAACACAGTAGTTTTGAGGAAGTCGCGTATTTGTTATTACACGGTAAGCTTCCTACGCGCGAGGAGTTGGAAGACTTCAAGGAAGAGTTGGCGGAGCACCGCGCCATTCCGGAAGAAGTATTGGATATGTTGCGTAATTTGCCTAAGAACGCTCACCCGATGGCGGCCCTGCGTACCGCCGTATCCGCTTTGGGGATGTTCGACCCTGAAGCAGACGATACTAGTCCGGAGGGGAACTACCAGAAAGCCGTACGTTTAATCGCTAAAATCCCGACTATTGTAGCTGCATTCCACCGTATTCGTAAGGGCCAGGACCCGGTCGCACCGCGCCCAGATTTAAGCCATGCAGCAAACTTCTTATATATGTTGCATGGTGAGGAGCCTGACCCGATCCAAGCTCGCGTGTTTGATGTAGCGTTGATCCTGCACGCGGAACACGAAATGAATGCCAGCACGTTCGCCGCTCGTGTTGTAGCGAGTACGCTGTCCGATATGTACTCGGCGATCACTGCCGCAATCGGTGCACTGAAAGGTCCGTTACACGGGGGAGCCAATGAACAGGTCATGAAAATGTTACAAGAGATTGGAAGCCCAGACAAGGTCGAAGCGTGGATTAAGGATAAGCTTGAGAAGAAGGAGCGTATTATGGGATTTGGCCATCGTGTCTACAAGACTTACGACCCTCGCGCTCGTATCCTTCGCAAGATGGCACGTCAGTTAGCAGAAAAGAAAGGTGATAGTAAATGGTACGAAATCGCCGAAAAGATTGAACGCGTCGTTACAAAACGCCTGGGGGAAAAGGGTATTTACCCCAATGTGGACTTCTATAGCGGTGTGGTGTATCACTCCTTAGGAATTCCGACAGACTTGTTTACGCCGATTTTCGCAATTGCACGTATTTCAGGCTGGACTGCTCATGTTTTAGAACAACTTGAGAACAACCGCCTGATTCGTCCTCGCGCGATTTACGTTGGGCCAACAGATCGCAAATATGTCCCACTGGACGAGCGT |
| Anc 2/3 | ATGGCATCTATGGAGTATACCCCTGGGTTGGCAGGAGTGGTGGCGGCTGAATCCTCAATTTCTTATATTGATGGACAGGAGGGTATTTTGCGTTATCGCGGGTATCCAATCGAAGAGCTTGCGGAGCACTCAACCTTCGAAGAGGTAGCATACCTGTTGCTGTACGGCGAATTACCCACGCGCGACGAGTTAGATGAATTTGATCACGAACTGAAGCATCATCGCGCGTTACCAGAACAAATCATTGACCTTTTGAAAAATCTGCCGAAAGATGCCCATCCTATGGCCGCATTGCAGTCCGCCGTGGCGGCCTTAGGGATGTTTTATCCAGACGCAGACGATACGGATCCGGAGGGAAATTATGAAGCGGCAGTGCGTTTGATTGCAAAGTTACCAACGATCGTTGCTGCGTTTCACCGTATTCGTCGCGGACAGGACCCAGTTGCTCCTCGCGACGATCTTAGTCATGCGGCCAACTTCTTATACATGTTAAACGGCGAAGAACCAGACCCGCTTGCCGCACGCGTCTTCGACGTTTGCTTAATTCTGCATGCCGACCACGAAATGAACGCATCTACCTTTACTGCACGTGTAGTTGGCTCAACCTTGGCCGATCCTTATTCAGCAATCGCCGCAGCTATCGGCGCTCTTTCGGGGCCTTTACACGGTGGAGCTAACGAGGAAGTGTTAAAGATGCTGGAAGAGATTGGAAGTCCGGATAACGTGGAGCCGTGGCTGGAGGAAAAGTTGGCTCGTAAGGAGCGTATCATGGGCTTCGGACACCGTGTTTATAAAACCTATGACCCCCGCGCCAAAATTCTTAAAAAAATGGCCGAGCAATTGTTCGAAAAACACGGGTCAACGCCTTTATATGACATTGCCCAAGAACTTGAGAAGGTTGCAGCTGAACGTCTGGGGCCCAAGGGGATTTACCCTAACGTCGACTTCTACAGCGGAATTGTATATCAGTCAATGGGGATCCCAACGGACTTATTTACGCCTATTTTCGCGATCGCGCGTATTGCCGGTTGGACAGCGCATTGGTTAGAGCAACTTGAAGACAATCGTATCTTCCGTCCTCGTCAGATCTATGTGGGCCCCACTGACCGCTCTTACGTACCCATCGATGAGCGCCT |
| Altall Anc 2/3 | ATG GCA ACA ATG GAG TAC AAA CCA GGT CTG GCC GGG GTC GTT GCG GCT GAG TCG TCC ATT TCT TAC ATT GAT GGG CAA GAG GGC ATT TTA CGC TAT CGT GGT TAC CCA ATT GAG GAG TTG GCC GAA CAC TCC TCA TTT GAA GAG GTG GCG TAT CTT CTG CTT TTC GGT GAA TTG CCC ACC AAA GAA GAA CTT GAA GAG TTC GAT CAC GAG TTA AAG CAT CAT CGT GCA ATC CCA GAG CAA ATT ATT GAC CTG TTA AAA AAC CTT CCT AAA GAT GCT CAC CCC ATG GCT GCA CTT CAA ACG GCC GTG GCT GCA CTT GGT ATG TTC TAT CCG GAT GCA GAC GAT ACT GAC CCT GAA GGA AAT TAC GAA GCA GCC ATT CGT TTG ATT GCA AAA CTG CCC ACA ATT GTT GCC GCG TTT CAT CGC ATC CGC CGT GGC CAA GAC CCC ATC GCG CCA CGC GAT GAT CTT GGG CAT GCC GCC AAC TTT TTG TAT ATG CTT AAC GGA GAG GAG CCC GAT CCC GTG GCT GCC AAA GTG TTT GAT GTC TGC TTG ATC TTA CAT GCT GAG CAC GAG ATG AAC GCT TCT ACC TTT ACG GCC CGC GTA GTG GGT AGC ACG CTT GCA GAC CCT TAC TCC GCT ATC GCT GCT GCT ATT GGT GCT CTT TCC GGC CCT CTT CAC GGC GGA GCC AAC GAG GAG GTG CTT AAG ATG TTA CAG GAA ATT GGC AGT CCT GAA AAC GTC GAA CCC TGG ATC GAG GAA AAG TTG GCA CGC AAG GAG AAA ATT ATG GGT TTT GGA CAT CGC GTA TAC AAG ACT TAC GAC CCC CGT GCT AAA ATT TTG CAG AAA ATG GCG GAA CAG CTG TTC GAG AAG CAT GGT AGT ACT CCC CTT TAT GAC ATT GCA CAG GAA TTG GAA CGC GTC GCC GCG GAA CGT CTT GGT CCC AAG GGT ATC TAT CCG AAT GTA GAT TTC TAT TCG GGA ATT GTA TAT CAA TCC ATG GGC ATC CCC ACT GAT TTG TTC ACA CCC ATT TTT GCA ATT GCT CGT GTT GCA GGC TGG ACT GCG CAC TGG TTA GAA CAA TTA GAG GAC AAT CGC ATT TTT CGT CCA CGT CAG GTT TAT GTG GGG CCG ACC GAC CGC TCC TAT GTT CCA ATT GAT CAA CGC |
| Anc 3a | ATG GCG AGT ATG GAA TAC ACA CCC GGT TTA GCC GGC GTC GTT GCC GCG GAG TCC AGC ATC TCG TAC ATC GAT GGC CAA GAA GGT ATC TTA CGC TAC CGT GGA TAC CCT ATC GAG GAG CTT GCG GAA CAC AGC ACA TTC GAG GAA GTC GCA TAC CTG CTT TTG TTT GGG GAA CTG CCT ACG CGT GAC GAG CTT GAA GAG TTT GAC CAC GAG CTG AAG CAC CAT CGT GCT TTG CCA GAG CGC ATT ATT GAC TTA TTG AAA AAT CTG CCT AAG TCG GCT CAC CCA ATG GCC GCC CTG CAA TCG GCA GTC GCA GCT TTA GGG ATG TTC TAT CCT GAT GCA GAT GTA ACA GAT CCC GAG GGC AAT TAC GAG GCA GCA GTT CGC CTG ATT GCT AAA TTG CCA ACT ATT GTA GCT GCT TTC CAC CGT ATT CGC CGC GGC CAA GAC CCT ATC GCA CCC CGT GAC GAC CTT GGT CAC GCT GCT AAC TTT CTT TAC ATG CTG AAT GGT GAG GAA CCG GAT CCT CTT GCT GCA CGT GTC TTC GAT GTC TGT CTT ATT TTG CAC GCA GAG CAC TCT ATG AAC GCT TCC ACT TTC ACA GCC CGT GTA GTT GGA TCT ACG TTG GCA GAC CCA TAC TCG GCG ATC GCT GCC GCG ATT GGC TCA CTG AGT GGT CCC TTG CAT GGC GGC GCG AAT GAA GAA GTC TTA CAG ATG CTG GAA GAA ATC GGA AGC CCT GAT AAT GTC GAG CCT TGG TTG GAG GAA AAA TTA GCG CGT AAA GAA AAA ATC ATG GGA TTT GGC CAT CGT GTC TAC AAG GTG AAA GAC CCT CGT GCT ACC ATT TTA CAG AAG ATG GCA GAA CAA CTT TTT GAG AAA CAT GGC TCA ACT CCG TTA TAT GAC ATC GCC CTT GAG CTT GAG AAG GTA GCG GCA GAG CGT TTG GGC CCC AAA GGC ATT TAT CCT AAT GTC GAT TTC TAT TCA GGA ATT GTA TAC CAA AAA ATG GGG ATC CCA ACT GAT CTG TTC ACA CCG ATT TTT GCA ATC GCA CGC GTT GCC GGA TGG ACC GCG CAT TGG TTA GAG CAA TTG GAA GAC AAC CGT ATC TTT CGT CCC TCA CAG ATT TAC GTG GGC CCA ACT GAT CGT TCA TAT GTA CCT ATT GAC GAA CGT |
| Altall Anc 3a | ATG GCG TCG ATG GAG TAC ACA CCA GGA CTG GCT GGA GTT GTT GCT GCC GAA TCC TCC ATC TCG TAC ATC GAC GGC CAG GAG GGC ATC CTG CGC TAT CGC GGC TAC CCC ATC GAG GAG CTG GCC GAA CAC TCT TCC TTT GAA GAG GTC GCA TAC TTA CTT TTG TTT GGT GAG TTG CCA ACC AAG GAG GAG TTA GAC GAG TTT GAT CAC GAG TTA AAG CAT CAC CGT GCT ATC CCA GAG CGC ATT ATT GAC TTA TTA AAA AAC TTG CCG AAA TCG GGT CAT CCT ATG GCA GCC TTA CAG GCC GCT GTG GCG GCA CTG GGT ATG TTT TAC CCG GAC GCG GAC GTC ACA GAC CCC GAA GGC AAT TAT GAG GCG GCA ATC CGT TTG ATC GCA AAG TTA CCT ACT ATT GTT GCG GCG TTT CAT CGC ATT CGC CGT GGG CAA GAT CCT GTT GCC CCT CGT TCG GAT TTA GGC CAT GCG GCA AAC TTT TTG TAT ATG CTG AAT GGC GAA GAA CCT GAC CCA CTT GCG GCT AAG GTA TTC GAT GTG TGC TTA ATT TTA CAT GCG GAA CAT TCA ATG AAC GCG AGT ACA TTC ACG GCT CGT GTC GTG GGT TCT ACA CTG GCA GAC CCC TAT TCG GCA GTT GCC GCT GCA ATC GGG TCT CTT TCA GGG CCT CTG CAC GGT GGC GCA AAC GAG GAA GTC CTT CAG ATG CTG GAG GAG ATT GGG TCT GCC GAA AAC GTA GAG CCC TGG TTA GAA GAT AAG TTG GCT CGC AAG GAG AAA ATC ATG GGC TTC GGA CAC CGT GTC TAC AAA GTT AAG GAC CCC CGT GCT ACG ATT TTA CAG AAA ATG GCA GAG CAG CTG TTC GAA AAG CAT GGA AGC ACT CCT CTT TAC GAC ATC GCA TTG GAA CTG GAA CGT GTG GCT GCG GAA CGC CTT GGA CCT AAA GGC ATT TAC CCG AAC GTT GAC TTT TAC TCT GGA ATC GTT TAC CAG AAA CTT GGC ATC CCT ACA GAT CTT TTT ACC CCT ATC TTC GCC ATC GCG CGT ATT GCA GGT TGG ACC GCA CAT TGG CTT GAA CAA TTA GAG GAC AAT CGT ATC TTT CGT CCC ACC CAA ATC TAT GTG GGA CCG ACC GAC CGT TCC TAC GTC CCG ATT GAC GAA CGC |
| Anc 3b | ATG GCG GCC ATG GAA TAC ACT CCA GGC CTT GCG GGA GTT CCA GCT GCG GAG TCA AGC ATC TCC TAT ATC GAT GGC CAA CGT GGT ATT TTG CAA TAC CGT GGC TAC CCC ATT GAG GAA CTG GCT GAG CAT TCT ACA TTC GAG GAG ACA GCC TAT CTT CTT TTG TTC GGG GAA CTG CCA ACC AAA GAT GAA TTA GAG GAG TTC GAT CAC GAG CTG AAG CAC CAC CGT CGC CTT AAA TAT CGC ATT ATC GAC CTG TTA AAA AAT TTA CCT GAA AGT GGG CAT CCT ATG GAC GCC CTT CAA GCC GCG GTT GCT GCT CTG GGT ATG TTC TAC CCT GGG CGC GAC GTT ACA GAT CCT GAA AAT AAT TAC GAA GCT GCT GTT CGC CTT ATC GCT AAG CTT CCC ACC ATC GTG GCT GCG TTC CAC CGC ATT CGT CGT GGC GAT GAT CCT ATC CCT CCC CGC GAT GAC CTG GGA CAC GCC GCG AAT TTT TTA TAT ATG TTG AAT GGC GAA GAG CCT GAT CCG CTT GCC GCT CGT GTA TTC GAC GTC TGC CTT ATT TTA CAT GCA GAG CAT AGC ATG AAC GCC TCC ACC TTC ACT GCT CGT GTA ACT GGC TCG ACT CTG GCC GAC CCA TAC TCG GTT GTA GCT TCT GCC ATC GGT AGC CTG TCC GGC CCG CTT CAC GGT GGG GCA AAC GAG GAG GTG CTT CAG ATG TTA GAG GAG ATT GGC TCC CCT GAG AAT GTA CGT CCT TGG TTG GAG GAG AAA TTG GCA CGT AAG GAA AAA ATT ATG GGT TTC GGA CAC CGC GTG TAT AAG GTA AAA GAC CCC CGT GCG ACC ATC TTA CAG AAA TTA GCT GAA CAA TTA TTT GAA AAA TAT GGC TCA ACA CCA TTA TAT GAC ATT GCT CTT GAA TTG GAA CGT GTC GCC GCG GAG CGT TTG GGA CCG AAA GGC ATC TAT CCG AAT GTC GAT TTC TAT AGT GGC ATC GTT TAC CAG AAG ATG GGT ATC CCC ACG GAT TTA TTT ACG CCC ATC TTT GCC ATC GCT CGT GTA GCG GGA TGG CTG GCG CAC TGG CTT GAA CAG CTG GAA GAC AAC CGT ATC TTT CGT CCG TCT CAG ATC TAT GTC GGC GAG ACC GAT CGT TCA TAC GTC CCT ATC GAT GAG CGC |
| Altall Anc 3b | ATGAGTTCCATGGAGTATACGCCCGGATTGGCAGGCGTCCCAGCAGCGAAGTCCTCCATCTCGTATATTGACGGGCAAAAAGGCATCTTGGAGTATCGCGGGTACCCAATCGAGGAATTAGCTGAGCATTCTAGCTTTGAGGAGGTAGCCTATTTGTTACTGTATGGCGAACTGCCGACTCGTGAAGAACTTGACGAATTCGACCACGAATTGCGCCACCACCGCCGTATCAAGTACCGCATTATTGATATGATGAAAAATCTGCCCGAGTCGGGACACCCAATGGATGCCTTGCAAGCAGCCGTAGCCGCACTTGGTATGTTCTACCCTTCGGCAGATGTAACCGATCCCGAGAATAATCACGAGGCAGCTATCCGCCTGATCGCGAAGCTGCCTACTATTGTGGCAGCGTGGCACCGTATGCGTCGCGGTGACGACCCTATTGCACCTCGCGACGATTTAGGGCACGCGGCAAATTTCCTGTATATGTTAAACGGGGAAGAACCAGACCCGTTAACCGCTAAAATCTTTGATGTTTGCTTGATTCTGCACGCGGAGCATACGATGAACGCCAGTACATTCACTGCGCGCGTAACCGGAAGTACGTTAGCGGATCCGTATTCGGTAATCGCCTCCGCCATCGGCTCCCTTAGCGGCCCATTGCACGGAGGTGCCAATGAAGAAGTGTTAGAGATGCTGGAGGAGATTGGGTCGGCAGAGAACGTGAAACCTTGGTTAGAAGATAAGTTGGCGCGCAAGGAGAAGATCATGGGGTTCGGACACCGCGTATATAAAGTCAAGGACCCCCGTGCGACTATTCTGCAGAAGCTGGCCGAACAATTATTTGAAAAGTTTGGGTCCAGTCCCTTGTACGATATTGCTGTGGAACTGGAAAAAGTTGCAGCAGAACGCTTGGGTCCAAAAGGCATTTACCCTAATGTAGATTTCTACTCAGGCATTGTATATCAAAAACTTGGCATTCCAACCGATTTGTTTACACCCATTTTCGCTATTGCTCGTGTTGCCGGATGGCTGGCCCATTGGTTGGAGCAGTTGCAGGATAATCGTATCTTTCGCCCAACCCAGATTTACGTTGGAGAAAGTGACCGTTCCTATGTTCCGATCGAACAACGT |
| Anc 2 | ATG AGT ACC TCG AAG AAC ACC TTA ACG CTT ACG GAT AAC CGC ACT GGT AAG ACC TAC GAG TTA CCC ATT GAG GAC GGT ACT ATC CGC GCA ATT GAC TTA CGC CAA CTG AAA GTG GAT TTT GGG TTG ATG ACG TAT GAT CCA GGC TAC ACC AAT ACT GCC TCG TGT AAA TCA GCA ATT ACC TAC ATT GAT GGA GAT AAA GGC ATT CTG CGT TAC CGC GGG TAC CCC ATC GAG CAA TTG GCA GAG AAA AGC TCG TTC CTT GAG GTG GCC TAC TTG CTT ATC TAT GGC GAG TTG CCT ACA AAG GAA CAG CTG GAT GAA TTT GAG TAC GAG ATT ACG CAT CAT ACT TTT ATC CAT GAG AAT ATT AAG AAA TTC ATG GAC GGG TTC CCA CGT GAC GCG CAT CCC ATG GCC GTT CTG TCT TCC GCA GTG GGG GCC TTA TCG ACC TTC TAT CCC GAC GCA AAG GAC ATT AAT GAT CCA GAG CAA CGC CAC CTG GCG ATC GTA CGC TTG ATT GCC AAG ATG CCT ACA ATT GCC GCG TTC GCC TAT CGT CAT TCA ATC GGG CAG CCT TTC GTT TAT CCG GAC AAC GAT TTG TCC TAT GCA GAG AAC TTT TTG TAC ATG ATG TTT GCC ATG CCT GAA GAG AAG TAT GAG CCC GAT CCT GTT TTA GCC CGT GCC CTG GAC GTT CTT TTC ATT TTA CAT GCG GAC CAC GAA CAG AAT TGC AGT ACT AGT ACA GTT CGC CTG GTC GGT TCT TCC CAG GCG GAC CCA TAT AGT GCT ATC GCC GCT GCC ATT GCC GCT TTA TAC GGC CCG CTG CAT GGT GGC GCT AAT GAG GCA GTT TTA CGT ATG CTG GAG GAA ATC GGC TCG GTA GAC AAC GTG CCT GAA TTT ATT GAA CGC GCC AAA GAC AAG AAA GAC CCT TTC CGT CTG ATG GGG TTC GGA CAT CGT GTA TAC AAG AAT TAC GAT CCC CGC GCG AAA ATC ATC AAG AAG ATG GCA CAT GAG GTT TTT GAA AAA ACA GGT AAG AAC CCT CTT CTT GAC ATC GCA ATG GAG TTG GAG CGT ATC GCC CTT GAA GAC GAG TAC TTC GTT GAG CGT AAG TTG TAT CCG AAC GTA GAT TTT TAC TCA GGG ATT ATT TAT CAG GCG ATG GGC ATT CCG ACC GAC ATG TTC ACC GTC TTA TTC GCA ATC GGA CGC ACT CCA GGT TGG ATT GCT CAG TGG AAG GAA ATG CTG GAG GAC CCA GAA CAG AAG ATT GCT CGC CCC CGC CAA ATC TAC GTG GGA GAG ACG GAG CGT GAC TAC GTA CCT ATC GAT CAA CGT |
| Altall Anc 2 | ATGGCAACCTCGAAAGCAACTCTGACCATCACCGATAATCGCACAGGTAAGACTTACGAGATTCCAATCGAGGACGGTACGGTGAAGGCAATGGATATTCGTAAGATCCGTGCTGAAACTGGTTACATGTCTTTCGATCCGGGCTTCATGAATACTGCTTCATGCCGCTCTGCGATTACTTATATTGATGGAGACAAGGGAATTTTACGCTACCGCGGTTATCCTATCGAACAATTAGCAGAAAAAAGTTCTTTCTTAGAAGTGGCCTACTTGTTGATTTACGGTGAGTTGCCAACCAAGGACCAGTTGGAAGAGTTCGAACATGAAATCACACACCACACATTTCTTCACGAAAATTTAAAGAAGTTCTTCGACGGCTTCCCCCGCAACGCTCACCCCATGGCTATGTTGTCATCGGCTGTGGCTGCTTTGTCCACATTCTACCCGGATAGCAAAGACGTAGATGATCCTGAGGCACGCCATTTGCAGGTCGTACGCCTGATTGCTAAAATTCCTACCATTGCGGCATTCGCGTACCGTCACAGCATCGGACAGCCATACGTGTATCCCCGTAATGACCTTTCCTACGCCGAAAATTTCTTGTATATGATGTTCGCAATGACAGCAGAGAAATATGAACCAGACCCCGTACTTGCGAAAGCCCTGGACGTGTTGTTCATCTTACATGCAGATCATGAACAGAACTGCTCAACCTCTGCTATGCGCTTAGTCGGTTCATCACAGGCCGACCCCTACAGCGCCGTTGCAGCGGCAATTGCGGCTTTGTGGGGCCCACTGCACGGCGGTGCGAATGAGGCGGTTCTTAAAATGTTAGAGGAAATTGGTAGCGTTGAGAACGTGCCGGACTTTATTGAGAAGGTCAAGGAGAAAAACGATGAATTCCGCTTGATGGGCTTTGGACACCGCGTTTATAAGAATTATGACCCTCGTGCCAAAATTATCAAGAAAATTGCTGACGAGGTTTTCGAGGAGTTAGGCAAAGATCCGCTTCTTGATATCGCAATGGAGTTAGAAAAAGTAGCTCTGGAAGATGAATATTTTATCAGCCGTAAGCTGTATCCGAACGTGGATTTCTACTCCGGAATCATTTACCAAGCCATGGGTATTCCCACCGACATGTTCACCGTGTTATTCGCCATTGGGCGTACGCCGGGCTGGTTGGCACAGTGGCAGGAGATGCTGGAAGATCCCGAGCAAAAGATCGCACGCCCTCGTCAAATTTATACCGGCGAGGACGAACGTGACTACGTGCCGATGGACCAACGT |
| Anc 2a | ATG AGT ATG TCG AAG AAT ACC TTA ACG ATT ACT GAC AAC CGT ACT GGA AAG ACC TAC GAG ATC CCG ATC GAG GAC GGG ACG ATT CGT GCA ATG GAC TTG CGC CAA ATC AAA GTA GAC GAC GAT GAT TTC GGG TTG ATG AGC TAC GAC CCG GGT TAT ATG AAT ACT GCC TCT TGC AAG TCC GCA ATT ACT TAC ATC GAT GGC GAC AAG GGC ATT CTT CGC TAT CGT GGG TAT CCC ATC GAG CAA TTG GCC GAG AAA TCG TCG TTC CTT GAA GTA GCC TAT CTG CTT ATT TAT GGG GAA TTG CCC ACC AAG GAA CAA TTG GAT GAA TGG GAG TAT GAG ATT ACA CAT CAT ACA TTC ATC CAC GAG AAC ATT AAA AAA TTT ATG GAC GGC TTT CGC TAC GAC GCC CAC CCA ATG GGG ATG TTG GTG AGC TCT GTA GGT GCG CTG TCA ACC TTC TAT CCT GAC GCA AAG GAC ATT AAT GAT CCA GAA AGC CGC CAC TTA CAG ATC GTC CGC TTA ATC GCT AAG ATT CCT ACT ATC GCG GCT TTT GCT TAT CGT CAT TCC ATT GGA CAG CCT TAT GTC TAT CCC GAC AAT GAT CTT TCT TAC GCG GAA AAC TTT TTG TAT ATG ATG TTC AAA ATG ACC GAG TTG AAG TAC GAG CCG GAC CCC GTT TTG GCT CGC GCG TTG GAT GTA TTA TTC ATT CTT CAC GCA GAC CAT GAA CAA AAC TGC TCA ACG AGC GCT ATG CGC TCT GTC GGC TCC AGT CAA GCG GAC CCG TAT TCA GCG GTG GCT GCT GCA GTG GCG GCG TTA TAT GGC CCA CTT CAT GGC GGG GCT AAC GAG GCA GTA CTT CGC ATG CTT GAG GAA ATT GGT TCT GTA GAC AAC GTC CCT GAG TTT ATT GAG CGC GTG AAG GAA AAG AAG GGG GAA TTC CGT CTG ATG GGT TTT GGG CAT CGC GTG TAT AAG AAT TAC GAC CCA CGT GCC AAG ATT ATT AAG AAA ATC GCA CAT GAG GTC TTT GAG GTT ACG GGC AAG AAC CCA TTA TTG GAT ATT GCA GTG GAA TTA GAA CGC ATT GCC CTG GAA GAT GAA TAC TTC GTG AGC CGC AAA TTG TAC CCA AAC GTT GAT TTC TAC TCC GGC ATT ATT TAT CAG GCT ATG GGA TTC CCA ACC GAT ATG TTT CCG GTC CTG TTT GCT ATT GGT CGC ACG CCT GGG TGG TTA GCC CAG TGG CAA GAG ATG TTA GAA GAT CCA GAG CAG AAA ATC GCT CGC CCT CGC CAG ATT TAC GTC GGA GAA GAC GAG CGC GAT TAC GTG CCC ATC GAT CAA CGC |
| Altall Anc 2a | ATGACGACTTCGAAAGATACCCTTACAGTCACAGATAACCGCACGGGAAAGACGTACGAAGTGCCCATTGAAGACGGAACAATTCGTGCGATGGACCTGCGTCAAATTAAAGTAAACGATGATGATTTTGGACTGATGGCTTATGACCCAGGCTTCACCAATACAGCGTCGTGTCGTAGTGCAATCACCTACATCGATGGCGACAAGGGGATTTTGCGCTACCGCGGGTACCCTATTGAGCAATTGGCAGAAAAGAGCAGTTTTCTTGAGGTGGCTTATTTATTAATTCATGGTGAGCTTCCCACCAAGGACCAGTTAGAACAGTGGACCTATGAGATCACTCACCACACCTTCGTACACGAGAATCTTAAGAAGTTTATGGACGGTTTCCGTTATGACGCGCACCCAATGGGAATGTTAGTCTCAACAGTCGCCGCCTTATCTACTTTCTATCCTGAGGCTAAAAACATTGACGATCCGGAAGCACGTCACTTGCAGATTGTTCGTCTGATTGCAAAAATGCCGACGATCGCCGCCTTTGCTTATCGCCACTCAATTGGGCGTCCGTTCGTCTACCCTGACAACGATTTAAGCTATGCCGAAAACTTCTTGTATATGATGTTTAAGATGAGTGAGTTGAAGTATGAGCCAGATCCTGTGCTGGCCAAAGCCTTGGATGTTTTATTTATTCTTCACGCCGACCATGAGCAGAACTGCTCTACCTCTGCTGTTCGCGCAGTTGGTAGTTCACAGGCCGACCCCTATAGCGCTATCGCGGCGGCCATCGCTGCTCTTTACGGACCACTTCATGGTGGCGCAAATGAAGCCGTTCTTCGCATGCTTGAAGAGATCGGATCAGTAGAAAATGTACCCGACTTTATTGAAAAAGTAAAGGCACGCAAAGGAGAATTTCGTTTAATGGGTTTCGGGCACCGCGTTTATAAAAACTATGATCCTCGCGCGAAAATTATCCGTAAGGTCGCCGATGAGGTCTTTGAAGTGACAGGTCGTAATCCCCTGTTGGACATCGCTATGGAGTTAGAAAAAGTGGCGTTGGAGGATGAATACTTCATTAGTCGCAAGTTATATCCCAACGTGGACTTTTATTCTGGATTAATTTATCAAGCAATGGGCTTCCCCGTTGATATGTTTCCCGTGCTTTTTGCGATCGGGCGCACCCCAGGTTGGTTAGCTCAATGGAAGGAAATGTTACAGGACCCTGAACAGAAAATCGCCCGTCCACGCCAGATTTATACAGGCGAAGACAAACGTGACTATGTTCCCATGGACCAGCGC |
| Anc 2b | ATG TCC TCG AAG AAG GCG ACA TTG ACG TTA ACT GAT AAC CGC ACG GGC AAA ACA TAT GAG TTA CCG ATC TTA GAC GGA ACA GTA GGT CCC AAG GCT ATT GAT ATT CGT AAA TTA CGT GCT GAT ACT GGA TAT ATG ACG TTC GAT CCA GGG TAC ACG AAT ACG GCA AGT TGC AAG TCG GCC ATT ACC TAT ATT GAT GGA GAC AAA GGC ATC TTG CGT TAT CGC GGT TAC CCA ATT GAG CAG CTG GCC GAA AAA TCG TCA TTT CTG GAG GTC GCC TAC CTG TTA ATC TAT GGT GAA TTG CCG ACT AAA GAA CAG TTA GAC GAA TTT GAG CAC GAG ATC ACC CAT CAT ACA TTC ATC CAT GAA AAC TTG AAG AAG TTT TTC GAC GGC TTC CCT CGC GAT GCA CAC CCT ATG GCT GTG CTG TCG TCG GCT GTT GGA GCA TTG AGT ACA TTT TAC CCT GAC AGC TTG GAC ATC AAT GAC CCC GAA CAG CGC AAT TTG GCA GTT GTG CGT TTG ATC GCG AAG ATG CCC ACA ATC GCC GCT TTC GCA TAT AAG CAC AGC ATC GGA CAG CCG TTC GTC TAT CCG GAT AAT GAT CTG AGC TAC GCG GAA AAC TTT CTG TAC ATG ATG TTC GCG GTC CCG GCC GAG GAA TAT GAA GTT GAT CCA GTC CTT GCG AAA GCA CTG GAT ATG TTG TTT ATC CTT CAT GCA GAC CAC GAA CAG AAT TGC AGT ACT TCC ACG GTT CGT CTG GTT GGC TCC TCA CAA GCT AAC CCG TAT GCG GCC ATT GCG GCG GGA ATC GCT GCG CTT TGG GGG CCC TTA CAT GGC GGG GCT AAC GAA GCT GTC TTG AAA ATG CTT GAG GAG ATC GGT TCC GTA GAC AAC GTC CCC GAG TTT ATT GAG CGT GCT AAA GAT AAG AAC GAC CCC TTC CGC TTG ATG GGT TTT GGA CAT CGT GTT TAC AAA AAT TTT GAT CCC CGC GCT AAA ATC ATC AAA AAG ACG GCA GAT GAA GTA TTG GAA AAA CTT GGT ATT GAT GAT CCA CTT TTG GAT ATC GCC ATG GAA TTG GAG AAA ATT GCA CTG GAA GAT GAA TAC TTT GTT GAG CGC AAG CTG TAC CCA AAT GTT GAT TTT TAC AGT GGC ATT ATT TAT CAG GCT ATG GGT ATC CCT ACA GAC ATG TTC ACG GTC CTG TTT GCA ATT GGC CGT ACT CCG GGC TGG ATC GCG CAA TGG AAG GAA ATG TTG GAA GAC CCA TCC CAA AAA ATC GGA CGT CCT CGC CAA ATT TAC ACG GGC GAA ACC GAG CGT GAC TAT GTC CCT ATT GAT CAG CGT |
| Altall Anc 2b | **ATGGCTGCCGAAAAGGCGACGCTGACATTGACGGATAATCGTAACGGGAAGTCTTACGAGCTGCCGATCATTGAAGGTACCATCGGTGCCGACGCGATTGACATCCGCAAACTGCGCGCAGAAACGGGGTATTTCACCTATGATCCGGGTTTTGCGAATACTGCTTCTTGCAAGTCGAAAATCACTTACATTGATGGTGATAAGGGCATCTTGCGCTATCGTGGCTACCCGATCGAGCAACTTGCGGAGAAGTCGAGTTTTTTGGAAGTAGCGTATCTTCTGATCTACGGTGAACTTCCGACCCGTGACCAGTTGGAGGACTTCGAAAATGAGATCACACATCATACTCTGCTTCATGAGGATATGCGTAAATTTTTTGACGGCTTTCCACGCAATGCACACCCAATGGCGGTTTTATCGTCTGCAGTTTCGGCCTTGTCGGCCTTCTATCCAGATTCGATGGATGTAAACGACCCGGAACAGCGTCACCTTTCAATCATCCGCTTGATTGCCAAGATCCCCACTATCGCGGCGTTTGCATATCGCCACTCCATTGGCCAACCGTTCGTATATCCACGCAATGACCTGTCTTACGCTGAAAATTTTTTGTACATGATGTTCGCGATGCCAGCTGAGGAATATGAAGTTGACCCCGTCTTAGCCCGTGCCCTTGACGTCTTACTTATCTTGCACGCCGATCACGAGCAAAACTGCAGCACCTCGACGGTTCGCCTGGTCGGTTCCTCACAAGCGAATCCCTACGCGGCGATCTCCGCTGGGATTTCCGCCCTGTGGGGTCCTCTTCATGGTGGGGCTAATGAGGCGGTGTTAAAAATGCTGGAAGAGATTGGCTCAGTCGAGAATGTTCCCGAATTTATTGAAAAGGTTAAAGATAAAAATGATCCTTTCCGCCTTATGGGTTTTGGTCATCGTGTTTATAAGAATTACGACCCGCGTGCGAAGATCATTAAGAAAATGGCACACGAGGTACTGGAGGAACTGGGGGTGAATGATCCTCTGTTAGATATCGCCAAGGAGTTAGAACGTGTGGCACTTAACGATGAGTACTTTATCGAGCGCAAGCTTTACCCTAACGTAGATTTCTACTCAGGGATTATCTACAAGGCCATGGGCATCCCGACCGATATGTTCACCGTGTTATTCGCGATTGGGCGCACGCCGGGTTGGATCGCGCAGTGGAAAGAGATGCTGGAAGATCCTTCTCAGAAAATTGGGCGCCCCCGCCAAATCTACACCGGCGAAACGCAACGCGATTACGTGCCTATGGATCAACGC** |
| Anc 2c | ATGTCCGAAACAGCTACGCTTACTCTTGATGGCAAGACATACGAACTGCCCATTATTGAGGGAACGGAAGGTGAAAAAGCAATTGACATTCGTAAATTACGCGCTGAAACGGGTTATATCACCTTTGATCCTGGATACGCCAATACGGCCTCGTGCAAGTCCGCCATCACATATATTGACGGGGATAAGGGAATCCTGCGTTACCGTGGGTACCCTATCGAGCAGTTAGCGGAGAAGTCCTCTTTCCTGGAAGTAGCCTATTTATTGATTTATGGTGAATTGCCCACAAAAGAACAATTAGACGAATTCCAAAATGAGATTACCCATCATACGCTGATTCACGAGGATTTAAAGAAGTTTTTCGATGGCTTCCCACGCAACGCGCACCCAATGGCAGTGTTATCCTCGGCGGTTTGCGCGCTTTCGACCTACTACCCGGACAGTCTTGACCCTAACGACCCTGAGCAAATTAACCTGTCGATCGTTCGCCTTCTTGCTAAACTGCCAACGATCGCTGCCTTCGCCTACAAAAAATCCATTGGTCAACCGTTTGTTTACCCCGACAATGATCTTTCCTATTGCGAAAATTTCCTGTACATGATGTTCGCGGTTCCCGCCGAACCCTACGAAGTCGATCCTGTCGTAGCTAAAGCGTTGGATATGTTATTCATTTTGCACGCAGATCATGAGCAAAATTGCTCCACGAGTACTGTACGCCTTGTCGGGTCTTCGCAAGCTAATCTTTATGCAAGTATCAGTGCTGGGATTTCGGCACTTTGGGGGCCGTTGCATGGAGGAGCAAACCAGGCGGTTCTGGAAATGTTGGAAGAGATTCACGCAGATGGAGGGGACGTTAAGGAATTCATTGAGAAAGCGAAGGATAAGAATGATCCATTCCGTTTAATGGGATTTGGTCATCGTGTATATAAGAATTTTGACCCGCGTGCGAAAATCATCAAGAAAACAGCCGATGAGGTGTTAGAAAAGCTGGGGATTAATGATCCTCTTCTGGACATTGCGAAAGAATTAGAAGAAGTGGCGCTTAACGACGAATATTTCGTCGAACGCAAATTATATCCGAACGTCGATTTTTATTCAGGTATCATCTATCGCGCCATGGGGATCCCGACGGATATGTTCACCGTTCTTTTTGCATTAGGACGCTTGCCGGGATGGATCGCCCAGTGGAAAGAAATGCGTGAGGATCCGTCGACTAAAATCGGGCGTCCTCGTCAGATCTATACAGGAGAGACCGAGCGTGACTATGTGCCCATTGATCAACGCCTCGAGCACCACCACCACCACCACTGA |
| Anc 2d | ATGGCAGCTAAGAAGGCTACAGTTACGTTAACAGATAATCGTACTGGCAAATCATATGAGTTGCCGATTTTGGATGGGACGGTCGGACCTGATGTAATCGACATTCGTAAACTGTATGCTGACACTGGCTACTTTACATTTGACCCGGGGTACACATCTACGGCGTCTTGCAAATCAAAGATTACCTACATTGATGGGGACAAGGGAGTGTTGCGTTATCGCGGGTATCCGATCGAACAGTTGGCCGAGAAGAGTTCATTTTTAGAGGTTTGCTATCTTTTGTTAAATGGGGAATTGCCTACCAAGGAACAATTAGAGGAGTTTGAACACGAGATTACACATCATACATTTCTTCATGAGAATTTGCGTAAATTCTTTGATGGATTCCCGCGCGACGCGCACCCGATGGCGGTATTAAGTTCTGCTGTAGGGGCACTTTCAGCATTCTACCATGACAGTCTTGACATCAATGACCCGGAACAACGCAATATTGCAGCTCATCGCCTGATTGCAAAGATGCCCACAATCGCAGCGTTCGCATACAAACACTCGATTGGACAGCCGTTTGTTTATCCGGACAACGACTTGGGTTACGCCGAGAATTTTTTATACATGATGTTTGCTGTGCCCGCTGAGGAATACGAAGTCAACCCGGTTCTGGCACGCGCTTTGGACACTATCTTTATTCTGCACGCGGACCATGAACAAAACGCCAGTACGTCGACGGTGCGCTTGGTCGGGAGCTCAGGCGCAAACCCTTATGCGGCCATTGCAGCTGGCATCGCCGCACTTTGGGGACCCGCGCATGGTGGTGCAAATGAGGCTGTGTTAAAAATGCTGGAGGAGATCGGGAGTGTTGACAATGTACCCGAATTTATCGAACGTGCTAAGGATAAAAATGACCCCTTCCGTTTGATGGGTTTTGGTCACCGCGTATACAAGAATTTCGACCCACGCGCAAAGATTATGAAGAAAACAGCGGATGAAGTATTAGAAGAACTGGGCATCGATGACCCGCTTCTGGACATTGCTATGGAACTTGAGAAGATCGCGCTGGAGGACGAATACTTCGTAGAGCGTAAGCTGTATCCTAATGTAGACTTTTACTCTGGAATCATTTTAAAGGCGATGGGTATCCCAACCTCGATGTTTACTGTGTTATTCGCTATTGGTCGCACCCCTGGCTGGATTGCTCAGTGGAAGGAAATGATCGAAGATCCATCACAAAAAATCGGCCGTCCGCGTCAGTTATACACTGGTGCAACAGAGCGCGATTATGTACCCATTGACCAACGCCTCGAGCACCACCACCACCACCACTGA |
| *Archaeoglobus veneficus*  WP_156786146.1 | ATG CAC GAC GGG CTT CAA GAC GTC CTT GCA TGC AAG TCA TCC ATT TGT CGT ATC GAG ATG ATT GAT GGA AAG GCG ATT TTA GAG TAC CGT GGG TAC GAT ATT CAT GAG TTG GCC CAG CAT AGC ACT TTC GAG GAA GTT GCC TAC TTA TTA TTA TTC GGT GAA TTG CCA ACC AAA TCC GAA CTG GAG GCT TTT TCC GAA GAG TTA AAG GAG TTA CGC GAA TTG CCG CCC CAG ATC ATC GGA CTG TTA ACG CAT TTA TCC CCT TTT TCA CAC CCT ATG GTC GTC CTG CGC ACT GCT ATC TCT TAC CTG GGT ACT ATG GAT AAA CAC ATC CAC CAT AAA TCA CAT GAA AAG TCT TTG CAA AAG GCT AAA TCC CTG ATT GCT AAG TTC CCA ACA ATT GTC GCT TAC TTC CAT CGT ATC CGT TCA GGT CAA AAC TTA GTC CAC CCG TCA GAT GAA TTG AGC CAC GCT GCT AAT TTT TTA TAT ATG CTG CAC GGC GTA GAG CCT ACT AAA ACG GAA GCT AAA GCG ATG GAC CTG GAT TTA GTT TTA CAC GCA GAC CAT GAA CTT AAT GCT TCG ACG TTT GCA GCC CGT ATT GCT GCA TCG ACT TTA GCC GAC ATT TAT GCG TGC GTG GTC GCC GCA ACG GGA ACA CTT ATG GGA CCC TTG CAC GGG GGA GCA GCG CAG AAA GTT ATG GAA ATG TTG CGT GAG ATC GCG GTG CCT TGG CGT GCC GAA GAG TAT GTC AAG ATG AAA TTG GAA CGT GGA GAA CGT ATC ATG GGA TTC GGT CAT CGT GTA TAC CGT GGT GTC GTA GAC CCC CGT ACA ATC GAA CTG CGC GCA CTG GCC GAG AAG CTG GCT AAG GAA AAG GAG CCA AAG TGG TTC GAA ATT TCC CGC GCT GTC GAA GAA GCT GTA TAC AAA TAT AAG GGA CTT TTT CCC AAT GTG GAC TTT TAC TCT GCT TCC GTG TAT GCA AAT CTT GGC ATC CCC GAT GAC TTG TTC ATT AAT ATT TTC GCC ATC TCT CGC ATT TCT GGG TGG ACC GCA CAC ATC ATT GAA CAG TAT GAG AAT AAC CGC TTA ATT CGC CCA CGC GCA TTC TAC GTG GGA GAG GCT GGC CGT AAG TAT GTG CCA ATC GAT CAG CGT GGA GAT |
| *Bacillus subtilis*  WP_004398810.1 | ATG ACA GCG ACA CGC GGT CTT GAA GGG GTT GTA GCA ACA ACA TCA TCT GTT AGT TCT ATT ATT GAT GAT ACC CTT ACA TAT GTG GGG TAT GAT ATC GAT GAT TTG ACA GAG AAT GCA AGT TTT GAA GAA ATC ATC TAC TTG CTT TGG CAT CTA AGG CTG CCA AAC AAA AAA GAG CTT GAA GAG TTA AAG CAG CAG CTT GCA AAG GAA GCT GCC GTT CCG CAA GAA ATT ATT GAG CAC TTC AAA TCT TAT TCC CTC GAA AAT GTT CAT CCC ATG GCT GCT CTT CGG ACA GCA ATT TCA TTA CTA GGC CTG CTT GAC AGC GAG GCT GAT ACC ATG AAC CCG GAA GCC AAC TAC AGA AAG GCG ATC CGC CTT CAG GCA AAG GTG CCT GGC CTG GTT GCA GCG TTT TCC AGA ATT CGC AAG GGT CTT GAG CCT GTT GAA CCG AGA GAA GAT TAC GGT ATT GCG GAG AAT TTT CTT TAC ACA TTA AAC GGT GAA GAG CCT TCA CCA ATT GAA GTT GAA GCC TTC AAC AAA GCA TTG ATT TTA CAT GCT GAC CAT GAG CTG AAC GCG TCA ACG TTC ACA GCG AGG GTC TGC GTC GCA ACA CTT TCT GAC ATC TAT TCA GGC ATT ACT GCA GCA ATC GGC GCG CTG AAA GGG CCT CTT CAC GGC GGA GCG AAT GAA GGC GTT ATG AAA ATG CTG ACA GAA ATC GGT GAA GTG GAA AAC GCT GAG CCG TAC ATC CGC GCT AAA CTT GAA AAG AAA GAA AAA ATC ATG GGA TTC GGC CAC CGT GTG TAT AAG CAT GGT GAT CCG CGC GCG AAG CAT CTG AAA GAA ATG AGC AAG CGC CTG ACC AAC CTG ACA GGC GAA AGC AAA TGG TAT GAG ATG TCG ATT CGT ATA GAA GAC ATC GTC ACA TCA GAG AAA AAG CTT CCG CCT AAC GTT GAT TTC TAT TCG GCT TCT GTA TAT CAC AGC CTC GGT ATT GAC CAT GAT TTG TTT ACA CCG ATT TTT GCT GTA AGC AGA ATG TCC GGC TGG CTC GCT CAT ATT CTT GAG CAG TAT GAC AAC AAC CGT CTG ATC CGC CCG CGG GCA GAT TAC ACA GGC CCT GAC AAA CAA AAA TTC GTT CCG ATT GAA GAA AGA GCC CTC GAG CAC CAC CAC CAC CAC CAC TGA |
| *Deinococcus pimensis*  WP_027481744.1 | ATG ACA ACC ACA ATC GCC AAA GGG CTT GAA GGA GTG TTA TTT ACA GAA ACG AAA CTG ACG TTT ATT AAC GGG ACC GAG GGC GTC CTT ACA CAT CTT GGC ATT CCC ATC CAA GAG TGG GCG GAA AAA TCT ACC TTC GAA GAG TTG TCA TTC GCG CTT CTG CAT GGG AAG CTT CCT ACA CGT GAA GAA CTG GCT GCA TTC GAT GCC GAC TTA AAG GCT AAC CGC GCT GTG CCT GCT GAA CTT ATC ACT GCG ATT CGT GCA TAC CCC CGC GAT GTC CAT CCC ATG CAA GCG CTT CGT ACT GCT GCG TCA CAT CTG GGG TTA TTG GAT GCG GAC GCG GAA GAT ACA TCC GCA GAA GGG CGT TAC CGT ATT GGC GTC CGT ATG ATT GCT CAG TTC TCG ACG ATT ATT GCA GCA ATT GCG CGC GCA CAA GAC GGA CAA GAC CCA GTG GAG CCT CGC GCT GAT CTG ACT CAC GCG GGC AAT TTC TTG TAT ATG TTG AAC GGT CAA GAG CCC TCA GCC GAA CAG GCC CGC CTG TTT GAT ATT GCA CTT ATT CTG CAC GCG GAC CAT GGT ATG AAC GCC TCG ACA TTT ACA GCC ATT GCG ACG GCC AGC ACT TTA TCA GAT ATG TAC AGT AGT GTC GTC TCT GCT ATC GGA GCC CTG AAA GGT CCT CTG CAT GGA GGT GCT AAC GAA GCA GTC ATG GAC ATG CTT GAC GAG ATT GGA ACT GTT GAT GCG GCA GTA CCA TTC ATC ACG CAG AAG TTA GAT AAC AAG GAA AAA ATC ATG GGG GTC GGC CAC CGC GTG TAC AAG TAC TTC GAC CCG CGC AGT CGC GTC TTA CGC GAC TAT GCT GCT CAT GTC TCC GAA AAA CAA GGA AAG TCC CAC TAC TAT TCT ATT TTA GAA ACA ATT GAA AAG GAA ATC GTC GAA CGC CTT GGG GGT AAA GGG ATC TAT CCG AAC GTT GAT TTC TAT TCC GGA GTT GTA TAC TCG GAT CTG GGG ATT AAA AAG TCG TTT TTT ACC CCC ATT TTC GCT CTT GCC CGC ATT TCA GGC TGG GTC GCT AGT GTA ACA GAG TAC ACT GCC AAC AAC CGT TTA CTT CGC CCC GAT GCG GTT TAC GTA GGC GCG CGC GAC CAA CAC TAT GTC GAG ATT GAC GAG CGC AAG |
| *Deinococcus radiodurans*  WP_027479989.1 | ATG TCA AAT ATC GCC AAA GGC TTA GAA GGC GTG CTG TTT ACC GAA AGT AAA CTT ACA TTT ATT AAT GGG TCA GAG GGT ATC TTG ACA CAC CTT GGA ATC CCT ATT CAG GAA TGG GCT GAG AAA AGC ACT TTC GAA GAG TTG AGC CTT GCG TTG TTG GAT GCC AAA CTT CCA ACC GCG GAA GAA TTA GCC AAA TTT GAC GCG GAA CTT AAA GCG AAC CGT GCG ATT CCG GAC CAG TTA GTC GGA ATC ATC CGT GAT ATG CCT AAG GGG GTT CAC CCT ATG CAA GCA TTG CGT ACC GCA GTT AGT TAC TTA GGA CTT CTG GAC CCG CAG GCG GAA GAC ATC ACT CCA GAA GCA CGT CGC GCT ATT AGT ACC CGC ATG ATC GCG CAG TTT AGT ACT ATC ATC GCT GCG ATC AAC CGC GCT CAG GAG GGC CAA GAC ATT GTA GCC CCC CGC GCG GAT CTG ACC CAC GCC GGC AAC TTC TTA TAT ATG TTA ACT GGT AAT GAA CCG ACA CCT GAG CAG GCA CGT CTT TTT GAC ATC GCA CTG GTT TTA CAT GCG GAT CAT GGA ATG AAT GCA AGC ACG TTC ACA GCT ATT GCG ACT TCG TCC ACA TTG AGT GAT ATG TAC AGT TGC ATG GTT TCA GCG ATC GGG GCT TTG AAG GGT CCG CTT CAT GGC GGT GCC AAC GAA GCA GTA ATG ACT ATG TTA GAC GAG ATT GGA ACA GTA GAC AAG GCT GAA GCG TAC ATT ACC GGA AAG CTT GAT AAC AAG GAA AAA ATC ATG GGT GTT GGC CAT CGC GTC TAT AAG TAT TTT GAT CCT CGT AGC CGT GTG CTT CGT GAC TAC GCG GAG CAT GTT GCA AAT AAG GAG GGT AAA TCC AAT TAC TAC CAG ATT TTG GAA GCG ATT GAG AAA ATT ATT GTA GAT CGC ATG GGC GCG AAG GGA ATT TAT CCA AAT GTT GAC TTC TAC TCT GGT ACC GTA TAT TCT GAC CTT GGG ATT AAA AAG GAG TAC TTC ACT CCG ATC TTC GCC TTG GCG CGT ATT TCA GGT TGG TGT GCT TCA GTG ATT GAG TAC TCA CAA GAT AAT CGC CTG CTT CGC CCC GAC GCA GAA TAT ACT GGT GCG CGT GAT CAG CAT TAC GTC GAC ATC AAA GAC CGC CAA |
| *Nitrosopumilus maritimus*  WP_012215482.1 | ATG GAG ACG AAG AAT ATC GGA TTG CGT GGT ATT GAA GTT GCG GAT ACT CGT ATC TCC AAT ATT GAC GGA GAA AAA GGG AAA CTG ATC TAT CGT GGG TTC GAT ATC CTT GAC TTA ACG AAG AAT AGC ACA TTC GAA GAA ACC GCT TAC CTG CTT CTT TAT GAT AAC CTT CCT ACC AAG GCT CAG TTG GAC GAA TTC AAC CAA AAA TTA GTG GAG GCG CGC TAT ATC CCG AAA CAA ATG CAG AAG AAC ATG GGG AAC TGG CGT AAA GAC GCG GAC CCT ATG GAT ATG CTG CAG GCA TTC GTA TCG GCT TTG GCA GGC TAC TAC GAT GAA GAG TTC TCA AAC AAG GAA GCG AGC TAC GAG AAA GCA ATT AAC CTG ATT GCA AAA GTA CCC ACA ATC ATT GCG AGC TGG CAA CGT ATC CGT AAT GGC TTA CCG ATT GTT GAT CCG GAT TCG TCG CTG AGT CAC GCA GCG AAT TTC TTA TAT ATG ATG AGT GGC GAA AAA CCT GAT CCG GAG GTC GAA AAA GTA TTT GAT GTG TGC TTA ATC CTT CAT GCT GAC CAC ACA TTT AAC GCC TCC ACA TTC ACC GCC CGC CAA GTC GCC TCC ACA CGT GCG CAT ATG TAC TCT GCA TCT TCT GCA GCT ATC GGT GCC TTA TCG GGG GAG TTA CAC GGG GGC GCT AAC ACA GAG GTG ATG AAG ATG TTA CTT GAA ATT AAA GAG ATT GAT AAA GTA GAA CCA TGG ATC AAG GAA AAA ATG AGC GCG GGC GAG CGT ATT ATG GGG ATG GGG CAC GCG GTC TAC CGC ACT TAT GAC CCA CGC GCC CAA GTG TTG AAG GAG TTA TCG CGC AAG TTA GCT GAA AAA ACA AAA GAA CCC TGG TTC GAT ATG ACG GAA AAA GTA GAA ACT ACG ACC ATC AGC GAA ATG AAA GCA CAG AAG GGG AAA GAC ATC TAC CCT AAT GTG GAC CTG TAC TCT GCC TCG ATC TAC TAC ATG CTG AAG ATT CCC GTT GAC CTT AAC ACG CCT ATT TTC GCG ATT TCA CGC GTA GTA GGC TGG GCA GCG CAC ATT ATC GAA GAG AAG TTC GCC GAA GCT GCC CCG AAA CCT GCC CTT TAC CGC CCC AAA GCG ACG TAC GTG GGC AAA TAT TGC GGT CCC GAG GGA TGT GAA TAC AAG ACA TTA GAT TTA CGC AAG |
| *Oceanithermus profundus*  WP_013457601.1 | ATG GCT GAG GCG GAA ATT GCC CGC GGG TTG GAG GGC GTT GTA TTC ACC GAG ACT GAA TTA TCA TTC ATT GAC GGT CAG AAT GGG CGT CTG TAT TAC CTT GGT TAT CCT ATC CAA GAA TTG GCT GAG CAT TCA AGT TTC GAA GAA GTT TCT TAC TTA CTG TTA CAT AAA CGC TTG CCC ACG GCC GAC GAG TTG GCC GTT TTT AAG GAA AAA TTA GTT AAA AAT CGT AGT ATC GCG GTT GAG AAG GTA GCA AGC TTT GCT GAC TAT CCG CGT ACA GCG CAC CCC ATG GCG TCG TTG CGC ACC GCC ATC AGT GAG TTA GGA CTG TTT GAC CCA ACT GAA GAA GAT ACT TCA TTT GAA TCA TTG TAC GAG AAG TCA GTT TCA TTG ATT TCG AAG TTT GCC ACA GTG ACC GCG GCG ATC AAG CGT TTG CGC GAG GGT CAT ATG CCA ATC GAA CCC GAC CCC GAA TTA TCG CAC GCA GCG AAC TTC TAT TAC ATG TTA AAC GGC AAA CGC CCG AGT CCT GAA CAA GAG AAG TTG CTG GAT GTG GCT CTT ATC CTG CAT GCA GAA CAT GGA ATG AAT GCC TCC ACG TTT ACT GCG TTA GCC GTA CAT TCG ACG GCA AGT GAC ATT TAT TCG AGT ATC GTT GCC GCG GTT GGC GCG TTG AAG GGA CCG CGC CAC GGT GGT GCG AAC GAA CAG GTT ATG AAA ATG GTT CAA GAG ATT GGT AAA CCC GAA GCT GCT CGT GGT TGG GTG CAA GGG AAA TTA GCC AAT AAG GAG CGC ATT ATG GGC ATG GGC CAT CGT GTA TAC AAA GCT CTT GAT CCC CGT GCT GTG ATC TTG AAA AAA TAC GCG GAA ATT GTC GCC AAA GCT CAT GGA AAG AGT ACA GAA TAC GAA ATT TTG ACA ATT GTA GAA GAA GAA GCG GGA AAG GTT TTG AAC CCC CGC GGA ATT TAC CCC AAT GTT GAC TTC TAT AGC GGT GTT GTA TAT TCA GAC CTG GGA ATC CCG ACA GAA TTT TTC ACA CCC GTA TTC GCC GTG GCC CGT ATC TCT GGC TGG ACT GCA CAT ATC CTT GAG TAC GCT CGT ATG GAT AAT CGC CTG TTA CGT CCT AAG GCC AAA TTC GTA GGG GAG TTA GAC CGT AAA TAC GTT CCA ATT GAT CAA CGC |
| *Thermaerobacter marianensis*  WP_013495717.1 | ATG ACA CAA CCA AAT CCA GCC GGG CTG GAA GAT GTG GTC GCT GGA ACG TCG GAA ATC TGC TTT ATC GAC GGG AAA GAA GGT CGC CTG TTA TAC CGC GGC TAT GAT GTC CGC GAC TTA GCG GAA CAT GCA TCT TTC GAG GAA GTG GTT TAC CTG TTA TGG CAC GGA CGT CTT CCC AAT CGT GCC GAA CTT GAC GCC TTC GTG CAA CAA CTT CGC AGT TTG CAG GCT CTT CCG GGC CAG GTT CTT GAT TTG ATC CAG CAT ATC CCT CCT GCC ACA CAT CCC ATG GCT GCT TTG CGT ACG GCA GTA AGT TAT TTA GGT ACG TTG GAT CCC GAC CAA GAG GAC CAG TCT CAG GAG GCC AAT CTG CGC CGT GCG ACG CGC CTT GTC GCT CAA TTT CCG ACG CTT GTC GCC GCG ATT CAG CGC GTA CGT CAG GGT AAG GAG CCG GTC GCA CCG CGT CGC GAT CTG TCA TTA GCG GCA AAT TTT TTA TAC ATG CTT CGT GGA GAA GAG CCC TCC CCT CTG CAT GCG GAC GTT ATG AAC GTT GCA CTT GTG CTG CAC GCA GAT CAC GAA TTA AAC GCC TCT ACG TTT GCA GCG CGT GTT GTC GCG GCA ACG CTT TCT GAC ATG TAC AGC GCC ATT ACG GCT GCG ATT GGT GCC TTG AAA GGC CCC TTG CAC GGT GGA GCA AAT GAG CAG GTT ATG CGT ACT TTG TTA GAG ATC GGT TCA CCG GAT AAA GCT GAG GCC TGG GTT AAG GAA GCA TTA GCC GCA AAA CGT CGC ATC ATG GGC TTT GGG CAC CGC GTC TAC AAG ACT GAA GAC CCG CGT GCT ACG ATT CTG CGC CGT CTG TCC CGT AAA GTA GCA GAG GCT GCC GGG GAT CTT CGC TGG TTC GAA ATT AGC CAG GCC GTG GAA AAG GTT GTG ACC GCG GAG AAA GGC TTG TAT CCT AAT GTC GAT TTC TAC TCC GCC TCA ACT TAT TAC GTT ATG GGA ATC CCG TTC GAA CTT TAT ACC CCA ATC TTT GCG GTT TCC CGC ATC TCA GGT TGG ACA GCA CAT GTC TTG GAG CAG TAT GCA AAT AAC CGC TTA ATC CGT CCA CGC GCT GAG TAT ACT GGT CCG ACA CGT CGC GAA TGG GTG CCC TTG GAA CAA CGC |
| *Thermococcus chitonophagus*  WP_068576448.1 | ATG GTA AAC AGT GGT AAA GGG TTG GAA AAC GTC TAC ATT GAT CAA AGC TCA ATT TGT TAT ATT GAC GGT TTT GGG GGA AAG CTG TAT TAT CGC GGC TAT TCC ATT GAG GAA TTG GCC GAA ATG TCC AGC TTT GAG GAA GTA ACT TTC TTG TTG TGG TAC GGG GAG CTG CCT ACC AAG AAG GAA TTG GAA GAG TTT TCA AAA GAA CTG GCT CGT TCT CGT AAG TTA CCT GAG GAA TTA TTA GAC TTA TTG TAC CGT ATT CCA AAA ACC GCG CAC CCC ATG GGA GCA CTT CGT ACC GCG GTC AGC TTC TTA GGG AAT TTG AAC GGC GAA CCA TCA GCC ACA CCG GAA GAC GTG TTC CGC AAA GGG ATC TCG GTG ACC GCT AAG ATT CCC GTT ATC GTC GCT ACT TTA TAC CGT ATC AAG AAT GGT TTA GAT CCT ATC GAA CCG TCC ACG AAG CTT TCC CAC GCG GCA AAC TTT TTA TAC ATG CTT CAC GGC GAA AAG CCA CCT AAA GAG TGG GAG AAA GCA ATG GAC GTT GCT CTT ATT CTT TAC GCT GAG CAC GAG ATC AAC GCT AGC ACC TTG ACT GTG ATG ACA GTC GGC TCC ACG CTG TCC GAT TAT TAT AGC GCC ATT GTG GCC GGT ATT GGT GCT CTT AAG GGC CCC TTA CAT GGC GGA GCC GTG GAG GGA GCG ATT CGT CAA TTT ATG GAG ATC GGT TCC CCC GAG AAG GTA GAA GAG TGG TTC TTC AAT GCG TTG AAA GAA AAG CGT CGT ATC ATG GGT GCA GGG CAC CGT GTA TAT AAG ACT TAT GAT CCT CGT GCA AAG GTG TTC AAA AAA TAT GCA CGT CTT TTA GGA GAT AAG ACC TTA TAC GAG ATT GCC GAA CGT TTA GAG CGT CTG GTG CTG GAA CAT TTA GGA AAG AAA GGG GTG TCG ATT AAC GTC GAT TAC TGG TCT GGA ATT GTG TTT CAC GCG ATG GGT ATC CCC ATT GAG TTG TAT ACT ACC ATT TTT GCA ATG GGT CGT ATC GCT GGA TGG ACC GCT CAT TTG GCG GAG TAT ATC TCG CAC AAT CGT CTG ATC CGT CCC CGT CTT CAG TAT GTC GGC GAA ATT GGT AAA AAA TAC GTT CCG GTT GAA ATG CGC |
| *Thermus thermophilus*  WP_014510561.1 | ATG GAA GTC GCT CGC GGG CTT GAG GGG GTA TTA TTT ACC GAA TCG CGT ATG TGC TAT ATT GAT GGA CAG CAA GGG AAA TTA TAC TAT TAC GGA ATT CCA ATT CAA GAA TTG GCG GAA AAG TCG TCG TTT GAA GAG ACT ACC TTC TTA TTA TTG CAT GGC CGC TTG CCC CGT CGT CAA GAG TTA GAA GAA TTT TCA GCG GCA CTT GCC CGC CGC CGT GCA TTA CCT GCT CAC CTT TTA GAA TCG TTC AAA CGC TAT CCT GTA TCG GCC CAC CCT ATG AGC TTT CTG CGC ACA GCT GTA TCA GAA CTG GGT ATG CTG GAC CCT ACA GAA GGG GAT ATC TCA CGT GAG GCT CTT TAT GAA AAG GGT TTA GAC TTA ATC GCC AAG TTT GCG ACC ATC GTA GCT GCT AAC AAA CGC CTG AAA GAA GGG AAA GAA CCA ATC CCT CCT CGC GAG GAT CTG TCC CAC GCT GCG AAT TTC CTG TAT ATG GCT AAC GGA GTA GAG CCT TCC CCA GAG CAA GCT CGT CTG ATG GAT GCG GCG CTG ATC TTA CAC GCA GAG CAC GGG TTT AAC GCG TCT ACG TTC ACA GCA ATT GCC GCT TTT TCG ACT CAG ACT GAC TTG TAT TCA GCC ATC ACT GCT GCT GTA GCC TCG CTG AAA GGT CCA CGT CAC GGA GGC GCT AAT GAA GCG GTA ATG CGT ATG ATT CAA GAG ATT GGA ACT CCG GAG TGT GCC CGT GAA TGG GTG CGT GAG AAA TTG GCC AAA AAG GAA CGC ATT ATG GGC ATG GGC CAT CGT GTT TAT AAG GCT TTC GAC CCG CGC GCA GGT GTG TTG GAA AAA TTA GCC CGC CTT GTA GCA GAA AAA CAC GGG CAC TCG AAA GAA TAT CAA ATC CTT AAA ATC GTG GAG GAA GAG GCC GGG AAG GTA CTT AAC CCG CGC GGG ATT TAT CCA AAT GTG GAT TTC TAC AGC GGT GTG GTT TAC AGT GAC CTT GGC TTT AGC CTG GAG TTT TTC ACA CCA ATT TTT GCG GTG GCC CGT ATC TCA GGT TGG GTG GGA CAC ATC CTT GAG TAC CAA GAA TTA GAT AAT CGC CTT CTG CGT CCA GGC GCG AAA TAC ATT GGA GAA TTG GAT GTC CCC TAC GTC CCT CTT GAA GCG CGT GAT |
| *Ananas comosus*  XP_020089322.1 | ATG GAG GGT GCC TTC GAT CAT AGT GCA CTT GCC CGT GCG CGT TTA GCC GTG TTA TCA GGA CAC CTT GCA GCG GTG TCA GCC GCA GGC GGT GGC GCG TCA CCC TTA GAG CGC AGC CCA GTG AGT GTT CAA GAG ATT CCA CCA CCC CCT CGT AAT CTG GGC GGG TCA CTG GCG GTT ATT GAT GGG CGT TCA GGC AAG AAA TTT GAA TTC AAG ATC AGC GAC GAA GGC ACG GTT CGT GCT ACT GAC TTT AAA AAG ATC ACA ACC GGA AAA AAC GAC AAA GGG TTA AAA CTT TAT GAT CCG GGG TAC TTA AAC ACT GCA CCT GTT CGT TCT AGC ATC TGC TAC ATC GAC GGA GAC GAG GGT ATC CTG CGT TAC CGC GGC TAC CCG ATT GAA GAA TTA GCT GAG AGC AGC TCG TAT CCA GAG GTT GCC TAT CTT TTA ATG TAT GGA AAC TTG CCA TCG AAA AGC CAG TTG GCT GAT TGG GAG TTC GCC ATT AGT CAA CAC AGC GCT GTT CCT CAG GGT GTA CTT GAT ATC ATC CAA GGA ATG CCC CAT GAC GCA CAC CCG ATG GGG GTC TTA GTG TGT GCC ATG AGC GCG TTA TCT GTG TTC CAT CCT GAT GCT AAC CCG GCC TTG CGC GGT CAA GAT CTT TAC AAG AGT AAA CAG CTT CGC GAC AAA CAG ATT GTT CGT ATT TTG GGG AAG GTT CCG ACT ATT GCA GCC GCG GCT TAC TTA CGT TTA GCT GGC CGT CCC CCC GTT TTG CCT AGT AAT AAT TTC TCT TAT TCG GAG AAT TTC CTG TAC ATG CTG GAT AGT TTG GGG AAC CGC TCA TAC AAG CCA AAT ACG CGC CTG GCA CGC GTA CTT GAT ATT TTA TTT ATT CTT CAT GCC GAA CAT GAA ATG AAC TGC AGT ACT GCT GCT GCC CGC CAC TTA GCG TCC AGC GGC GTA GAT GTC TTT ACG GCG TTG GCC GGT GCC GTC GGG GCC CTT TAC GGC CCC CTG CAT GGG GGC GCT AAT GAG GCC GTA CTG AAG ATG CTT AAC GAA ATT GGC ACA GTA GAG AAC ATT CCG GAT TTC ATC GAA GGC GTA AAG AAT CGT AAG CGT AAG ATG TCA GGG TTT GGG CAT CGT GTT TAT AAA AAC TAC GAT CCT CGT GCG AAG GTA ATT CGT AAA TTA GCA GAA GAA GTT TTC TCA ATT GTT GGC CGT GAT CCT TTG ATC GAA GTA GCT ATC GCA TTG GAG AAA GCA GCT CTG TCT GAT GAA TAC TTC ATC AAG CGT AAA CTT TAC CCC AAC GTT GAT TTT TAC AGC GGT TTA ATC TAC CGT GCT ATG GGG TTC CCG ACT GAA TTT TTC CCT GTT CTG TTT GCA ATC CCG CGC ATG GCA GGT TAC TTA GCT CAC TGG CGC GAA TCG CTG GAT GAT CCA GAT ACC AAG ATC ATG CGT CCC CAG CAG GTC TAC ACG GGG GTT TGG TTG CGT CAC TAT ACC CCC TTA CAG GAA CGC ACC GTT AGT AAC GAT GAA GAC AAA CTT GGG CAG GTG GCG GTG AGT AAT GCC ACG CGC CGT CGT TTG GCG GGA AGT CGC ATT |
| *Cavenderia fasciculata*  XP_004355114.1 | ATG TCG CAC ATC TCT CGC CTT AAC GTT ATT TCT GGT CAT CTG AAG GAC GAG CAG GAA GAA GAG TAC GTG GAT GAA CTG ACG TCC AAC AAT GTG AAC GCC GAT GCT AAG AAG GAG ACG CTT ACG GTC ATT GAT AAT CGT ACC GGG AAG CAG TAC AAT ATC AAG ATC AAG AAC CAG ACT ATC TCC GCG TTG GCA TTC CGT GAA ATC TCG GAA CAG AAA GGG GAC AAT GGT TTA GTG GTC TAC GAC CCT GCG TTT CAG AAC ACG GCA GTT GTT ACT TCG ACA ATC ACC TAT ATC GAC GGC GAC AAG GGT ATC TTG CGT TAT CGC GGC TTC CCT ATT GAG GAG CTG GCA GAG CGT TCA TCG TTC TTA GAA GTT GCT TAT CTG CTG ATT AAC GGT AAT TTG CCG AAT AAA AGT CAA TTA GAT GGG TGG TCG CAC AAA ATT ATG ACG CAC ACC TTC TTA CAC GAG AAC TTG GTG GGG CTT ATG AAG AGC TTT CGT TAC GAC GCC CAC CCC ATG GGC ATG TTG ATC TCA ACG GTA GCC GCC TTA GGA ACT TTT TAC CCC GAG GCG AAC CCA GCG CTT GCA GGA CAG GAC ATC TTT AAG TCG GAA AAT GTA CGC AAC AAA CAA ATG TTC CGC ATT ATC GGG AAG TTG CCC ACA ATC GCG GCT TGT GCT TGG CGC CAC CGT ATT GGC CGT CCG TAC AAC ACG CCG GTG AAT CAC CTG GGC TAC ACC GAG AAC TTC TTG TAT ATG CTG GAT AAA CTT TCC GAA CAA GAC TAT AAG CCA AAT CCG GTA TTA TGC CGT GCC TTG GAA ATC TTG TTT ATT CTT CAC GCC GAC CAC GAA TTA AAT TGC TCT ACG GCG GCC ATG CGT CAT ATC AGT TCT TCC AAT ACT GAT CCG TAC ACG GCA GTC GCT GGC GCC GCA GGC GCT CTG TAT GGG CCC TTG CAC GGC GGG GCC AAC GAG GCC GTG TTA GAC ATG TTG CAA CAA ATT GGT ACC AAG GAA AAC GTT GGA CAA TTC GTG GCC GAC GTA AAA GCG AAA AAA AAG AAA TTG ATG GGG TTT GGA CAT CGC ATT TAT AAG AAC TAT GAC CCA CGC GCA AAG ATC GTC CGT CGC GTG GCC TAT GAA GTA TTC GAA TCT CTG GGT AAA GAA CCC CTG ATT GAA GTG GCT ACA GAG CTG GAA CGC CAA GCA CTT AAC GAC GAG TAT TTC GTA ACA CGT AAG TTA TAC CCA AAT GTC GAC TTC TAT TCA GGC TTA ATC TAC AAG GCC ATG GGA TTC CCG ACA GAC ATG TTC CCA GTG TTG TTT ACA ATC CCA CGC GCC GTA GGA TGG TTG GCC CAT TGG ATC GAG CAT CAC GAA GAT CCG GAG ACC AAA ATC TAT CGC CCA CGC CAA GTG TAT AAG GGA GAA TGG TTC CGC AAT TAC GTG CCA ATC GAG GGG CGC CCT CCA GCC AAA GTT CGT ACC CAA GAG AGC TAT AGT TCG GCC ACC ACT AAG CGT TAC TCG AAG GTC ACC GGC TCC GGC GCA CAA |
| *Chlamydomonas reinhardtii*  XP_001695571.1 | ATG ATG TCC AGC CGA CTG GAT GTT CTG TCT AGG CAG ATG TGC GGT ATG CAG CTC TCC CCC ACG GCC GGT GAA GAG GAG CAG CGG CCC GCG CCG GCC GGT GGT CGC GGA ACG CTG ACA GTT GTC GAC AAC CGC ACT GGC AAG AAG TAC ACG CTC GAG ATC TCC GAT GGC GGC ACC ATC AAC GCC CAG GCG CTA AAG CAG ATT AAG GCC GGT GGA GAC GGC GTG GGC CTG CGC ACC TAC GAC CCT GGG TAC ACA AAC ACC ACC GCC GTC ATC TCG CGC ATC TCC TTC ATC GAT GGC GAC AAG GGT ATT CTG CGC TAC CGT GGC TAC CCC ATT GAG GAG CTG GCT GCG CGA TCA AAC ATG ATG GAG GTG GCG TAC CTG GTT CTC TAT GGC AGC CTG CCC ACG CAG TCG CAG CTG TCG GTG TTC CAC GAG GCT GTG ATG CGG CAC ACG GCG CTT CCG ACG GAG GTG ATT GAT GTG ATT CAC GCC ATG CCG CAC GAC TCG CAC CCA ATG GGC GTG CTC ATG ACC GGC ATC TGC GCG CTG TCC GCC ATG CAC CCA GAG GCG AAC CCG GCT CTG GCG GGC CAG GGT GTG TAC AAG ACG CGC GAG ATG CAG GAC AAG CAG ATC GTG CGG CTG CTG GGC AAG ATC CCC ACC ATC GCC GCC ATG GCC TAC CAC AAA AGC ACG GGT CGC AAG GCC GCG CCG CCC AAC CAG CGG CTG GAC TAC ACG GAG AAC TTC CTC TAC ATG CTG GAC GGC GGC TAC AAC CCG CAG TAC CGG CCC AAC CCG CGC CTG GCG CGC GCG CTG GAC ATT ATG TTC CTC CTG CAC GCG GAG CAC GAG ATG AAC TGC TCC ACG GCC GCC GTG CGC CAC CTG GCC TCG TCG GGC GTG GAC GTG TAC ACG GCG GTG GCG GGT GCG GTG GGC GCG CTG TAC GGC CCG CTG CAC GGC GGA GCC AAC GAG GCG GTG CTG CGC ATG CTG GCG CGC ATT GGC TCG GTT GAG AAC ATC CCG GCC TTC ATC GCG GGC GTC AAG AAC AAG AAG GAG AAG CTG TTT GGC TTT GGC CAC CGA GTG TAT CGC AAC TTC GAC CCG CGC GCC AAG ATC ATT AAG GAC GTG GCG GAG AAC GAG GTG TTC CCG CTG GTG GGC GTG GAC CCG CTC ATC GAG ATC GCC AAG GCG CTG CAG GAC GCG GCC CTG TCT GAC GAG TAC TTT GTG AGC CGC AAG CTG TAC CCC AAC GTG GAC TTC TAC AGC GGC CTG GTC TAC CGC GCG CTG GGC TTC CCG CCG CAG TTC TTC ACC GTG CTG TTC GCC ATC CCA CGC ATT GTG GGC TAC TGC GCG CAC TGG CGC GAG TCG CTG ACG GAC CCG GAC ACC AAG ATC ATC CGG CCG CAG CAG GAC TAC AAG GGC GTG TGG CTG CGG CAC TAC TCG GAC GTG GTC GTG CGC ACC GCC GAG GGT GCG GAC ACG CTC TCG AAG CTG CCG CCC TCG AAC GCC TAC AAC CGT CGG GTT GCC GGT GAG AAC TGG ATG |
| *Chlorobium limicola*  WP_059139550.1 | ATG ACG GTA ACT GAG ACG GGT AAC TCG CTT ACC ATC GTA GAT AAC CGC ACC GGT AAG AGT TAC GAA GTT CCT GTG GAG AAT GGA AGT ATC AAT ACC ATG GAA CTG CGC AAG ATC AAG GTT TCG GAA GAA GAT TTC GGT TTA CTT GGG TAT GAC CCT GGA TAT TTG AAC ACA GCA TCA TGT AAG TCT CGT ATT ACC TAC ATT GAT GGG GAT AAA GGC ATC TTA CGC TAC CGT GGT TAC CCC ATC GAG CAA TTA GCG GAA AAG AGT ACT TTC TTA GAA ACT GCG TAC TTG CTT ATC AAA GGG GAA CTT CCG GAC AAG GAG CGT CTG GCT GTG TGG ACG TAT AAT ATC CGT CAC CAT ACC ATG ACC CAC AAT AAC ATC GTG AAA TTC ATG GAC GGG TTC CGC TAC GAC GCA CAC CCG ATG GGA ATC TTG GTC GGA ACT GTA GGT GCT CTG TCT ACT TTC TAC CGC GAC GCT AAA GAT ATC GGA AGC GAG GAT TCG CGC AAG CTG CAG GTG CGC CGC CTT ATT GGA AAG ATC CCC ACC TTA GCT GCA ATG TCT TTC CGC CAT AGC ATG GGG TTC CCT TAC GTT ATG CCT GAT AAC GAT CTG TCC TAC GCT GGG AAT TTC CTT TCA ATG ATG TTT AAG ATG ACT GAA CTT CGT TAT AAA CCG AAC CCA GTT TTG GAA CGT GCA TTG GAC GTG TTG TTT ATC CTT CAT GCG GAC CAC GAG CAG AAC TGT TCT ACG TCT TCC TTA CGC GCG GTC GCT AGC TCT GGC GTA GAT CCT TTT TCA GCG ATC GCC GCA GGC TGT GCT GCC TTA TAT GGG CCG CTT CAT GGC GGG GCC AAT GAG GCT GTT ATT CGC ATG TTA ATG AAG ATC GGT TCC ATT GAC AAG ATT CCA GAA TTT ATT CAA TCG GTA AAG GAC GGC GAT GGT CGC TTG ATG GGT TTC GGA CAT CGC GTA TAC AAG AAT TAT GAC CCT CGC GCT AAA ATC ATT AAA GAT ATC GCT TTC GAA GTG TTC GAG GAG ACT GGT CGC AAT CCG ATG CTT GAC ATT GCC TTA GAA TTA GAA CGT ATT GCT CTT GAG GAC GAC TAC TTT GTA AGC CGT AAA TTG TAC CCT AAC GTC GAT TTT TAT TCG GGA TTG ATC TAT CAG GCG ATG GGA TTT CCG ATG GAC ATG TTC CCT GTT CTT TTT GCC ATC GGC CGC ATC CCA GGG TGG CTG GCG CAG TGG ATC GAA CAC GTC AAA GAT GGC GAA CAA AAA ATC GCA CGT CCC CGT CAG ATC TAT CTT GGG GAG GAT GAG CGC CAA TTT GTA GCG ATG GCT GAC CGC CCA AAG ACC CGT TTG GAT GAA CAG ATG GCC GGA ATC TGC CGT TTG |
| *Conexibacter woesei*  WP_081691039.1 | ATG AGT GAG ACT CAG ACT ACG GGG GAC GGG GCG GTA GCA GCT GCA AAT GCG GAC ACC CTG ACA GTG ACG GAT AAT CGT ACC GGC CAG ACT TAC GAG GTA CCC ATC ACT GAC GGA ACT GTA CGC GCT ATG GAT TTC CGC CAG ATG AAA ACT TCA GAC GAC GAC TTT GGT CTG ATG ACC TAC GAT CCT GCA TTT ACG AAT ACA GCA TCC TGT CGT TCG GCG ATT ACC TAT CTT GAT GGA GAA AAC GGG GTA TTG GAA TAT CGT GGC TAC CCT ATC GAG CAA TTG GCC GAG CGC TCC ACA TAT CTG GAG GTG GCC TAC CTG TTA GTC CAC GGC GAG CTT CCA ACT ACT CCA CAG CTG GAT CAG TGG AAG CAC GAC ATT ACG ACT CAC ACA TTT GTG CAC GAG AAC GTT AAG GAG TTT GTA GGG GGG TTT CGT CAC GAC GCT CAC CCG ATG GGG ATG CTT GTC GGA AGT GTT GGA GCA CTG TCA ACG TTC TAC AAA GAC GCT AAC GAA ATT AGC GAC CCG GAA AAC CGC GCA CTG CAG ACA ATC CGT TTG ATC GCT AAG ATG CCG ACA TTG GCT GCT TTT GCC TAT CGT CAC ATT ATG GGA CAA CCA TAT TAC TAC CCT GAT AAT GAC TTA GAC TAT CCT GGA AAC TTC CTT TCG ATG ATG TAC AAG ATG ACA GAA CTG AAA TAC AAG CCA GAT CCG CGT TTG GAA CGC GCG CTT GAC GTT CTT TTT ATC TTA CAC GCA GAT CAC GAG CAG AAT TGT TCA ACC AAT GCT GTG CGT GCC GTA GGG TCG TCT CAA GTG GAC CCA TAC AGT GCC GTA GCG GCA GGA GTT GCC GCC CTG TAT GGG CCA TTG CAT GGA GGC GCA AAT GAG GCA GTT CTT CGT ATG TTG AAG CGC ATC GGA AAC AAG GAA AAC ATT CCT GAC TTT ATG CAA GGA GTC AAA GAT GGA AAT GAA CGC TTA ATG GGA TTC GGC CAC CGC GTG TAC AAA AAT TAC GAT CCC CGT GCA ACA ATT ATC AAA AAG GCA TGT GAC GAC GTA TTT GAG GTA ACA GGG GTA AAT CCT CTG TTA GAT ATC GCT CTG GAA GTA GAG AAA ATC GCG CTT GAG GAT GAG TAT TTT GTC TCC CGT AAG TTA TAC CCG AAC GTA GAC TTT TAC TCG GGC TTA ATC TAC GAA GCA CTG GGT TTG CCT ATG GAC ATG TTT CCT GTA ATG TTC GCG ATC CCT CGC ACA AGT GGA TGG ATT GCA CAG TGG TTA GAG ATG GTG CAA GAC AAA GAG CAA AAA ATT GCG CGC CCA CGC CAA ATC TAC ACT GGT GAA CGT ACC CGC GAC TAC GTC GGC ATT GAT GCC CGT AAG |
| *Corynebacterium pollutisoli*  WP_085550151.1 | ATG GCC ACC GAT AAC ACC GAA AAG GCG GTG TTG AAT TAT CCC GGC GGC GAA TTT GAA ATG GAC ATT ATC AAG GCG ACA GAA GGT AAC GAC GGT GTA GTG CTT GGG AAG ATG CTG GCG GAT ACC GGG TTG GTT ACA TAC GAC CCT GGA TAC GTT AGT ACA GGC TCG TGT GAA TCG GCG ATT ACA TAC ATC GAT GGG GAC AAT GGT ATC CTG CGT CAT CGT GGC TAT GAC ATC GCA GAC TTG GCC GAG AAT GCG ACG TTC AAC GAA GTC TCG TAT CTG CTT ATC AAC GGC GAA CTG CCT ACG GTA GAA GAG TTG CAT AAA TTC TCT GAC GAG ATC CGT CAT CAC ACC CTT CTG GAC GAA GAT TTT AAG TCT CAA TTC AAT GTG TTT CCA CGC GAC GCT CAT CCT ATG AGC GTG CTG GCG AGC TCG GTT AAC ATT TTG AGT ACT TAT TAC CAG GAT CAG CTT GAT CCG CTG AAC GAA GAG CAT CAG CAT AAA GCA ACA GTA CGT CTT ATG GCT AAG GTG CCA ATG CTT GCT GCC TAC GCG TAT CGC GCA TCA AAA GGG GCC CCC TAC ATG TAC CCA GAT AAC TCT CTT AAT GCC CGT GAA AAT TTC CTT CGC ATG ATG TTT GGG TAC CCA ACG GAG CCA TAT GAG ATT GAC GAG GTC ATG GTC AAG GCT TTG GAT AAA TTA CTT ATC CTG CAC GCC GAC CAC GAA CAG AAC TGC AGT ACT TCT ACT GTA CGC ATG ATT GGG AGC GCA CAA GCG AAC ATG TTC GTC TCT GTA GCC GGC GGA ATT AAC GCT TTG TCT GGG CCA CTG CAC GGT GGG GCG AAC CAG GCG GTG CTT GAG ATG CTG GAA GAA ATT GAT GCG AAT GGG GGC GAC GCA ACG GAT TTT ATG AAC CGT GTC AAA AAT AAA GAA AAG GGT GTT CGT CTT ATG GGA TTC GGA CAC CGC GTA TAC CGC AAT TAC GAC CCA CGT GCT GCC ATT GTG AAG GAG ACA GCG CAC GAA ATT TTG GAA CAT CTT GGT GGC GAT CAT CTG CTG GAT TTG GCC ATG AAA CTG GAA GAG ATC GCA CTT AAT GAC GAA TAC TTT ATT TCA CGT AAA TTG TAC CCG AAC GTG GAC TTC TAC ACA GGG CTT ATT TAC CGT GCA ATG GGG TTT CCA ACT GAC TTC TTT ACA GTC CTG TTC GCA ATT GGG CGT TTG CCT GGC TGG ATC GCC CAT TAC CGC GAG CAG TTG GCT ACT ACG ACC AAG ATC AAT CGT CCA CGT CAG ATT TAT ACG GGT AAC ACC TTA CGT ACA GTA ACA CCT CGT GAG GAA CGC |
| *Desulfoluna spongiiphila*  WP_092207573.1 | ATG TCA GAA GTA GTT AAA ATG ATT ATC GAA GGC AAG ACA TAC GAG TTT CCC GTA GTT GTG GGA AGC GAA GGA GAG AAA GCT TTT GAC ATC ACC AAG CTG CGT CAA CAA ACG GGG TAC ATT ACA ATG GAT CCC GGC TTT GGC AAT ACG GGC AGT TGT ACT TCG GCA ATC ACC TTC ATG GAC GGT GAG AAG GGG ATC CTG CGT TAT CGC GGA ATT CCG GTG GAG CAG CTT GCG GAG CAT TCG TCA TTC GTC GAG ACG GCG TAT CTT TTA ATC AAT GGA CGC CTT CCG AAT CGC CAG GAA CTT ACT CGC ACA AGT GTG CAG CTT AAT GAC GAC TCC TTG ATT CAT GAG GAT ATG CAA ATC TTC TAC CAA AAT TTT CCC CGC CGT GCG CAC CCT ATG GGG ATT TTG AGT GCC ATG CTG AAT GCT TTA CGT TCC TTC TAT CCT GAA TTA GAA GAC ACT GAC CCT GAA GGG ATC AAC CGT ACA TTC CTT CGT TTG TTA TCG AAG ATT CGT ACT ATG GCC GCG ATG TCT TAT CGC ATC AGC CGC GGT CAT AAA GTC GTT TAC CCC CGC GCG GAC TAC TCC TAT TGC GCT AAC TTC CTT AAT ATG ATG TTT GAT TCT CCG GTG CGT CCT TAC GAG TTG GAT CCT GAT ATT GTT GAG GCG TTA AAT GTG TTT TGG ATT CTT CAT GGG GAC CAT GAG CAA AAC TGC AGT ACC GCG GCC GTT CGT GTG GTG GGA TCT GGT CGC GTC AAT CTT TAC GCA GCC ATT TCA GCT GGC ATC GCC GCA CTT TGG GGC CCC CTG CAT GGG GGC GCA AAT CAG GCC GTA ATT GAG ATG TTG ACT AAT ATC CAT CAG TCC GGG GCG GAC ATT GAA GAT GTC GTT AAA CGT GCA AAG GAC AAG GAT GAT CCA TTT CGT CTT ATG GGA TTT GGA CAT CGT GTG TAT AAG ACA TAC GAT CCT CGT GCC AAG ATC ATG AAA AAA ATG TGC GAC ACT GTT TTG CCA AAA TTA AAA GTG GAA GAT CCT CTG TTA GAC ATC GCT CGT CGT TTG GAG GAG GTA GCG CTG ACC GAC CCA TAC TTC GTA GAC AAG AAT TTG TAC CCT AAT GTT GAC TTC TAT TCT GGA ATC GTG TTG CGT GCT ATG GGG ATC CCT ACT GAA ATG TTC ACC GTT ATG TTC GCT ATT GGT CGC TTG CCT GGT TGG ATC AGT CAG TGG AAG GAG GGA GCT GAT GAT CCG AAC TGG AAG ATT TCT CGT CCA CGC CAG GTG TAC ACC GGT AAT ACC GTA ACC GAT TAT TTA CCA ATG AAC CAG CGC |
| *Escherichia coli*  WP_166726827.1 | ATG GCT GAT ACA AAA GCA AAA CTC ACC CTC AAC GGG GAT ACA GCT GTT GAA CTG GAT GTG CTG AAA GGC ACG CTG GGT CAA GAT GTT ATT GAT ATC CGT ACT CTC GGT TCA AAA GGT GTG TTC ACC TTT GAC CCA GGC TTC ACT TCA ACC GCA TCC TGC GAA TCT AAA ATT ACT TTT ATT GAT GGT GAT GAA GGT ATT TTG CTG CAC CGC GGT TTC CCG ATC GAT CAG CTG GCG ACC GAT TCT AAC TAC CTG GAA GTT TGT TAC ATC CTG CTG AAT GGT GAA AAA CCG ACT CAG GAA CAG TAT GAC GAA TTT AAA ACT ACG GTG ACC CGT CAT ACC ATG ATC CAC GAG CAG ATT ACC CGT CTG TTC CAT GCT TTC CGT CGC GAC TCG CAT CCA ATG GCA GTC ATG TGT GGT ATT ACC GGC GCG CTG GCG GCG TTC TAT CAC GAC TCG CTG GAT GTT AAC AAT CCT CGT CAC CGT GAA ATT GCC GCG TTC CGC CTG CTG TCG AAA ATG CCG ACC ATG GCC GCG ATG TGT TAC AAG TAT TCC ATT GGT CAG CCA TTT GTT TAC CCG CGC AAC GAT CTC TCC TAC GCC GGT AAC TTC CTG AAT ATG ATG TTC TCC ACG CCG TGC GAA CCG TAT GAA GTT AAT CCG ATT CTG GAA CGT GCT ATG GAC CGT ATT CTG ATC CTG CAC GCT GAC CAT GAA CAG AAC GCC TCT ACC TCC ACC GTG CGT ACC GCT GGC TCT TCG GGT GCG AAC CCG TTT GCC TGT ATC GCA GCA GGT ATT GCT TCA CTG TGG GGA CCT GCG CAC GGC GGT GCT AAC GAA GCG GCG CTG AAA ATG CTG GAA GAA ATC AGC TCC GTT AAA CAC ATT CCG GAA TTT GTT CGT CGT GCG AAA GAC AAA AAT GAT TCT TTC CGC CTG ATG GGC TTC GGT CAC CGC GTG TAC AAA AAT TAC GAC CCG CGC GCC ACC GTA ATG CGT GAA ACC TGC CAT GAA GTG CTG AAA GAG CTG GGC ACG AAG GAT GAC CTG CTG GAA GTG GCT ATG GAG CTG GAA AAC ATC GCG CTG AAC GAC CCG TAC TTT ATC GAG AAG AAA CTG TAC CCG AAC GTC GAT TTC TAC TCT GGT ATC ATC CTG AAA GCG ATG GGT ATT CCG TCT TCC ATG TTC ACC GTC ATT TTC GCA ATG GCA CGT ACC GTT GGC TGG ATC GCC CAC TGG AGC GAA ATG CAC AGT GAC GGT ATG AAG ATT GCC CGT CCG CGT CAG CTG TAT ACA GGA TAT GAA AAA CGC GAC TTT AAA AGC GAT ATC AAG CGT TAA |
| *Fluviicola taffensis*  WP_0136874881­­­­.1 | ATG TCG AAG ATC GCA AAA ATC GAA TTG GAC GGC AAA GTT TAC GAA TTT CCA GTG GTT CAG GGA ACG GAA AAC GAG CTT GCA ATT GAC ATT AGT AAG TTG CGC CAA GAG ACA GGC TAC GTC ACT CTT GAC ACC GGC TAC AAG AAC ACC GGC GCC ACT ACA AGT GCA ATC ACC TTC CTT GAC GGG GAA GAG GGT ATT TTA CGT TAT CGT GGG TAT CCA ATT GAG CAA CTT GCA GAG AGC GCC ACC TTT ATT GAA GTG GCT TAC CTG TTA ATC TAT GGC GAA CTT CCA ACG CAG GTT CAA CTT GAC GAT TTT ATT TCT TCT ATT ACC AAG CAC ACC TTA GTC CAT GAG GAC ATG AAA CAG TTT TTC GAG GCT TAC CCA GCG CAG GCA CAT CCT ATG GGT GTA TTA AGT TCC ATG GTA TGT TCA TTG AGT ACG TTC TAC CCC GAG TCT TTA GAT CCT AAT CGC TCT GCG GAA AAA AAA AAT CGT ACG ATC CTG CGC CTT CTT GCC AAA CTT CCA ACT CTG GCA GCT TGG TCG TAT AAA AAC AGT ATG CGT CAT CCC TTC ATG TAT CCG AAA AAT GAA TAT GAT TAT GTG AAG AAC TTT CTT TAC ATG ATG TTC GCA ATG CCT ACG GAA GAT TAC AAG GTT GAT CCG GTA GTA GTC GAT GCC CTT AAT AAA CTG TTA ATT CTG CAC GCA GAC CAC GAG CAG AAC TGC AGC ACG AGT ACT GTT CGC ATC GTA GGC TCA TCT CAG GCG AAC CTG TAT GCG TCA ATC TCT GCG GGT ATT AGT GCC TTA TGG GGA CCA CTG CAT GGT GGG GCG AAT CAA GCG GTA ATC GAG ATG CTT GAG AAG ATC CAT AAC GAC GGT GGT GAC GTT GAC AAG TGG GTC CTG AAA GCC AAA GAC AAG GAT GAT CCG TTT CGC CTG ATG GGT TTT GGT CAC CGT GTT TAT AAA AAT TTT GAT CCG CGT GCC ACC ATT ATC AAG AAA GCT GCC GAC GAT GTC TTA GAG AAA TTG AAG ATC AAC GAC CCC TTG CTG GAT ATT GCC AAA AAA CTT GAA AAG TAC GCG CTT GAA GAC GAA TAC TTT AAG AGC CGC TCT CTT TAT CCA AAC GTC GAT TTC TAC TCC GGG ATC ATT TAC AAA GCT CTT GGT ATC CCG TCC GAA ATG TTT ACT GTC ATG TTC GCA CTG GGT CGC TTG CCA GGG TGG ATT GCA CAG TGG AAA GAA ATG AGT GAG GGT GGA GAA CCT ATT GGA CGT CCT CGC CAG ATC TAC ACA GGC GAG ACC ACC CGC GAA TAC GTC CCG ATC GCG AAA CGT GGT |
| *Methylobacillus flagellates*  WP_011478429.1 | ATG AAG CCC AGT AAG GTC ACG CTT ACG TTC GAT GAT GGT AGC GCC CCT ATC GAA CTG CCA ATT TTG CCT GGC AAA TTA GGT CCT AGT GTA ATT GAT ATT CGT TCA CTT TCA AAA CAC GGC TAC TTT ACT TAT GAT CCA GGT TTT CTT AGT ACA GCG TCG TGC GAC TCT AAC ATC ACC TTC ATT GAT GGC GAA GAG GGT CTT TTG TTC TAC CGT GGT TAC CCC ATC GAG CAA TTA GCT GAG CAC TCC GAT TTT ATG GAG GTC AGC TAC TTG TTG ATC TAT GGC GAG CTT CCG AAC GCG GAG CAA AAG GAG AAA TTC ACG AAT ACG ATC ACA CGT CAT TCT ATG GTC CAT GAC CAG CTG ACA CAC ATC TTC CGT GGG TTT CGC CGC GAT GCA CAC CCT ATG GCG GTC ATG GTG GGC GTC GTA GGT TCA ATG TCC GCT TTC TAC CAC GAA GCA ATT GAC GTT TCG GAT CCG CGC AAC CGT GAA TTT GCG GCC CAC CGC TTG CTT GCA AAG GTC CCT ACT ATC GCC GCA TGG TCG TAC AAA TAT AAC GTC GGC CAG CCT TTC ATG TAC CCC AAA AAC CGC TTT AAC TAC GCG GAA AAT TTC ATG CAC ATG ATG TTC GCC ACA CCT TGC GAG GAT TAT GAA CCG AAC CCC GTT CTT GCT AAA GCG TTC GAA CGT ATT TTG ATT CTG CAT GCA GAT CAT GAG CAG AAT GCA TCG ACA TCA ACC GTT CGT CTG GCG GGG TCA TCT GGC GCC AAC CCT TTT GCC TGC GTC GCA GCA GGA ATT GCG AGC CTT TGG GGT CCG GCT CAT GGC GGC GCA AAT GAA GCC ACT CTG AAG ATG CTT GAG GAG ATC GGC GAC ATT TCG CGC ATC GGT GAG TAT ATT AAG CGT GCC AAA GAC AAG ACT GAT AGC TTC CGT CTT ATG GGA TTC GGA CAC CGC GTA TAT CGT AAT ATG GAT CCC CGC GCT GCC ATC ATG CGC CAA ACC TGC CAC CAA GTG TTG GAC GAA CTT GGG TTG AAC GAC GAT CCG ATG TTT AAG CTG GCG TTG GAG TTA GAG AAA ATC GCG TTA GAG GAT GAA TAC TTC GTG TCC CGC CGT CTG TAT CCG AAT GTA GAT TTC TAT TCA GGT ATC GTG ATG CGC GCG ATG GGC ATC CCG AAC TCG ATG TTT ACG GCG ATC TTC GCA TTG GCG CGT ACG GTC GGA TGG GTG GCG CAG TGG AAT GAA ATG ATG TCG GAC CCT GGG TCG AAA ATT GGA CGC CCC CGC CAG TTA TAT ACT GGA CCT GAG CGC CGT GAT TTC CTG CCT CTG GAC CAA CGT |
| *Myxococcus xanthus*  WP_140862691.1 | ATG GCC AAG GAC ACG CTG ACG ATC ACC GAC AAT CGG ACC GGG AAG ACC TAC GAG GTC CCG ATC GAG AAC GGC TGT ATT CGC ACC AAT GCT CTC CGC CAG ATC AAA ACG GGC GAC GAT GAC TTT GGG TTG ATG GGC TAC GAT CCG GCG TTC CTC AAT ACC GCC AAC TGC AAG AGC GCC ATC ACC TTC ATC GAC GGA GAC AAG GGC ATC CTG GAG TAC CGG GGC TAC CCC ATC GAG CAG CTC GCG GAG AAG TCG AGC TTC CTC GAG GTC GCC TAC CTC CTG CTG AAC GGT GAG CTT CCC TCC GAG AAG GAG CTG CAG CAG TTC AAC CAC CTC GTC ACG CAC CAC ACC TAC GTG CAC GAG AAC GTG AAG AGC TTC ATG GAC GGG TTC CGC TAC GAC GCG CAC CCC ATG TCC ATG GTG AGC TCC ACG GTG GCG GCG CTG TCC AGC TTC TAC CCC GAC GCG AAG AAC ATC AAG GAC CCG CTG AGC CGC CGC ATC CAG ATC ACG CGG CTC ATC GCG AAG ATG CCC ACC ATC GCC GCG TTC TCC TAC CGG CAC ACG ATG GGC CTG CCG TAC ATC TAC CCG GAC AAC GAC CTG TCC TAC GTC GCC AAC TTC CTG GCG ATG ATC AAG CGC ATC GGC ACG TCG ACC TAC AAG GTC CAC CCG GTG CTC GAG CGC GCG CTC GAC ATC CTC TTC ATC CTG CAC GCG GAC CAC GAG CAG AAC TGC TCC ACC ACG TCC GTG CGC ACG GTG GGC TCG TCC GAG GTG GAT CCG TAC TCG GCG GTC AGC GCG GGC ATC GGC GCC CTC TAC GGC CCG CTG CAC GGC GGC GCC AAC GAG GCG GTG CTC CGC ATG CTC CGC GAC ATC GGC CAC GTC TCC AAG ATC CCG GAG TTC ATC AAG TCG GTG AAG AGC GGC GAG GGT GAG AAG AAG CTG ATG GGC TTC GGT CAC CGC GTC TAC AAG TCC TAC GAT CCG CGC GCC AAG GTC ATC AAG CGC GTG GCC GAC GAG GTC TTC GAG GTG ACG GGC AAG AAC CCG CTG CTG GAG ATC GCC GTC GAG CTG GAG CGC ATC GCG CTC GAG GAC GAG TAC TTC GTG AAG CGG AAG CTG TAC CCG AAC GTC GAC TTC TAC TCG GGC CTC ATC TAC GAG GCG ATG GGC TTC CAG GTC GAG ATG TTC CCG GTG CTC TTC GCC ATC CCC CGC ACG GTG GGC TGG TGC GCG CAG TGG GAG GAG ATG GTG ACG GAC AAC GAG CAG AAG ATT GCC CGT CCG CGC CAG GTG TTC ACG GGC GCC CCG CGC CGC GAC TAC GTC CCC ATG GAC AAG CGC GCC GTC AAG |
| *Neisseria arctica*  WP_047760537.1 | ATG AGT AAG ACT ATT AAA TTA CAA GTC GAA GGA CAA GAG GCG TTC GAA CTG CCC GTT TTG GAG GGT ACG TTA GGT AAC GAT GTA GTG GAC ATT CGT ACT TTT ACT AAA GAG ACC GGT ATG TTT ACT TTC GAT CCG GGG TTT GTT TCG ACC GGG TCC TGT GAG TCG AAG ATT ACC TTT ATC GAT GGT GAG AAG GGC CAA TTA TAC TAC CGT GGC TAC CCT ATC GAA CAG TTG GCT GAA AAT AGC GAC TAC CTT GAA ACC TGC TAC CTG CTT ATC TTC GGG GAG CTT CCC ACC GCT GAA CAG AAA GCA CAA TTT GAA CAC ACT GTG TCG CGC CAC ACG ATG GTC CAC GAA CAA CTT ACT TGG TTC TTC CGC GGA TTC CGT CGC GAC GCT CAC CCG ATG GCA ATG ATG GTC GGT GTG GTA GGC GCA CTT GCA GCT TTT TAT CAA GAT AGC TTG GAC ATT AAC AAC GCT GAC CAT CGT CGC ATT GCT GAG TTT CGC CTT ATT TCT AAA ATT CCG ACA ATC GCG GCC ATG TGT TAC CGC TAC TCA AAC GGA CTT CCG TTC AAC TAT CCT CGC AAC GAT TTA TCT TAT ACG GCA AAC TTT ATG CAC ATG ATG TTC GCT ACA CCG TGC GAG GAG TAT CAG CCC AAC CCC GTT CTG GTG CGC GCA CTT GAC CGC ATT TTT ATC TTA CAC GCA GAT CAC GAA CAA AAC GCA TCT ACT TCC ACA GTG CGT CTG GCA GGA AGC TCG GGA GCG AAT CCG TTT GCA TGC ATC GCG GCG GGT ATT GCA AGC TTG TGG GGT CCC GCT CAC GGT GGT GCA AAC GAG GCG GTG CTG AAA ATG CTG GAC GAA ATC GGC GAT GTG AGT CGT GTG CCG GAA TTT ATG GAA GGT GTG AAG GAG CGC AAG TAC CGT TTA ATG GGC TTC GGT CAC CGC GTC TAC CGC AAC ATG GAC CCG CGT GCT AAC ATT ATG CGC GAG ACT TGC TAT GAA GTA CTG AAG GAA TTG GGG CTG GAA AAT GAC CCC AAA TTC AAG CTT GCC ATG GAA TTG GAG CAG ATC GCG TTA AAC GAT CCG TAC TTC GTG GAG CGT AAG TTA TAT CCT AAC GTT GAT TTC TAT AGC GGG ATT GTA CTT TCT GCC CTT GGG ATT CCT GTC TCA ATG TTT ACA GTC ATT TTT GCT TTA GCA CGT ACC GTT GGC TGG ATT AGT CAC TGG CAC GAA ATG ATT GCT GAC CCT GGT CAT AAG ATT TCT CGT CCT CGT CAA TTG TAT ACG GGG GCT GTA CGT CGT GAT TAT GTT CCA GTC GAC AAA CGC |
| *Patulibacter americanus*  WP_022926837.1 | ATG AGC GAG ACA CAA GAG CGC GGT GGC GAC GCG GCG GTC GCA ACA GAT CAG CAA ACG CTG ACT GTC GTC GAC AAT CGT ACC GGC AAG CAG TAC GAA GTC CCC ATC AGT GAT GGA ACT GTC CGT GCA ATG GAC TTT CGC CAG ATT AAA ACT GGA GAC GAC GAC TTT GGA TTG ATG ACG TAC GAC CCG GCA TAT ACA AAT ACG GCA AGC TGC CGT TCA TCG ATC ACC TTT ATC GAC GGC GAT AAA GGC ATC TTA GAA TAT CGT GGC TAC CCG ATT GAG GAA TTG GCT GAG AAG GCA ACT TTC CTG GAG GTA GCT TAC CTG TTG ATT TTT GGT GAG TTG CCT ACG ACT GAT GAA TTA GCA GAG TGG ACG CAC GCT ATT ACG ACC CAC ACA TTT GTG CAT GAA AAC GTA AAA AGC TTC GTA GAA GGA TTT CGT TAT GAT GCA CAT CCG ATG GGA ATG TTG CTT GCA TCG GTA GGG GCG CTT TCG ACC TTC TAT CCG GAA GCA AAA GAG ATT AAA GAT GAA CAA TTA CGC TAC GAA CAA ATC GTC CGT CTT ATC GCC AAG GTT CCT ACT CTG GCG GCC TTT GCG TAC CGC CAC GCC AAG GGC ATG CCT TAC GTA TAC CCG GAC AAC GAC TTA AAT TAT GCC GAA AAC TTT CTG GGT ATG TTG TTC AAA ATT GCA GAA CTT AAA TAT CAG GCT GAC CCT CGT ATT GCT AAA GCC CTG GAC GTG CTG CTG ATC TTA CAC GCA GAC CAC GAA CAG AAC TGC AGT ACC TCG TCA GTA CGT GCA GTA GGG TCA AGC CAA GTC GAT CCA TAT AGT GCA GTA ACC GCC GGA ATC GCA GCC CTG TAT GGT CCC CTT CAC GGG GGA GCG AAT CAA GCG GTT TTA GAA ATG CTT CAG GAG ATT GGC ACA GTC GAA AAT GTA CCT GCC TAT ATC GAG AGT GTC AAA TCC GGG GAT AAA AAG TTG ATG GGC TTT GGC CAC CGT GTG TAT AAA AAT TTC GAT CCG CGT GCC AAA ATT ATT AAG AAG GCC GTT GAT GAT GTA TTT GAG GTT ACT GGA GTG TCT CCT CTT TTG GAG GTT GCC CAG GAA TTG GAA CGT ATT GCC TTG GAG GAC GAC TAC TTT GTA AAA CGC AAA CTT TAC CCG AAC GTC GAT TTC TAC TCT GGA CTT ATC TAT GAG GCC CTT GGC CTT CCC GTG GAA ATG TTC ACA GTT TTG TTC GCA ATC GGC CGC ACG CCT GGG TGG ATT GCG CAG TGG CTT GAG ATG GTG CAA GAT AAG GAG CAA AAA ATC GCC CGC CCT CGT CAG ATT TAT ACC GGG GAA CGT ACC CGT AGT TAC GTT CCT ATT GAT GAT CGT TCT |
| *Perkinsus olseni*  AND95703.1 | ATG GAC CGC TTG AAT ACC GTA GCT GCC CAT GTC GCA CCA ACT CTT ACA ATT ACG GAT AAT CGC AGC GGA AAG AAG ATC GAC GTG AAA GTT AAA GAT GGG ACG ATC CGC GCA ACG GAT CTT AAA CAG CTT GGT ATT CAA ACA TAC GAC CCG GGC TAT ATG AAC ACT ACC TGT TGC GTA TCA CGC ATC TCT TTT ATT GAT GGG AAC CGT GGA GTG CTT CGT TAC CGC GGC TAT CCT ATT GAA CAA TTA GCC CAG GAT AGC AGT TTC ACT GAG GTT GCC TTT CTG TTG CAG TAT GGA GAA CTG CCC ACG CGT GGC CAA TTA ACG CAG TGG GAG CAA GAC CTG ATG CGT CAC AGT ATG CTG CAT CAA GAT GTA GCC ACG CTT ACG TCC GCG TTC CGT TAC GAC GCG CAT CCG ATG GGC ATG TTC GTA TCA GCC ATG GCT GCT CTG GGA ACG TTG CAC CCC GAG ACC AAC CCA GCA TTA ACT AGC CAA AAC ATT TAT AAG GAT AAG GCG GTT CGT AAC AAA GAA ATT GCC CGC ATC TTG GGT TCA GCT ACA ACT TTA GCT GCT ATG GCA TAC CGT CAT CGC ATG GGC CGC CCT TTT AAC ACC CCA AAC TCC GAT CTT GGT TAT ACT GAA AAT TTC CTT TAT ATG TTA GAT GTG TCC CAC GAA GGC ACG TCT TAT CGT CCG CAC CCC AAA CTG GTA AAA GCT TTG GAC GTG CTT TTC GTC CTG CAC GCC GAG CAC GAA CTT AAC TGT TCG ACA AGC GCC ATG CGT CAT TTA ACT AGT TCA AAT GTC GAT GTT TAT ACA GCA TGT GCC GCT GCG GCC GGT GCG TTG TAC GGA CCC CGT CAT GGG GGA GCG AAT GAA GCG GTC TTA CGC ATG TTG GAG CGC ATT GGG TCA ATT GAG AAC GTA CCT ACC TTC GTA GAT AAG GTC AAA CAG CGT AAA GAG TTG TTA ATG GGC TTC GGA CAT CGC GTT TAT AAG CAT TAT GAT CCT CGC GCG ACC ATC GTA CGC AAA ATC GCA GAG GAT GTC TTC GAG ATT TGC GGT CGC GAA CCG CTT ATT GAG GTA GCC ATG GAT TTA GAA CGC ATC GCA TTG TCG GAT CCG TAC TTT AAA GAT CGC AAG CTT TAC CCT AAC GTA GAT TTT TAC TCA GGC CTG ATT TAT AAG GCC ATG GGT TTT CCG ACT GAT ATG TTC CCA ATC TTA TTT ACC GTT CCT CGT CTG GCA GGC TGG CTG GCC CAT TGG TCC GAA TGG ATC GAC GAT CCT GAG AAC CGT ATC TAC CGC CCC TTC CAG GTT TAC AAG GGC TAT GAT AAG CGC GAT TAC GTG CAA GTT GAT GAA CGT AGC GAG AAA GTT CAC TCT AAG GAA TTG ACT GTA AAG CGT AGT GCC TTT AAC CGT CGC CGC GAC GCG TCG CTT CAG TCA GCA GCC GCG GAC CGC TCG GAA TAC GAG TGG ATC AAA GAT |
| *Perlucidibaca aquatica*  WP_068858516.1 | ATG ACA GCG AAA ACC GCC ACA CTT ACC ATT GGC GAC GAA ACT TTA ACA CTT CCC ATC ACG AGC GGT ACA CTG GGA CCA GAC GTC ATT GAT GTT AAA GAT GTC CTG GCT AAA GGT TAT TTT ACG TAC GAT CCG GGG TTT ATG GCA ACG AGT GCG TGT GAA TCA AAG ATT ACA TTC ATC GAC GGA GAT AAA GGG ATC TTG CTG CAC CGT GGT TAC CCT ATC GAT CAG TTA GCG GAG AAA GCG GAA TAT CTG GAG GTA TGT TAT CTT CTT TTG AAC GGT GAG CTT CCA AAC GCG CAG CAA AAA GCC GAG TTC ACA AAA CTT GTT ACA GAT CAT ACG ATG GTC AAC GAT CAA CTG CGT AAG TTT TTC GAA GGG TTT CGC CGC GAC GCT CAC CCG ATG GCT GTG ATG TGT GGC GTG GTG GGG GCG CTG AGC GCA TTT TAT CAC GAC GGT TTA GAC GTA AAT GAT CCT AAG CAT CGT GAG ATC ACA GCG ATC CGT CTG ATC GCA AAA ATC CCT ACC ATT GCT GCA ATG TCC TAC AAG TAT ACA GCA GGA CAG CCT TTT ACC TAC CCT CGT AAC GAC CTG AAC TAC GCG GAG AAT TTT TTG CAC ATG ATG TTC TCC GTG CCA GCG GAC GTC AAC TAT AAG GTC AAC CCG ATT CTT GCG AAG GCT ATG GAT CGC ATT TTC ACC CTG CAT GCA GAC CAT GAA CAA AAT GCG TCT ACA TCA ACG GTT CGC CTT GCT GGC TCA ACG GGA GCG AAT CCG TAT GCA TGC ATC GCC GCC GGT ATT GCC GCG TTA TGG GGC CCC GCC CAC GGA GGA GCG AAC GAA GCC GTA TTG AAG ATG TTA GAT GAA ATT GGT AGC GTA GAG AAT GTC GCG GAT TTC ATG GAA AAA GTG AAA ACA AAA GAG GTG AAG TTG ATG GGC TTT GGC CAC CGT GTA TAC AAG AAT TTT GAT CCT CGT GCT AAA GTA ATG AAA CAA ACG TGT GAT GAA GTG TTG GCC GCA TTG AAC ATC AAC GAT CCT CAA TTA GCG TTG GCT ATG GAG TTA GAA CGT ATC GCT CTG AAC GAT CCA TAT TTT GTA GAA CGC AAA CTG TAC CCG AAC GTT GAT TTT TAC TCA GGG ATT ATC TTA AAA GCG ATT GGA ATT CCA ACA TCA ATG TTC ACC GTC ATT TTT GCT CTT GCC CGC ACA GTG GGA TGG ATT TCA CAC TGG CTT GAA ATG CAT TCC GGG CCG TAC AAG ATT GGG CGC CCC CGT CAG TTG TAC ACG GGG TAC GTG CAA CGC GAC CTT CCA CCG GTA GAG AAA CGT |
| *Roseomonas rosea*  WP_073132632.1 | ATG TCA GAT GCT TCG ACG CCG GGA TCG GTC ACG ATT ACG CTT GAC GGC ACC AAT AAA TCC TCG CGT GCC CCC TTG GTA CAG GCA TCC GTT GGC CCC GCA GTA GCA GAT ATT CGC AAG TTA TAC GCT GAC CTT GGC GTA TTT ACT TTC GAC CCG GGT TTC GGT ATG ACC GCT GCC TGC GAA TCA AAG ATC ACA TAT ATT GAC GGA GAT AAG GGA GTA CTG CTG TAT CGC GGG TAC CCC ATC GAA CAG CTT GCG GAA AAT AGC GAT TTT ACT GAA GTG TGT TAC CTG TTA CTG CAC GGT GAG CTT CCA AAC GAT GCG CAG TTA AAG GAA TTT AGC CAT AAT GTT ACA ATG CAC ACT ATG GTC CAT GAA CAA ATT CGC AAT TTC TTC AAT GGG TTT CGC CGC GAT GCT CAC CCG ATG GCG ATT TTG TGC GGC GTC GTC GGA GCC TTG TCT GCG TTC TAC CAT GAC TCA TTG GAT ATC TCT GAC CCG CGT CAA CGC GAA ATT GCG GCC TTT CGT CTT ATT GCC AAA GTA CCG ACG ATC GCT GCT ATG GCG TAC AAG TAT TCA ATC GGC CAA CCT TTC GTG TAC CCA CGC AAT GAC CTT TCA TAC GCC GAA AAC TTC TTA TAT ATG CTG AAC GCC GTT CCG GCG GAG GAA TAC AAG GTG AAC CCC ATT CTG GCA CGT GCG ATG GAT CGT ATT CTG GTC TTA CAT GCC GAT CAT GAG CAG AAC GCT AGC ACT AGC ACG GTA CGT CTG GCT GGC AGC ACA GGT GCA AAT CCG TAT GCC TGC ATC GCA GCG GGT ATC GCG GCT CTT TGG GGA CCA GCG CAT GGG GGA GCC AAT GAG GCG GTT CTT AAG ATG TTA GGG GAA ATT AAA ACT CCC GAA AAC ATC CCT GAC TTC ATC GAG AAG GTG AAG GAT AAG AAC TCA TCC GTG AAA CTT ATG GGG TTT GGT CAT CGT GTA TAC AAG AAT TTC GAC CCA CGC GCT AAA ATC ATG AAG GAA ACA TGT CAC GAA GTA TTG GCA GAG CTG GGC ATC AAG GAT GAA CCT CTG CTT GAC ATG GCT ATG GAG ATG GAA CGT ATT GCT CTG TCC GAT GAC TAC TTT GTA AGC CGT AAG CTG TAT CCT AAC GTG GAC TTC TAC TCC GGG ATT ATC CTT AAA GCT ATG GGA ATC CCC ACA TAC ATG TTC ACT GTT CTT TTT GCC GTT GCC CGC ACG GTA GGC TGG GTT TCG CAA TGG AAA GAA ATG ATC GAA GAG CCG GGG CAA CGT ATC GGA CGC CCG CGT CAG GTG TAC ACA GGA GAA ACT AAC CGC GAT TAC GTG CCG ATG GGA AGT CGC GGG |
| *Sorangium cellulosum*  KYF62269.1 | ATG AAA AAG AAT GGA GCG GAG CAG ACT ATT GAG CTT CCT GTT ATT ACA GGG ACA GAG GGA GAG AAA GCT ATT GAC GTA GCT TCC CTG CGT TCG AAA ACC GGC TAC GTG TGC ATC GAT CCC GCC TTC GTC AAT ACT GCG AGC ACG ACA TCA TCG ATT ACA TTT TTA GAT GGT GAG AAA GGG ATT TTG CGC TAT CGC GGT ATC CCG ATC GAA CAA CTG GGG GAA AAA TCT ACG TTT GTA GAA ACT TCA TAC TTG TTA ATC TAT GGC AAG TTG CCA AAT AAG AGC GAA CTT TCC CGT TTC TCG ACG CTG CTT ACT CGT CAT AGT CTT ATC CAC GAG GAT ATG AAG CGC TTC TTC GAT GGG TAT CCG TCC ACG GCT CAC CCT ATG GCA ATC TTA TCC GCT ATG GTC CTG AGT TTG TCG AGC TAT TAT CCA GAA GCG ATT GAT GTC AAG AAT ACG GAT TTT TTG GAT ATT ACG ATC GCT CGC TTA CTT TCC AAA GTT CGT ACT ATC GCC GCT TTC TCG TAT AAA AAG TCG ATT GGA CAG CCC TTT GTC TAC CCA CAT AAC TCA CTG TCA TAT TGT GCA AAT TTC TTG AAC ATG ATG TTC TCA GTT CCC GCC GAG CCT TAT GAG ATT GAT GAA GAC ATC GTC AAG GTG CTT AAC CTG TTG CTG ATT TTA CAT GCT GAT CAC GAA CAG AAT TGC TCC ACA TCC ACT GTT CGC CTG GTA GGT AGC GCA AAA ACC AAC TTA TTC GCG TCC ATC GCT GCC GGC ATC TGT GCG CTT TGG GGA CCC TTA CAC GGC GGT GCA AAC CAG GAG GTA GTC ACA ATG TTA GAG GCA ATT CGC GCC GAT GGT GGT GAC GTT AGT AAG TTT GTA AAG CTG GCA AAA GAC AAG ACA TCC GGT TTT CGT TTG ATG GGT TTC GGC CAT CGT GTA TAT AAA AAC TAT GAC CCA CGC GCG GTT TTG ATT AAG GCA GCA GCG GAT AAG ATT CTG AGC AAA TTA GGG ATT AAA GAT CCC CTT CTT GAT ATC GCG CAA CGT CTG GAG GAG ACT GCT CTT AAG GAT CCT TAT TTC GTA GAA CGC AAG TTA TAC CCC AAC GTA GAC TTC TAT TCC GGT ATC ATC TAT CGC GCG CTT GGC TTC CCA ACG AAT ATG TTT ACG GTT ATG TTC GCT TTA GGT CGC CTG CCC GGC TGG ATT GCG CAC TGG AAA GAA ATG AAT GAT GAT CCC AGC GGT AAA ATC GGG CGC CCA CGT CAG ATT TAC ACG GGG GAA AAT GCA ACA GAT TAT ACT CCG TTA GAC GAC CGT GCG |
| *Thioclava pacifica*  WP_038072739.1 | ATG GCA GAG AAC CAG AAA ACC GCA ACG CTG TCC TTG GAT GGT ACC ACT TAC GAG TTG CCC GTA TTG AGT CCC TCA GCG GGC CCA GAT GTT CTT GAT ATC CGC AAG CTG TAC GGG CAA GCT GAC GTT TTC ACA TAT GAT CCG GGA TTC ACG TCT ACA GCC TCT TGC GAA AGC AAG ATC ACC TAT ATC GAT GGC GAT AAG GGG GAA TTG CTG TAC CGC GGG TAC CCG ATT GAC CAA TTG GCT GAG AAA TCC CAT TAC CTG GAG GTA TGT TAC CTT CTT CTT TAC GGG GAA TTA CCA ACT CGC GCC CAA ATG GAG GAC TTC GAG TAC CGC ATC ACG CGC CAT ACA ATG ATC CAC GAG CAG ATT CAT AAT TTC TTC CGC GGG TTT CGC CGT GAT TCG CAT CCC ATG GCA ACC ATG GTT GGA GTA GTG GGC GCA ATG TCG GCA TTC TAT CAC GAT AGT TTA GAT ATC AAC GAT CCG TGG CAG CGT GAA GTA GCT TCA ATG CGC TTA ATC GCC AAA CTG CCG ACG ATC GCG GCT ATG GCG TAC AAA TAT TCG ATC GGA CAA CCC TTT GTT TAT CCG CGC AAC GAC CTT AGT TAT GCC GAA AAT TTT CTG AAT ATG TGT TTC TCG GTA CCA GCA GAA CCC TAT AAG GTG GAA CCG GCG CTG GCT AAG GCT ATG GAC CGC ATC TTT ACG TTA CAT GCT GAC CAT GAA CAG AAC GCA TCA ACA AGT ACC GTG CGT CTT GCT GGT TCC AGT GGG GCA AAC CCG TTT GCC TGT ATT GCG GCG GGA ATC GCT TGT TTA TGG GGG CCA GCA CAC GGT GGA GCA AAT CAG GCA TGT CTT GAA ATG CTG CGT GAA ATC GGA TCT GTC GAC AAG ATT CCT GAG TAT ATC AAG CGC GCA AAA GAC AAA GAC GAT CCA TTT CGC CTG ATG GGA TTC GGT CAC CGC GTG TAC AAA AAT TTC GAT CCT CGT GCT AAA GTA ATG AAA GAA TCA GCG GAT GAG GTA TTA GAT TTA TTA GGG ATT CAC GAT AAC CCA ACC CTT CAG GTT GCC AAG GAG TTA GAG AAG ATT GCT CTT GAA GAC GAA TAT TTC GTA TCT AAG AAG CTT TAC CCT AAT GTA GAT TTT TAC TCC GGC ATC ATT CTT GAA GCA ATG GGC TTC CCC ACT TCG ATG TTC ACG CCT ATT TTT GCA CTT TCC CGC ACT GTA GGT TGG ATC TCG CAG TGG AAA GAA ATG ATC GAA GAC CCC ACG AAT AAG ATT GGG CGC CCC CGC CAG CTT TAT ACA GGT GCT ACA TTC CGC GAC TAC GTG GAT GTT GAA AAG CGT |
| *Nitrospira inopinata*  WP_062485514.1 | ATG ATG CCC CAC GAT TTT ATG CCA GGT CTT GCA GGG GTG CCA GCG GCA ACT AGT TCA ATC TCC GAC GTT GAC GGC CAG CGT GGA GTT TTG GAA TAC CGT GGG ATT CGT GTA GAG GAG CTG TGC GCC AAG TCG TCA TAT TTG GAA ACA GCA TAT TTA TTA CTG TTC GGG CGT CTT CCT ACT CGC GCA GAA CTT CAC CGT TGG ACC GCG GAC GTA ACC CAC CAT CGT CGC GTA AAA TTC CGT ATC GTA GAC TTG TTG AAG TGC TTA CCC GAG CAG GGC CAT CCC ATG GAC GCT CTG CAA GCC GCG GTC GCG GCC CTT GGG ATG TTT TAC CCT GGG CGC AAC GTC AAA GAT GCT GAC AAC AAT TAC TGG TCG GCC GTC CGT CTT GTG GCG AAG TTA CCC ACT ATT GTC GCG GCA TGG GCT CGT ATT CGC CGT GGT GAC GAC CCC ATT CCA CCA CGT GAT GAC TTG GGA TTT TCG GAG AAT TTT CTG TAT ATG CTT ACA GAA ACT GCG CCC CCG CCA TTA TGG AGT GAA GTC TTT GAC GAC TGT CTT ATT TTA CAC GCT GAA CAT ACA ATG AAT GCT AGC ACT TTC GCA GGG CTG GTA ACT GCA AGC ACA TTG GCA GAT CCA TAT ACG GTT GTG GCG TCA TCT ATT GGC GCA CTG AAA GGC CCA CTG CAC GGA GGT GCC AAT GAG GAA GTA GTT CAG ATG TTA ATT GAG ATT GGG TCT CCA GAT CGT GCT CGT ACG TAT GTT GAA GAA AAG CTG CGT GCT AAG CAA AAG TTA ATG GGC TTT GGT CAC CGC GTT TAC AAG GTA AAA GAC CCT CGC GCA ACG GTA CTT CAA GAA CTG TGC CGT CGC TTA TTT ACG GAG AGT GGA CCG TCA CCC TTT TAC AAT ATC GCG TTA GAG GTT GAG CGT GCA GCG GGA GAA CGT TTG GGA GAT AAA GGG GTT TAT CCC AAC GTG GAT TTT TAC AGT GGT ATT ATC TAC GAC AAG ATG GGG ATC GAG ATG GAT CTT TTT ACT CCT ATT TTC GCG ATG GCC CGC GTC GCT GGA TGG CTG GCT CAT TGG CTT GAA CAA CTT GGT GAA AAT AAA CTT TTT CGC CCA GAC CAG ATC TAC TCA GGA GAA CAT AAC CGC CCG TAC GTT CCG ATC GAC CAG CGC |
| *Cyanobium sp.* PCC7001  WP_006910478.1 | ATG GCC GGG AGC TTG AGT GAT AGT GTA CCT GGA TCG ACT GGA GGG GCA ACG GCT GCC CCA CCA TTT CGT CCC GGG TTG GAA GGT GTA CCA GCA ACT CAG AGC GCG ATC TGT GAC ATT GAT GGA CAA AAG GGT CGT TTA ACA TAC CGC GGG TAC GAC GCG GGG GAG CTG GCC GCT CAT AGC ACG TTC TTG GAA ACA ACC TAC TTA TTA ATT TGG GGA GAG CTT CCC ACC GCC GAA GGT TTG CGT CAG TTT GAA CAC GAG GTT CAG ATG CAT CGC CGT GTT TCC TTC CGT ATT CGT GAC ATG ATG AAA TGC TTC CCC GCT ACA GGC CAT CCC ATG GAT GCA TTG CAG TCG TCC GCC GCC AGC TTG GGT CTG TTT TAT TCC CGC CGT GCG CTG GAC AAT CCC GAG TAT ATT GCA GAA GCG GTG GTA CGC TTA ATT GCC AAA ATT CCC ACT ATG GTA GCA GCC TTC CAA CTG ATT CGC AAG GGC CAG GAT CCG ATC CAA CCG CGC GAC GAT CTT CCA TTC GCG TCG AAC TTT CTT TAC ATG CTG ACT GAG CAG GAA CCT GAT CCT CTG GCG GCT CGC ATC TTC GAT GCA TGT CTG ATC TTA CAT GCT GAG CAT TCT CTG AAC GCA AGC ACG TTC TCG GCG CGC GTC ACT GCT TCT ACT TTG ACT GAC CCC TAC GCC GTC GTG GCG AGC GCA GTT GGC ACG TTG GCT GGC CCA CTG CAC GGT GGC GCA AAC GAG GAT GTC CTG GCC ATG TTG GAG GCT ATC GGG AGT GCA GAC CAG GTG GAA CCG TGG TTG GAC CGT GCA ATC GCC CAG AAA CAA AAG ATT ATG GGT TTT GGT CAT CGC GAG TAC AAG GTG AAA GAC CCT CGC GCC GTG ATT TTA CAG GGC TTA GCG GAA CAG CTT TTC CAC CGT TTC GGG CAC GAC CCC TTG TAT GAC TTA GCG CGT AAA CTG GAA GAA GCT GCG GCA GAA CGC TTA GGC CCG AAA GGT ATC TAC CCG AAC GTA GAT TTT TAT TCC GGC TTG GTC TAC CGT AAG TTA GGG ATC CCG CGC GAT CTT TTT ACA CCT ATT TTT GCC ATC GCC CGC ACA GCA GGT TGG CTT GCG CAC TGG AAG GAG CAA TTA GGT GCA AAC CGC ATC TTC CGT CCA TCA CAG ATC TAC ACA GGA CCT GTA CCC CGC GAC TGG GTC CCC CTT GAA GCC CGT |
| *Cyanothece sp.* SIO1E1  NET35916.1 | ATG ACA TTC TGT GAA TAT AAA CCC GGG CTG AAG GAA TTC CGG CGA CCC AAA GTT CAG TAA GTT ATG TTG ATG GTC AGC GTG GAA TCC TGG AGT ATC GCG GCA TCC AAA TCG AAG CAC TTG CCG CTA AGT CAA ATT TCC TTG AGA CAG CCT ACC TTT TGA TCT GGG GTG GTT TAC CTA CTC ATG AGG AGT TGG CGT CAT TTG AAT CGG AAA TTC GCT ACC ACC GTC GTT TGA AGT ACC GTA TCC GCG ACA TGA TGA AGT GCT TTC CGG AAA GCG GTC ACC CGA TGG ATA GCC TGC AGG CGT GTG CAG CCG CCC TGG GTC TGT TCT ATT CGC GTC GCG CAC TTG ATA ACC CTG TTT ATA TTC GCG CTG CGG TAG TTC GTC TGC TTG CCA AAA TCC CTA CTA TGG TAG CTG CTT TTC AGA TGA TGC GCA AAG GAA ATG ACC CCA TCC AAC CGC GCG ACG ATC TGA GCT ACT CTG CCA ACT TTC TGT ATA TGC TTA ATG AAC GTG AAC CGG ATC CGT TAG CTG CTC ACA TCT TCG ATG TGT GTC TTA CGC TTC ATG CCG AGC ATA CTA TCA ACG CGT CAA CCT TTT CCG CAA TGG TGA CCG CCA GTA CAC TTA CAG ATC CCT ACG CGG TTG TCG CGT CAG CCG TGG GTA CAT TAG CAG GAC CCC TTC ATG GAG GCG CGA ATG AAG AGG TGC TTT CTA TGC TGG AGG AAA TTG GAA GCG TGG GTA ATG TGC GCC CGT ACT TGG AAG ATT GTC TGC AGC GCA AAG CGC GCA TTA TGG GAT TCG GTC ACC GCG TGT ACA AGG TCA AAG ATC CAC GCG CCA TTA TTC TGC AAG ACC TGG CAG AAC AGT TGT TTG AAA AGC TTG GTG GCG ATC GTT ACT ACG ACA TTG CAG TAG AGC TTG AGC GCC AGG TCT CTG AAA AAT TGG GGC ACA AAG GCA TCT ATC CAA ACG TCG ACT TTT ATT CGG GAT TGG TCT ATC GTA AGT TGG GTA TTC CCC GCG ATA TGT TCA CGC CGG TCT TCG CTA TTG CCC GTG TTG CTG GGT GGT TGG CGC ATT GGA AGG AGC AAC TTG CTG AGA ACC GCA TCT TTC GTC CGA CTC AGA TCT ACA CTG GAC CTC GCC ACA TTC CTT ATG TAG CTA TTG CTG ATC GTC ATC CAC CCC AGG AGG AGG TCA TTC TTA CTA ATT TAC TTG CAG ATT |
| *Gemmata obscuriglobus*  WP_010038645.1 | ATG AGT ACT GAA ATT GAG TAT AAG CCT GGC TTG GAG GAT GTT CCT GCA GCA AAA AGT GCC GTA TCC TTC CTG GAC GGG AAG AAA GCC GTT TTA GAG TAT CGC GGT ATC CCA GTT GAA GTG CTG GCT AAG GAG TCG TCG TTC GAG GAG GTT TCA TGG TTA TTG GTC AAA GGG GAT TTA CCA ACA CAA AAG CAG CTT GCT GAA TTC GAC CAC GAT CTG CGT CAG CGC CGT GCC ATC CAC TTC CGC CTG AAA GAT CTT ATT AAA TGT ATG CCT GCT GAT GGC CAC CCG ATG GAT GCC CTG CAT GCT GGG GTC GCT GCG CTG GGC ATG TTT TAC CCA TGT CCT ACA GTC TCC AAT CCA GCC AAG AAT TGG GAT GCA ACC TGC CGC CTT ATT GCT GCC CTG CCA ACT CTT GTG GCC GCC TTC GCT CGC GTT CGC CGT GGA GAG GAG ATT TTA GAT CCA CGT AGC GAC CTT GAC CAC GCT GGG AAC TTT TAT TAC ATG TTA TTC GGT AAG GAG CCT TCA CCT GCA ACT CGC AAA GTC TTA GAT GCC TGC TTG ATC CTG CAT GCA GAG CAT CAA ATG AAC GCA TCA ACT TTC ACA GCT CGT GTT ACA GGT TCC ACG CTT GCT ACG CCG TAC CAT ACT ATT GCC TCT GCA ATC GGT AGT TTA TCA GGG CCC TTA CAT GGC GGG GCT AAT GAA GAG GCA TTA CGC CAG TTT GAG GAA ATT GGA GGT CCC GAA CAG GTA AAA GGA TGG CTT GAC GCA AAA CGC GCC GTG GAT AGC AAA TAC AAA GTG ATG GGT ATG GGA CAC CGC GTT TAT AAA GTA AAG GAT GCG CGC GCA ACG GTT TTA CAG GAG ATC GCG GAA CAT ATG TTT GCT GAG ACT GCC CGT CCT AAG ACT TAT GAA ACG GCA CTG GAG TTA GAG CGC CTT TGC GCT GGT ATT TAC GGC CCA AAG GGA ATC TAT CCC AAC GTC GAT TTT TAT TCA GGA GTG GTT TAT CAG TCA TTG GGT ATT CCC ACC GAT GTG TTC ACT CCT ATT TTC GCT ATC GCC CGC GTA TCT GGG TGG CTG GCA CAT TGG ACT GAG CAG CTT GTT GGC AAT CGT ATT TTC CGT CCG GAA CAA ATC TCG ATC GGA AAA ACA GAT GTG AAA TAC GTG CCG TTA GAG CAG CGC GCC |
| *Gloeobacter violaceus*  WP_011143005.1 | ATG TCA GGG GAG TAT GTG CCT GGA TTA GAA GGC GTA CCA GCA ACA CGT TCA AAT ATC TCG TTT GTG GAC GGA AAA GCA GGT GTG TTA GAG TAT CGC GGT ATT CCG ATC GAT CAG TTG GCC GAG TCA TCT ACA TTT TTA GAA ACA GCT TTT TTG CTG ATT TTC GAT CAT TTG CCT ACG AAG GAC GAA TTA TTG AGC TTC GAA GTT GAG ATT TTA GGC CAT CGT CGT GTA AAA TAT CGC ATT CGC GAC ATG ATC AAA AGC TTC CCC GAG AGT GGC CGT CCC ATG GTC GCC CTT CAG TCT TGC ATC GCG GCG CTT GGC TTA TTT TAC CCC TTG CAG AAC GAA TCG AAA GAA AAA TAC GCC TAC GAT AGT ACT ATC CGT TTA TTA GCT AAA ATG CCC ACC ATG GTA GCC ACC TTT CAT CAA ATG CGC CTG GGG AAC GAT CCC ATC CCA CCA CGC GAT GAT TTG GGG CAT GCC GCA AAC TTC TTG TAT ATG TTG ACG GGA AAA GAA CCG GAC CCG CGT GCA GCT CGT ATC TTT GAC GTT TGC CTG ATG TTG CAT GCA GAA CAC ACG GTC AAT GCT TCT ACC TTC AGC GCC CTT GTT ACG GCA TCA ACT TTG GCA GAC CCA TAC ACT GTA ATC ACC AGT GCG GCA GGA ACA TTG TCC GGC CCT CTG CAC GGA GGT GCG AAC GAG GAA GTC ATT CGC ATG TTA AAG GAA ATC GGC ACC ATC GAA CGT GTA CGC CCA TAC TTA GAA AAT CGT TTA GCT CGT AAA GAA AAG ATC ATG GGC GTC GGC CAC CGC GTA TAT AAG GTG AAA GAC CCA CGC GCA ACG ATT CTT CAA AAC TTA GCT CAA GAG CTT TTT GAT CGC TTC GGC CAC AGC CGT TTA TAC GAC ATT GCC GTG GAA GTG GAG CGC GTT TGC GAC GAG CTT TTG GGT CAA AAA GGA ATC TAT CCG AAT GTA GAT TTC TAT TCA GGG TTG GTC TAT GAG AAA ATG GGG ATT CCG GCT GAT ATG TTT ACA CCG GTC TTT GCG ATT TCC CGC GTC GCC GGA TGG CTG GCA CAT TGG CAT GAA CAG TTG GCG GAC AAT CGT ATT TTC CGC CCA ACG CAA GTT TAT ACT GGC AGC CAT AAC GTG GAG TTC ACG CCC CTT TCG TTG CGC AGC TAC GCA |
| *Mariprofundus aestuarium*  WP_100278409.1 | ATG GTG GAT AGC CAG AAC GCA GAA GAT TCG ATC GCG TGT TTT TCC CCT GGT TTA GCA GGT ATC CCC GTA GCA GAG TCA GCT GTT TCA TAC GTT GAC GGA AAG AAA GGG TAT CTG GAA TAC CGT GGA ATT GAT ATC GAG CAG CTG GCC GAG AAA AGC TCT TTT GAA GAG TCG AGT TAT CTT TTG CTG TAC GGA GAG CTG CCA ACG GCT AAA GAG TTA AAA GCG TTC GAT GAG AAG TTG AAG CAT CAC CGT CGT ATC AAA TTC CGT ATT CGC GAC ATG ATG AAA TGC TTT CCA GAG TCA GCA CAT CCC ATG GAT AGT CTT CAA GCA ACG GTG GCG GCT CTT GGT ATG TTT TAT CCG TTC CCA ACC AAT CTT GAA GGA GAA TTG GAC AAA GAA ACG ATT GAT AGC GTC TGT GTT AAT TTA ATT TCA AAA ATG CCT ACG CTT GTA GCC GCT CAC GCG CGT ATG CGT CAT GGT GAC GAC CCC ATT GCC CCG CGT GAC GAC TTG TCT CAC GCT GCT AAT TTC TAC TAC ATG CTT ACG GGA GAT GAG CCG TAT CCC CTG ACC GAG CGC ATT TTG GAT GTC GCC TTC ATC GTA CAT GCA GAA CAC GAG ATG AAC GCA TCC ACC TTT AGT GCG TTG GTC ACC GCC TCT ACC CAT GCG GAT CCA TAC ACA TCG ATT TCA GCA GCC ATT GGC GCG CTT TCC GGT CCG CTT CAT GGA GGC GCC AAT GAG GAC GTG TTA CAC ATG CTG GAT GAG ATC GGA AGT GTG GAC AAT GTA GAA GAC TAC TTC AAT CGT AAA TTG GCG AAG CGC GAA AAA TTT GCC GGC CTT GGT CAC CGC GTC TAT AAG ACG AAG GAC CCA CGC GCC ACG CTT TTA CAG AAA CTG TAC GTT GAA CTG ACA GAA AAG GTG GGG GTA GAC ACA ACG TAT GAG ATC GCA CGC CAG ATT GAA AAA TTA TCG CGT TCG ACA TTA GGG GCT AAG GGA GTT TGC CCA AAT GTT GAT TTC TAC AGC GGG ATC GTT TAC CGC AAA ATG GGA ATC CCG ACC GAT CTT TTC ACA CCG ATT TTT GCT ATC GCC CGC GTG TCG GGT TGG CTG GCA CAT TGG AAA GAA CAA CTG GGT CAG GGG CGC ATC TAC CGT CCA AGC CAA ATT TAT ACG GGT AAG TAC GGC CAA TCC TAT GTA CCA GTC GAA GAA CGC |
| *Methylophaga sulfidovorans*  WP_091715369.1 | ATG AGC GAC TTT CTT CCT GGG CTT GAA GGG GTT CCA GCT ACC AAA AGC GCA ATT TCT TTC ATT GAC GGG GAG AAA GGC ATT CTT AGC TAT CGC GGT TAT CCT CTG GAG ACG CTT GCT GAA AAT AGC ACT TTC GAG GAA ACG ACG CTT CTG CTT TTA GAT GGC GAG TTG CCG ACG AAG AAG GCG CTT AAT GAC TTT AGC CAG CAA CTG AAG GAC AAT TAT CGC ATC AAG TAT CAC ATT CGC CAA ATG ATG CGT CAT TTT CCT CAT ACA GGA CAT CCG ATG GAT ATG CTT CAG ACA GCC GTT TCC TCT TTA GGC ATG TTT TAT CCC GGC ACT GAA TGC CTG ACT GAC GCC AAC TCC TGC GAA GAC CTG GAC TAT GTT CGC AAT ATG ACA GTG AAT ATT ATT GCA CAG ATG GCC CCA TTA GTG GCG ATG TGG GAA CAT ATC CGT AAT GGA TGG GAC CCT GTT AAC CCA AAG CAT GAC CTT AGC GTC GCC GAG AAC CTG CTT TAC ATG TTC AAT GGG GAA GAG CCG GAT CCT CTG ATG GCG AAA ATC ATG GAT GTG TGC CTG ATC CTG CAT GCC GAG CAT ACA CTG AAT GCT TCT ACC TTT GCC GCG TTA GTA GCT GGG TCA ACA CTG GCG ACC CCA TAC TCC GTT ATC AGT GCG GCA ATC GGG ACA TTG TCG GGT CCA TTG CAT GGT GGA GCG AAT CAG CGT GTC GTT GGC ATG CTG CAG GAG ATT GGG AGC CCG AAG AAC GTC GAA AGT TGG GTA GAC GAA AAA TTA AAA AAC AAG GAA GTT ATT TGG GGA ATG GGT CAC CGC GAA TAT AAG GTC AAG GAC CCG CGC GCG ACT ATT TTG CAT AAA CTG GTA GAG CAG TTG GTA GCC GAA CGT GGG GGT CAC CTT GAC GAT ATG TTT GAC ACA GCC TTA AAA CTT GAG GAG GTT TGC GCT GAT CGT CTT GGG CAC AAA GGC GTT TAT CCT AAT GTA GAC TTT TAT TCT GGC ATC TTG TAT TCG GAA ATG GGT ATC CCC GAG GAC GAG TTT ACG GCC TTG TTC GCT GTG GCT CGC AGC GCA GGA TGG CTG GCT CAT TGG CGT GAA CAG ATT TCA GAT AAT CGT ATC TAT CGT CCT ACC CAA ATC TAT GTC GGC TCC GAT ATG CGC GAT TAC ACA CCA ATC GAA GAG CGC |
| *Myxococcales bacterium*  MAD59944.1 | ATG TCA TTT ACT CCT GGA TTA GCT GGC GTG ATT GCC GCG GAG TCT TCA ATT TCA ACA ATC GAC GGA CAA AAC GGG ATC CTG CGT TAT CGC GGT ATT CGC ATC GAG GAT TTG GCC GAG AAT TCC TCC TTC GAG GAA GTA GCC TAT TTG TTA TTG TTT AAC GCA CTG CCT ACA ACA GAG CAA TTA TCC GCC TTC GAC GCG CGT TTA AAG GAA CAC CGT TCA ATC CCG GAA GGC ATC GTG ACA TTA CTG GGG ACA TTA CCG GAT ACA ACG CAT CCA ATG GTT GCC TTG CAG TCA GCA GTG AGC GCG CTT GCC GCG TAC TAC CCA CAT TTT GAA GTG GAA GAC ACA GTG AAA AAT GAA GAG AGT ATC ATC CGT ATG ATC GCC TGT TTT CCT ACC ATC GTT GCC AGC TTC GAT CGT TTG CGC AAG GGA AAA GAA GTT TTA GCG CCA AAC AGC GAT CTG GAC CAT GCA GCC AAC TTT CTT TAT ATG CTG AAC GGA GAA GTT CCA GAC GAG AAA CAA CGT CGC ATT ATC GAC GTC GCA TTG GTG TTG CAC GCG GAG CAT GGG TTC AAC GCT TCT ACT TTT ACA GGT CGC GTA GTG GGT TCC ACA ACT GCC AAT CCG TAT GGT AGC CTT TCA GCA GCA GTT GGT TCT TTG TCA GGG CCT TTA CAT GGG GGT GCT AAC GAG CGT GTA TTG CAC ATG TTG GCG GAG ATC GAC GGT GGG GTA GAC GGC GTA GAG AGC TGG TTT ACC CAA GCG CAA GCT GAG AAG CGC AAA ATT ATG GGT CTT GGA CAC CGC GTT TAT AAA GTA AAG GAC CCC CGC GCC ACT GTG CTT CAG GGA ATG GCC CGT GAT TTA TTC GCT TCT CAC GGC AGC ACA CCT TTA TAT GAC ATG GCG GCG CGT TTG GAG GAG ATT GCA AAA GAA CGT TTG GGC GAG AAG GGG ATC GCG CCC AAT GTC GAT TTT TAC AGT GGC TTA GTA TAC CAG AAA CTT GGT ATG GAG ATC GAC CTG TTT ACT CCG TTC TTT GGA ATT GCG CGT ATT GCG GGC TGG GGC TCA CAC TGG ATT GAG CAA CTG GGT AAC AAT CGT ATC TAC CGT CCG ACT CAA CTG TAT GTA GGA GCG ACA GAA GCG CAG TAT GTT CCT ATG TCT GAA CGC |
| *Prochlorothrix hollandica*  WP_017713050.1 | ATG TCG ATT GAC GAA TAC AAG CCC GGA TTA GAG GGT GTA CCG GCG ACG TTG TCA TCA ATT TCC TAT GTT GAT GGG CAA AAG GGC GTA CTG GAG TAT CGT GGA ATT CCC ATC GAA CAA CTT GCT CAA AAC TCA AAT TTC CTG GAA ACA GCG TAT TTA CTG ATT TGG GGT ACA CTG CCG AGT ACC GAG GAA TTG ACG AGT TTT GAA CAA GAA ATC TAC CAA CAC CGC CGC CTT AAA TAT CGC ATC CGC GAT ATG ATG AAG TGC TTC CCG GAA AGC GGG CAC CCG ATG GAT GCT CTT CAA TCG TGC GCA GCC GCA TTG GGG CTT TTC TAT GCC CGC CGT GCA CTG GAC GAC CCA GCC TAC ATC CAT CGT GCC GTC GTG CGT TTG TTA GCG AAA ATT CCG ACC ATG GTA GCG GCT TTT CAA CTT ATG CGT AAG GGA AAT GAT CCG GTA CAA CCC CGC GAT GAT CTT AAT TAC GCC GCA AAT TTT CTG TAT ATG CTG AAC GAA AAG CAA CCG GAT CCA TTA GCA GCT CGC ATC TTC GAC GTG TGT TTA ATC CTT CAT GCA GAG CAC ACC ATT AAC GCT TCG ACA TTC TCT GCC ATG GTC ACG GCC AGT ACT TTG ACC GAT CCG TAC GCG GTA ATT GCT AGT GCA GTC GGA ACG TTG GCC GGT CCT TTA CAC GGC GGC GCA AAC GAA GAG GTG CTT GAC ATG TTA GAG AAC ATC GGC ACT GTG GAC CGC GTA GCC AAC TAT GTT GAC GAA TGC ATC GCA ACT AAA AGC CGT ATC ATG GGA TTC GGA CAC CGC GTA TAC AAA GTA AAA GAT CCG CGT GCG ACC ATT TTG CAG GGA TTA GTA GAG CAG CTT TTT GAT GAG TTT GGC TCT GAT TTG TAC TAC GGA ATC GCC CTT GAG CTT GAA CGT GTT GTA AGT GAT CGT TTA GGA CAT AAG GGG ATT TAC CCT AAT GTT GAC TTC TAC TCA GGA TTA GTA TAT CGC AAG CTG GGT ATT CCC ACA GAT TTG TTC ACC CCA GTT TTT GCT ATC GCG CGT GTC GCG GGT TGG CTT GCG CAT TGG AAG GAA CAG TTG GCT GCA AAC CGT ATT TTT CGC CCC ACG CAA ATC TAT ACA GGA AAC CAC GGC CAG TCT TAC GTG AAG TTG GAC GAC CGC TCT ACC CCT TTG CCC CCA GGT CTT CTG GCA CTG GGA CAG GAG TTG |
| *Pseudanabaena biceps*  WP_175355653.1 | ATG GCC ATC GGC GAG TAT AAA CCA GGA TTA GAA GGC GTG CCC GCG ACC CAG AGC AAC ATT TCA TAC GTC GAC GGA AAA GCG GGG CTT CTG GAG TAT CGT GGG ATC CGT ATC GAA GAA TTG TGC GTA CAC AGT AGT TTC TTG GAG ACA TCA TAT CTG TTA ATC TTT GGT GAG TTA CCT ACG TCG GCG AAG TTA AAG GAG TTT GAA GTT GAT ATT ACC CAC CGT CGT CGC ATC AAA TAT CGT ATC CGT GAT ATG ATT AAA TCG TTT CCT GAT AAC GCA CAC CCC ATG GAT GCC TTG CAA ACC AGT GTG GCG GCA CTG GGC ATG TTT TAT CCG TTG GGA GAT TTC CAC GAC GCG GAT TAT ATC TAT CAG GCG ACC GTG CGT CTG CTT GCC AAA GTG CCC ACT ATG GTT GCA GCC TTC CAT ATG ATG CGT CAA GGC AAT GAT CCA GTT ATG CCA CGC GAC GAT CTG GAC TAT GCG TCG AAC TTC CTT TAC ATG CTG AAT GAG AAA GTG CCG GAC CCT TTA GCC GCG CGT ATC TTT GAC GTT TGT CTG ACT TTA CAC GCG GAA CAC ACT GTT AAT GCA AGC ACG TTT GCG GCG TTG GTC ACG GCT TCA ACG TTA ACG GAT CCC TAC GCG GTT ATT ACA TCG GCT ATC GGA ACG TTA GCC GGC CCA CTG CAC GGG GGC GCC AAC GAA CAA GTG ATG ATG ATG TTG GAG GAA ATC GGT TCC GTT GAT AAT GTT ACC GCG TAT TTA GAA CGT AAG ATC GAG CGC AAG GAG AAG CTT ATG GGC TTT GGC CAC CGT ATT TAC AAA GTA AAG GAT CCG CGT GCT ATT GTA TTG CAA GAG TTA GTC CAT AAA ATG TTT GAC CAG TTC GGA CAT GAC CAT TAC TAC GAT ATC GCC TTG GAG TTA GAG AAA CAG GCA TTT GAG AAA CTT TCG TCA AAG GGA ATC CAC CCG AAC GTA GAT TTT TAC TCT GGG TTA GTC TAC AAG AAG CTT GGA ATC CCT AGC AAC TTA TTC ACG ACA ATC TTC GCT ATC GCC CGT GTG CCA GGT TGG CTT GCT CAT TGG AAA GAA CAA TTG AGT GAC AAT CGT TTA TTC CGC CCG ACT CAG GTC TAC ACG GGG CTT CAT GAT GTT ACG TAT TTA CCT ATT GAA CAT CGT |
| *Sandaracinus sp.*  MAQ19445.1 | ATG CTG ATG GCA GAT AAG CCT GAC TAT GTC CCT GGG CTG GCA GGT GTA CCT GCA GCT CGT TCC TCG GTG TGT CTT ATT GAC GGG CAA GTC GGG AAA CTG CAG TAC CGT GGA TAT CCT ATT GAA CAG TTA GCA GAA TCG TGT AGT TAT GAG GAG GTC GTC TAC TTG CTG CTG TTC GGA GAG TTA CCA ACC AAG GCA CAG CTT GAG AGC TTC GAT GCG GAA TTA AAG GAG GAG CGC GGA TTG AAG TTC CGT ATC GTC GAT GTC TTA AAG AAC TTG CCC GAG CGC GGC CAC CCG ATG GAC GCC TTA ACA GCA GCC GTC GCA TGC ATG GGC ATG TTT TAC CCG GGG GAT CAT GTC GAA GAT TTA GAA TTT CGC CGT CTG TGC GCA ATC CGC CTT GTT GCG AAA CTG CCT ACA GTT GTT GCT GCT TGG CAT CGC ATC CGC CGT GGG GAC AAC CCA ATC GAG CCA CGT ACA GAC CTT GGG CAT GCC GCG AAC TTC CTG TAC ATG CTG GAG GGA CAT GAG CCT GAT GAA TTA GAG GCC AAA GTC ATG GAT GTC GCC TTG ATT TTG CAT GCT GAA CAT TCC ATG AAC GCA TCT ACT TTC ACC GCG CGT GTA ACC GGT AGC ACA TTG TCA GAC CCG TAT GCA GCA GTT GCT AGC GCA ATC GGT AGC CTT GCT GGC CCA CTG CAT GGT GGG GCT AAC GAG CGC GTT CTG GAG ATG TTA CGC ACG ATC GAA GAT TCC ACA TCG GAG GCC GTG CGT CGC TGG GCA GAA GAC AAA CTG GCA CGC AAA GAG AAA ATC ATG GGC TTT GGA CAT CGC GTC TAT AAG GTT AAA GAC CCA CGT GCT AAC ATT TTG CAG AAA TTA GCC ACG CAA CTG TTT GAG AAG CGT GGG CGC ACA CCT ATC TAC GAC ATT GCA GTG GAG TTG GAA AAG CAA ATG GGA GAA TTA GTG GGT CAT AAG GGA ATT GCA CCG AAT GTT GAC TTT TAT AGC GGA ATC GTT TAC GAA AAA ATG GGC ATC CCA GTT GAT TTG TTT ACT CCT GTC TTT GCG ATT GCC CGC GTC GGA GGT TGG CTG GCC CAC CTT CTT GAA CAG TTG CAA GAC AAT CGT ATC TTT CGC CCG ACG CAA ATT TGG ATT GGT GAG GCC GAC CGC ACC GTA CCA CCT CTG GCT GAT CGT GGG |
| *Synechococcus elongatus*  BAD79102.1 | ATG ACT GCC GTC AGC GAG TTT CGG CCT GGC CTA GAA GGC GTG CCC GCC ACA CTC TCG AGC ATT AGC TTT GTC GAT GGC CAG CGC GGC GTC CTA GAG TAT CGC GGC ATC AGC ATC GAG CAA CTG GCG CAA CAG AGC AGT TTT CTG GAA ACC GCC TAC CTG TTG ATT TGG GGC CAT CTA CCA ACT CAG CAG GAA TTG ACC GAG TTC GAG CAC GAA ATT CGC TAC CAC CGC CGC ATC AAG TTC CGC ATC CGG GAC ATG ATG AAA TGC TTC CCC GAT AGC GGC CAT CCT ATG GAT GCC CTG CAG GCG AGC GCC GCA GCC CTC GGG TTG TTC TAT TCG CGG CGC GCC TTG GAT GAT CCC GAA TAC ATT CGG GCG GCC GTT GTG CGT TTG CTA GCC AAA ATT CCG ACG ATG GTG GCT GCC TTC CAG CTG ATC CGC AAG GGT AAC GAC CCA ATT CAG CCC CGC GAT GAA CTG GAC TAC GCC GCC AAC TTT CTC TAC ATG CTG ACG GAG CGC GAG CCC GAT CCA GTC GCA GCT CGG ATT TTT GAT ATT TGC CTC ACC CTG CAC GCC GAA CAT ACG ATC AAC GCC TCG ACC TTC TCG GCG ATG GTC ACA GCT TCG ACC CTG ACC GAT CCC TAC GCT GTC GTT GCT TCT GCC GTT GGC ACC TTG GCT GGC CCC CTC CAT GGC GGC GCC AAT GAA GAA GTG CTG GAC ATG CTG GAG GCG ATC GGT TCC GTC GAG AAT GTT GAG CCC TAC CTC GAC CAC TGC ATT GCC ACC AAA ACG CGC ATT ATG GGC TTT GGG CAC CGT GTC TAC AAA GTC AAG GAT CCG CGG GCA GTC ATT CTG CAA AAT CTG GCC GAG CAA CTG TTC GAT ATC TTC GGC CAT GAT CCC TAC TAC GAA ATC GCG GTC GCA GTC GAA AAG GCA GCA GCC GAG CGA CTC AGC CAC AAG GGC ATT TAC CCC AAC GTC GAT TTC TAC TCC GGC TTG GTC TAT CGC AAG CTC GGT ATT CCT AGC GAT CTA TTC ACA CCG GTG TTT GCG ATC GCG CGG GTT GCG GGC TGG CTC GCC CAC TGG AAA GAG CAG CTG AAC GAA AAT CGG ATC TTC CGC CCC ACT CAG ATC TAC ACG GGC AGC CAC AAC CTC GAC TAC ACC CCG ATC GCC GAT CGG GAT TTG GCG ATC GAA TCT GAT |
| *Synechocystis sp.*  WP_193386667.1 | ATG AAT TAT ATG ATG ACT GAT AAC GAA GTG TTT AAA GAA GGC CTA GCC GGA GTC CCC GCC GCT AAA TCG AGG GTG AGC CAT GTG GAT GGC ACC GAC GGT ATT TTG GAG TAC CGG GGC ATT CGC ATC GAA GAA TTA GCC AAA TCC AGT AGT TTT ATC GAA GTA GCC TAT CTG CTC ATC TGG GGT AAA TTG CCC ACC CAG GCA GAG ATC GAA GAG TTT GAG TAC GAA ATT CGC ACC CAT CGA CGC ATT AAG TAC CAC ATC CGG GAC ATG ATG AAA TGT TTT CCC GAA ACA GGG CAC CCC ATG GAT GCC TTG CAA ACT TCA GCG GCG GCC TTG GGA TTG TTC TAT GCT CGA CGG GCC TTG GAT GAC CCC AAA TAT ATC CGG GCG GCG GTG GTG CGT CTG TTA GCC AAA ATC CCC ACC ATG GTG GCA GCT TTC CAC ATG ATC CGG GAG GGT AAC GAT CCC ATT CAG CCC AAT GAT AAA TTG GAT TAC GCT TCC AAC TTC CTT TAC ATG CTG ACG GAG AAG GAG CCA GAC CCC TTT GCC GCC AAA GTG TTT GAT GTG TGT TTG ACC CTC CAT GCT GAG CAC ACC ATG AAT GCG TCC ACC TTT TCG GCC CGG GTA ACG GCT TCT ACT CTC ACG GAT CCC TAT GCA GTG GTT GCC TCG GCG GTG GGG ACT TTG GCG GGG CCG CTC CAC GGG GGA GCC AAC GAA GAA GTG CTA AAT ATG CTT GAA GAA ATT GGC TCA GTG GAA AAT GTC CGC CCC TAC GTG GAA AAA TGC CTG GCC AAC AAA CAG CGC ATC ATG GGC TTT GGC CAC CGA GTT TAT AAA GTC AAA GAC CCC CGG GCA ATT ATT TTG CAG GAT TTG GCT GAA CAG TTA TTT GCC AAA ATG GGC CAC GAC GAA TAT TAC GAA ATC GCA GTG GAG TTG GAA AAA GTA GTG GAA GAA TAC GTG GGT CAA AAG GGC ATT TAC CCC AAT GTG GAC TTC TAT TCC GGT TTG GTT TAC CGC AAG CTA GAC ATC CCC GCC GAT CTG TTT ACG CCC CTA TTT GCG ATC GCC AGG GTG GCG GGT TGG TTG GCC CAC TGG AAG GAA CAA TTA TCA GTC AAT AAA ATT TAC CGT CCT ACC CAA ATT TAC ATC GGT GAC CAT AAT TTA TCT TAT GTT CCC ATG ACA GAA CGG GTA GTT TCC GTG GCC CGC AAT GAA GAC CCC AAT GCG ATT ATT |
